# Cold Orthogonal Translation: A Psychrophilic Pyrrolysyl‐tRNA Synthetase Boosts Genetic Code Expansion in *E. coli*

**DOI:** 10.1002/advs.202513600

**Published:** 2026-04-15

**Authors:** Nikolaj G. Koch, Peter Goettig, Michael A. Nash, Juri Rappsilber, Nediljko Budisa

**Affiliations:** ^1^ Bioanalytics Group Institute of Biotechnology Technische Universität Berlin Berlin Germany; ^2^ Biocatalysis Group Institute of Chemistry Technische Universität Berlin Berlin Germany; ^3^ Department of Chemistry Institute of Physical Chemistry University of Basel Basel Switzerland; ^4^ Department of Biosystems Science and Engineering ETH Zurich Basel Switzerland; ^5^ Department of Pharmaceutical and Medicinal Chemistry Institute of Pharmacy Paracelsus Medical University Salzburg Austria; ^6^ Wellcome Centre for Cell Biology University of Edinburgh Edinburgh Scotland UK; ^7^ Chemical Synthetic Biology Group Department of Chemistry University of Manitoba Winnipeg Manitoba Canada

**Keywords:** bioorthogonal chemistry, genetic code expansion, noncanonical amino acids, psychrophilic enzymes, synthetic biology

## Abstract

Orthogonal translation systems (OTSs) enable site‐specific incorporation of non‐canonical amino acids (ncAAs) and are central to genetic code expansion. Current engineering strategies typically rely on hyperstable aminoacyl tRNA synthetase (aaRS) scaffolds to tolerate destabilizing mutations required for substrate diversification. Here, we introduce an alternative design principle: exploiting the intrinsic conformational flexibility of a psychrophilic pyrrolysyl‐tRNA synthetase (PylRS) enzyme to enhance mutational tolerance and in vivo performance. We identified a cold‐adapted PylRS from *Methanococcoides burtonii* and established a psychrophilic OTS (“Cold‐OTS”) compatible with *Escherichia coli*. This system consistently outperformed established mesophilic and thermophilic PylRS variants in single‐ and multi‐site ncAA incorporation. Notably, Cold‐OTS maintained high suppression efficiency at low ncAA concentrations and exhibited enhanced absolute performance at reduced cultivation temperatures, even under globally diminished protein synthesis rates. In addition, engineered variants accommodated a broader set of substrates, consistent with elevated substrate promiscuity. These findings establish psychrophilic aaRS scaffolds as potentially powerful resources for genetic code expansion. Given the broad host compatibility of the PylRS platform, Cold‐OTS provides a scalable strategy for efficient production of ncAA‐modified proteins across diverse biological systems.

## Graphical abstract

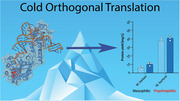

## 1 Introduction

New‐to‐nature non‐canonical amino acids (ncAAs) expand the functional repertoire of proteins beyond the limits of the canonical genetic code [1, 2, 3, 4]. Their specific incorporation relies on orthogonal translation systems (OTSs), typically composed of engineered orthogonal aminoacyl tRNA synthetase (aaRS)/tRNA pairs that function independently of the host's endogenous translational machinery. To date, more than 500 ncAAs have been genetically encoded, with over 300 achieved using pyrrolysyl tRNA synthetase (PylRS)‐based systems alone [1, 2, 3, 4, 5]. Among available strategies, genetic code expansion (GCE) by amber stop codon suppression remains the most widely applied approach, enabling ncAA incorporation at predefined sites during ribosomal protein synthesis. Owing to its natural orthogonality in both prokaryotes and eukaryotes, PylRS has become the most comprehensive platform for genetic code expansion [2, 6, 7].

An ideal OTS must combine in vivo efficiency with sufficient substrate tolerance to permit straightforward engineering. While existing PylRS variants offer notable versatility in their in vivo activity often remains limiting [8]. Numerous strategies have been pursued to enhance cellular incorporation efficiency, including optimization of plasmid architecture and promoter strength [9], engineering tRNA^Pyl ^ [10], rational and semi‐rational aaRS mutagenesis [11], sequence‐context optimization [12], host strain engineering [13], modification of translational factors [14], and solubility enhancement of aaRS variants [15]. Despite these advances, GCE protein yields frequently remain substantially below those of their wild‐type counterparts, underscoring a persistent efficiency bottleneck [16].

A prevailing paradigm in enzyme engineering—including in (GCE)—is to employ (hyper)thermophilic enzymes as starting scaffolds. Their intrinsic stability is presumed to buffer the destabilizing mutations required for active‐site remodeling. The hyperthermophilic tyrosyl‐tRNA synthetase of *Methanocaldococcus jannaschii* (*Mj*TyrRS), for example, exhibits high substrate specificity and typically requires extensive mutagenesis–often up to ten substitutions–to accommodate new substrates [17]. However, such high specificity complicates engineering and reduces the probability of identifying functional variants [17, 18]. Moreover, thermophilic enzymes frequently display reduced catalytic performance under standard laboratory cultivation conditions, compared to their mesophilic homologs, necessitating additional optimization [18, 19, 20].

In contrast, the unique evolutionary history of Pyrrolysine (Pyl) has imposed comparatively weak selective pressure for highly specific substrate recognition by PylRS. Instead, substrate binding is mediated largely through hydrophobic interactions, enabling encoding of diverse ncAAs with relatively few mutations—often one to four substitutions (and never more than three in this study) [21, 22]. This intrinsic permissiveness suggests that extreme scaffold stability may not be a prerequisite for successful PylRS engineering.

Based on this reasoning, we hypothesized that genetic code expansion using PylRS could benefit from a fundamentally different design principle: rather than relying on hyperstable scaffolds to tolerate mutations, exploiting the conformational flexibility of psychrophilic enzymes to enhance catalytic adaptability. Cold‐adapted enzymes typically exhibit increased flexibility and elevated catalytic activity at low to moderate temperatures, albeit often at the cost of thermal stability [19, 23, 24]. Here, we identify and engineer a psychrophilic PylRS from *Methanococcoides burtonii* (*M. burtonii*) PylRS (Mbur) and demonstrate that it outperforms mesophilic and thermophilic homologs in terms of in vivo incorporation efficiency and substrate scope. Notably, its relative activity particularly improves at lower cultivation temperatures (18°C) and under limiting ncAA concentrations.

## 2 Results

### 2.1 Initial Screening of Psychro‐, Meso‐ and Thermophilic PylRS Variants

To address the efficiency limitations of current PylRS‐based systems, we systematically screened psychrophilic mesophilic, and thermophilic PylRS homologs. PylRSs can be broadly divided into two structural classes: the +N class, which contains an N‐terminal domain (NTD), and the ΔN class, which lacks the NTD [2].

**FIGURE 1:**
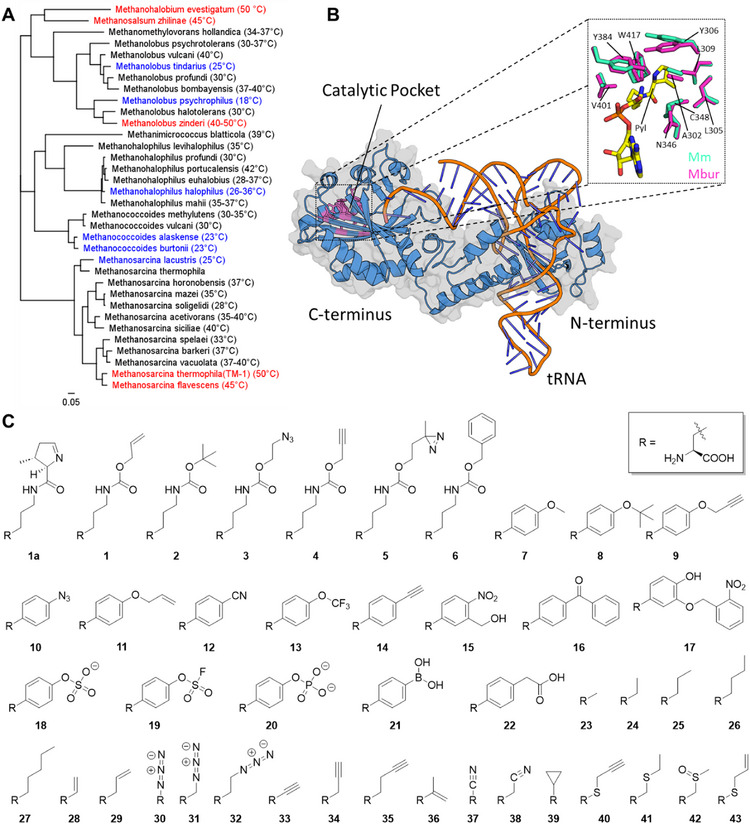
Integrating evolutionary phylogeny, structural biology, and non‐canonical amino acid chemistry led to the discovery of a novel psychrophilic orthogonal translation system (“Cold‐OTS”) based on PylRS. (A) Phylogenetic tree of the +N PylRS class generated using the approximate maximum‐likelihood method. The FastTree algorithm (version 2.1.11) was used to construct the tree based on sequences available from NCBI and *M. alaskense* from the JGI database [25]. Numbers in parentheses indicate the optimal growth temperature (OGT). Coloring denotes psychrophilic (blue, ≤26°C) and thermophilic (red, ≥45°C) PylRS variants. The scale bar corresponds to 5% sequence divergence. (B) Structural of *M. burtonii* PylRS (cyan, linker omitted) shown as a cartoon representation. The model was generated by aligning the N‐ and C‐termini with a superposition of the corresponding domains from *M. mazei* (PDB ID 5UD5) and *Desulfitobacterium hafniense* (PDB ID 2ZNI) (see Supplementary Information for details). The catalytic pocket with bound Pyl‐AMP (yellow) was taken from *M. mazei* (PDB ID: 2Q7H), while the *M. burtonii* structure was predicted using ColabFold [21, 26]. Conserved active site residues are shown as sticks, with *M. burtonii* PylRS in red and *M. mazei* residues in blue (numbering according to *M. mazei*). The linker region was omitted for clarity but is shown in the Supplementary Information. (C) Non‐canonical amino acid (ncAA) substrates used in this study. Systematic and trivial names are provided in Table S3. Compounds **1**, **3**, **4**, **9**–**11**, **14**, **23**–**30**, **35**, and **38** are compatible with bioorthogonal click reactions, including 1,3 dipolar cycloaddition, inverse electron‐demand Diels–Alder, and photocatalyzed thiol‐ene coupling [27].

To prioritize candidates within the +N PylRS set, we chose variants according to their optimal growth temperature (OGT) (see Figure 1A and discussion in Supporting Information S1: Section 1.1 for details). The psychrophilic subset comprised four PylRS variants derived from organisms with OGT of 26°C or lower. These were compared to two mesophilic and two thermophilic +N PylRS homologs.

Several ΔN‐class PylRS variants have been reported to exhibit high activity, [28, 29], in some cases comparable to *Methanosarcina mazei* (Mm) [30]. Although no psychrophilic ΔN PylRS has yet been identified, our objective was to determine the most efficient variant overall. We therefore included 15 high‐performing ΔN PylRS variants and their corresponding tRNAs, selected from a recent comprehensive study [31] to ensure representation across all ΔN subgroups (A, B, C, and S). In addition, the thermophilic *Methermicoccus shengliensis* (Sheng, OGT 65°C) was included for comparison [32].

In total, 23 PylRS variants were evaluated for in vivo activity using six different Pyl analogs (**1**, **2**, **3**, **4**, **5**, **6**), selected to represent a spectrum of known substrate efficiencies (Figures S5 and S6). Based on prior activity data for the +N class, substrates were categorized as high‐activity (**1**, **2**) [33, 34], moderate (**3**, **4**, **5** [35]), and low‐activity (**6)** [34], the latter exhibiting minimal incorporation even at 3.5 mM concentrations. To ensure optimal performance of the benchmark system, the SmbP‐tag to wild‐type *Methanosarcina barkeri* (Mb) PylRS variant—previously shown to enhance activity—was used throughout as a reference construct [15]. Additional structural and compositional analyses of +N variants are provided in Supporting Information S1: Sections 1.2–1.4).

To exclude cytosolic enzyme abundance as a confounding factor, we quantified intracellular PylRS levels, under the same conditions as our target protein production setup, using a split‐GFP protein‐fragment complementation assay (Figure S9). This method enables detection of soluble proteins in living cells and allowed direct comparison of variant expression levels [36, 37, 38]. Because several ΔN PylRS variants were incompatible with the original pTECH backbone, all constructs for the split‐GFP analysis were cloned into a modified pTECH system containing an inducible cuminic acid promotor (P_CymRC_) [39] with expression strength comparable to the original *lpp* promotor.

**FIGURE 2:**
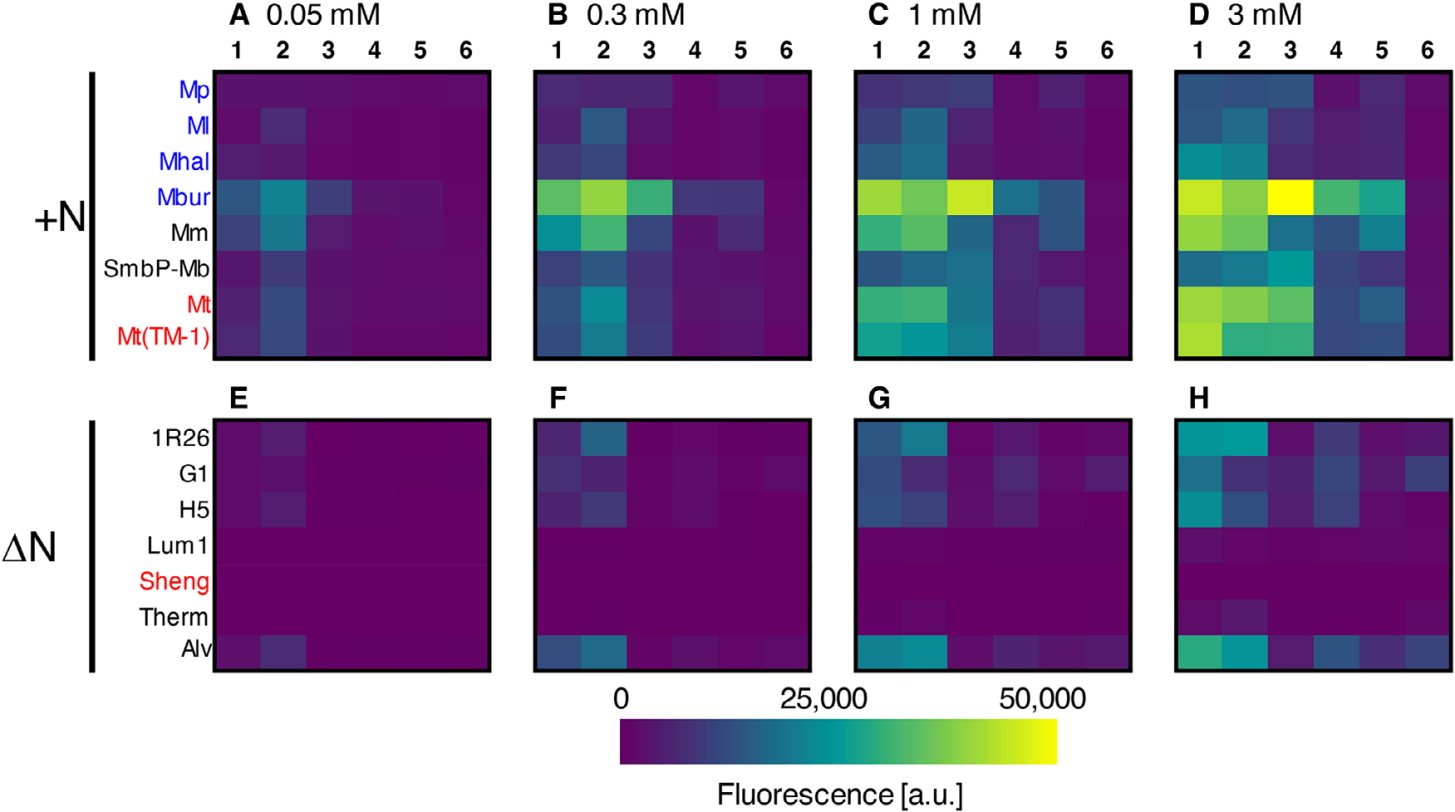
Expression normalized heatmaps showing the fluorescence from sfGFP(1x amber) expression in *E. coli* BL21(DE3) for the selected PylRS variants. The ncAA concentrations supplied in panels (A) and (E), (B) and (F), (C) and (G), and (D) and (H), were 0.05, 0,3, 1, and 3 mM, respectively, as indicated in the figure. Complete activity heatmaps for all PylRS variants, additional ncAA concentrations, and corresponding expression data are provided in the Supplementary Information (Figures S5, S6, and S10). Substrate numbering corresponds to Figure 1. PylRS abbreviations are defined as follows: Mp = *Methanolobus psychrophilus*; Ml = *Methanosarcina lacustris*; Mhal = *Methanohalophilus halophilus*; Mbur = *Methanococcoides burtonii*; Mm = *Methanosarcina mazei*; SmbP‐Mb = SmbP fusion with *Methanosarcina barkeri*; Mt = *Methanosarcina thermophila*; Mt(TM‐1) = *Methanosarcina thermophila* type strain TM‐1 (DSM 1825); 1R26 = *Candidatus Methanomethylophilus* sp. 1R26; G1 = methanogenic archaeon mixed culture ISO4‐G1; H5 = methanogenic archaeon ISO4‐H5; Lum1 = *Methanomassiliicoccus luminyensis*; Sheng = *Methermicoccus shengliensis*; Therm = *Candidatus Methanohalarchaeum thermophilum*; Alv = *Candidatus Methanomethylophilus alvus*.

The sfGFP(11) fragment was fused to the NTD of each PylRS variant via a flexible (GGGGS)_3_ linker. Consistent with previous reports that both +N and ΔN PylRS enzymes tolerate N‐terminal tagging better than C‐terminal domain fusions [40, 41], we confirmed compatibility for the Mbur variant (Figure S7). Detailed validation of promotor strength and sfGFP(11)‐linker design is provided in Supporting Information S1: Sections 1.6–1.7). Expression normalized in vivo activity for substrates **1**–**6** is shown in Figure 2. Variant I2 was excluded from further analysis because only 12.7% of cells shifted to the positive split‐GFP population, indicating markedly reduced soluble expression. In contrast, all other variants exhibited 70–90% of gated cells within the positive population (Figure S101). Statistical comparison revealed that ΔN PylRS variants were, on average, expressed at significantly higher levels than +N variants (Figure S10).

The final expression‐normalized in vivo activity heatmaps are presented in Figure 2. All selected +N PylRS variants were functional under standard cultivation conditions, albeit with varying activity levels (Figures S5 and S6). As expected, substrate **6** (low‐activity control) yielded minimal incorporation, even at the highest ncAA concertation tested (9 mM; Figure S5). Importantly, none of the psychrophilic PylRS variants exhibited elevated background incorporation, demonstrating fidelity toward canonical amino acids (cAAs) comparable to established PylRS systems.

Among the four tested psychrophilic PylRS variants, Mbur emerged as a clear outlier, consistently demonstrating superior OTS efficiency for all substrates across all tested concentrations. Maximal efficiency for substrate **2** was achieved at just 0.3 mM. Overall, Mbur displayed exceptional performance at lower ncAA concentrations, indicating a more efficient OTS. For example, at 0.05 mM substrate **2**, Mbur exceeded other variants by approximately 30%. At 0.3 and 1 mM, Mbur exhibited between 300% (for **3**) and 400% (for **4**) higher efficiency than the second‐best variant.

The two mesophilic PylRS variants showed mixed performances. Mm displayed higher efficiency than SmbP‐Mb at low ncAA concentrations, consistent with previous reports [11]. However, SmbP‐Mb outperformed Mm at high concentrations (3 and 9 mM) of ncAA **3**. The thermophilic Mt(TM‐1) variant performed similarly to Mm at ncAA concentrations of 1 mM and above. In contrast to highly thermophilic enzymes that often show little to no activity at 37°C, Mt(TM‐1) maintained measurable activity under these conditions, likely reflecting its origin from a mildly thermophilic organism.

Of the 15 ΔN PylRS variants evaluated, the five constructs (Nitra, RumEn, Sheng, Tron, and Clos) showed no detectable activity in our OTS setup (Figure S6). Notably, our pTECH‐based OTS setup, consistently outperformed the widely used pUltra platform in direct comparison [9] (Figure S12), suggesting that active variants should have been detectable under these conditions. From the 15 ΔN PylRS variants, we selected the seven best performing ΔN PylRS constructs for inclusion in Figure 2 (lower half), together with the thermophilic Sheng variant. As shown in Figure 2, even the best‐performing ΔN constructs (1R26, G1, H5, and Alv) exhibited, at most, only half the in vivo activity of the Mbur construct—and this was observed only at ncAA concentrations of 1 mM or higher (for substrates **1**, **2,** and **4**). Other constructs, such as 030, Deb, and Int, displayed minimal activity and required very high ncAA concentrations to generate signals distinguishable from background suppression (Figure S6).

### 2.2 Efficiency Characterization of Selected PylRS Variants by Increasing the Number of ncAAs Incorporated

As discussed in a recent review [2], using an *E. coli* strain with release factor 1 (RF1) and suppression of a single stop codon (as in Figure 2) provides a basic indication of the potential in vivo efficiency of an OTS. However, the dynamic range of the reporters used in normal strains is often too narrow to fully capture efficiency differences, particularly when reporter constructs containing multiple stop codons are included (see Figures S19 and S20). This limited dynamic range makes it difficult to appropriately resolve efficiency differences.

To enhance discriminatory power, we employed an RF1 knock‐out strain in conjunction with reporter constructs containing 1, 3, and 5 stop codons [42]. In the absence of release factor 1 amber codons are no longer efficiently recognized as termination signals, enabling more efficient multiple site‐specific ncAA incorporations within the same protein. Under these conditions, suppression efficiency differences are amplified across successive incorporation events. Consequently, both the absolute reporter signal (peak y‐value) and the relative line spacing between reporter constructs provide a more sensitive measure of OTS performance.

**FIGURE 3:**
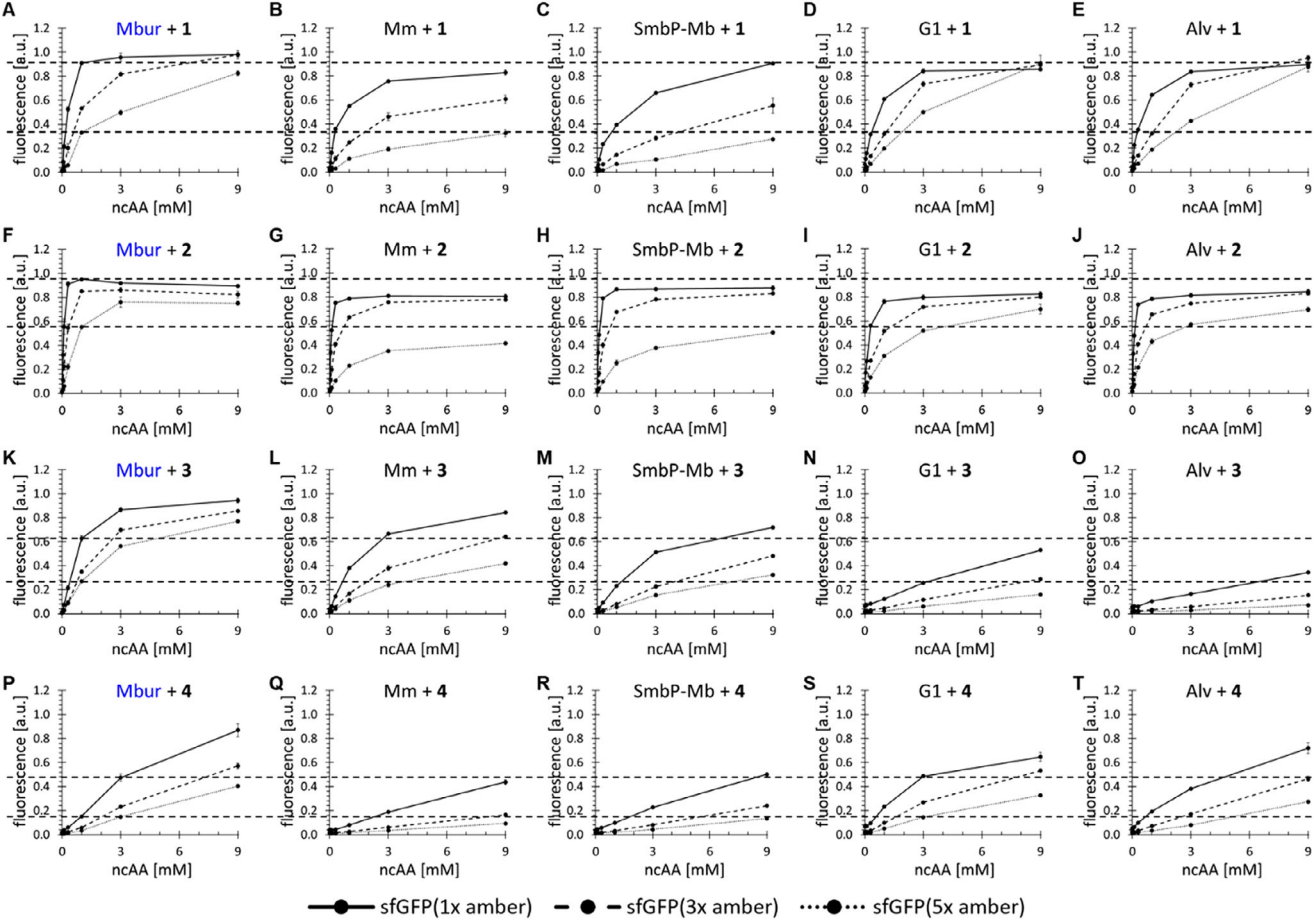
Concentration‐dependent sfGFP production using the two best‐performing +N and ΔN PylRS variants from Figure 2, compared with Mbur. Protein expression was performed in RF1‐deficient *Escherichia coli* B‐95.ΔA. Endpoint measurements were performed at ncAA concentrations (x‐axis) of 0.05, 0.1, 0.3, 1, 3, and 9 mM. Fluorescence values were normalized to the corresponding wild‐type sfGFP reporter constructs (without an in‐frame stop codon). Error bars represent the standard deviation of three biological replicates (*n* = 3). Fluorescence intensity (y‐axis) is reported in arbitrary units (a.u.). The upper dashed line indicates the reference condition corresponding to Mbur with 1× amber at 1 mM ncAA. The lower dashed line indicates the Mbur/5x amber condition. For substrate **4**, the upper dashed line corresponds to 3 mM, due to reduced incorporation efficiency. Substrates and PylRS constructs are indicated in the panel headings. A zoomed‐in figure showing the background suppression can be found in the SI (Figure S14B).

We selected the four best performing +N and ΔN variants and tested their ability to perform multiple ncAA incorporations using substrates **1**,**2**,**3,** and **4,** in the B‐95.ΔA strain (Figures S13 and S14). From this dataset, we identified the two best‐performing +N and ΔN variants, alongside the *Mbur*, for further analysis (Figure 3). All constructs performed best with substrate **2**, except for H5, which showed slightly better performance with substrate **1** when suppressing a single stop codon. For substrate **2**, Mbur clearly outperforms the other variants, particularly with the 5x amber construct. Mbur achieved near wild‐type signal levels and exhibited only ∼20% signal reduction at 3 mM, whereas the other variants showed ∼40%–50% signal loss under the same conditions (Figure 3F–J).

For substrate **1**, the two ΔN variants (G1 and Alv) perform comparably to Mbur at 3 mM but were clearly outperformed at lower concentrations, as shown on both single and multiple ncAA incorporation experiments (Figure 3A–E). For example, at 1 mM, the two ΔN variants reached only 60% of the wild‐type signal, whereas Mbur exceeded >90%.

For substrate **3**, Mbur demonstrated significantly better performance in terms of both wild‐type signal levels and efficiency at lower concentrations. For substrate **4,** G1 and Alv are clearly better than Mm and SmbP‐Mb and perform on par with the Mbur until reaching 9 mM where the Mbur showed a higher maximum signal (Figure 3P–T). This observation is consistent with the overall trend in Figure 2, where substrate **4** consistently showed lower efficiency across +N variants. Collectively, these data establish Mbur as the most robust scaffold under increasing incorporation demand, particularly at reduced substrate concentrations. In the absence of RF1 (B‐95ΔA strain), background suppression is inherently elevated; this is reflected in the 0 mM ncAA condition shown in Figure 3. Notably, Mbur exhibited the lowest background suppression, whereas the ΔN variants showed consistently higher baseline levels (Figures S14B and S15).

### 2.3 Flow Cytometry Analysis of the Best Performing PylRS Variants for Temperature‐Dependent Performance

In recombinant protein production with *E. coli*, many target proteins are either sequestered into inclusion bodies or fail to fold correctly. A common strategy to mitigate these issues is to slow down the protein production of *E. coli* by lowering the cultivation temperature [43]. While this approach might alleviate problems with specific target proteins, it may also reduce protein yields in the context of GCE due to decreased activity of the OTS.

**FIGURE 4:**
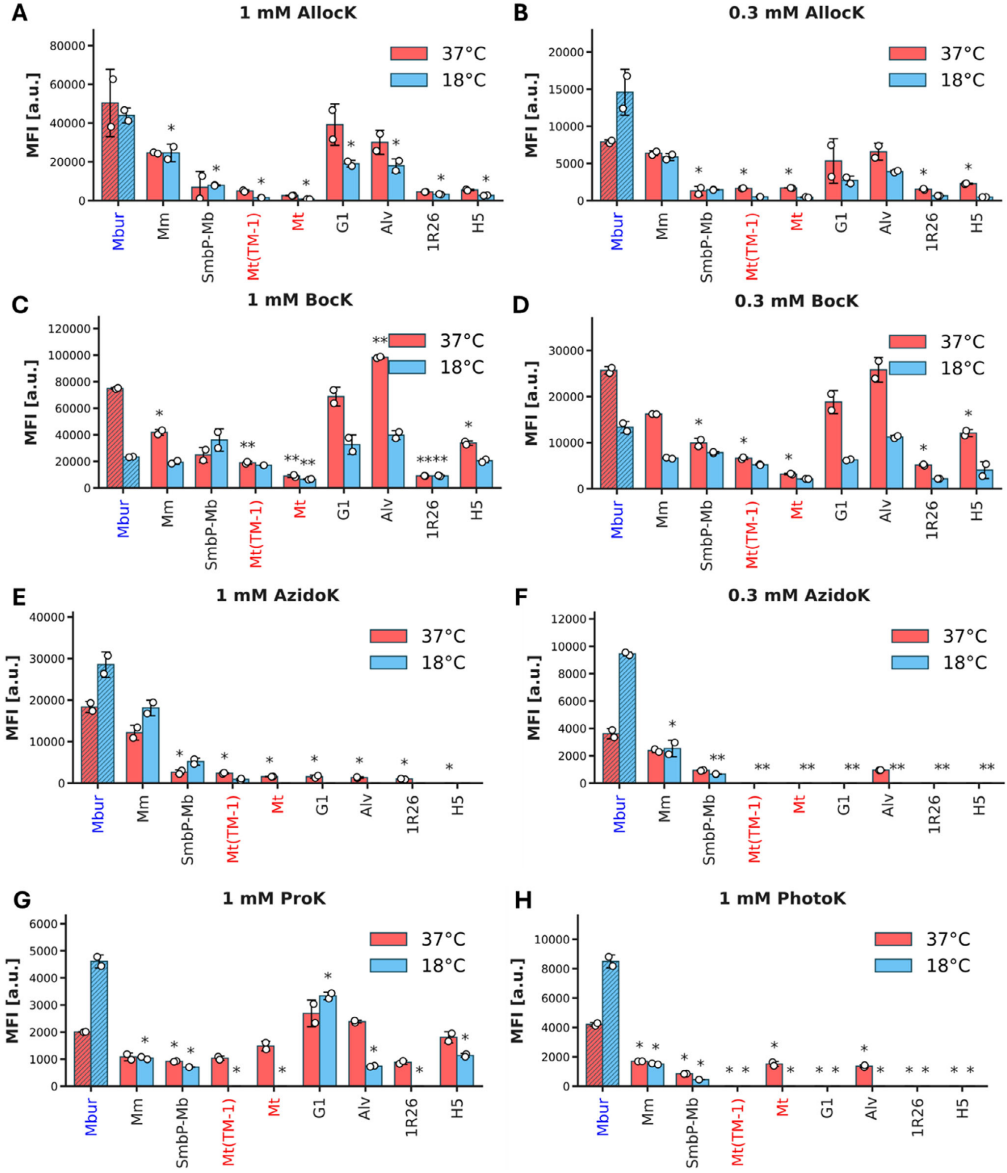
Temperature‐dependent production of sfGFP(5x amber) in the B‐95.ΔA strain at 37°C(red columns) and 18°C(blue columns), supplemented with different ncAA concentrations. Panels show results for: (A) 1 mM **1**, (B) 0.3 mM **1**, (C) 1 mM **2**, (D) 0.3 mM **2**, (E) 1 mM **3**, (F) 0.3 mM **3**, (G) 1 mM **4,** and (H) 1 mM **5**. Constructs were analyzed by flow cytometry in biological duplicates (*n* = 2), and the median fluorescence intensity (MFI) of the target population is shown. Missing bars indicate fluorescence levels not exceeding background suppression, meaning that no distinct expression populations could be resolved in the dot plots. Welch's t‐tests comparing Mbur (striped) to all other constructs within each temperature group were performed. BH/FDR‐adjusted *P*‐values are indicated as follows: *p < 0.05, **p < 0.01, ***p < 0.001. Representative dot plots for the Mbur variant are provided in Figures S98–S100. The background suppression and wild‐type sfGFP performance for each variant are found in Figure S15.

Given that Mbur originates from a psychrophilic organism, we aimed to determine whether its performance differs from other PylRS constructs at lower cultivation temperatures. To test this, we selected the four next best‐performing +N and ΔN constructs, alongside Mbur (Figure 4). We used the sfGFP(5x amber) reporter construct, which provides a robust dynamic reporter signal range, and tested substrates **1**–**5** to obtain a comprehensive performance profile. For substrates **1**–**3** (higher‐performing substrates), activity was measured at both 1 and 0.3 mM to assess concentration‐dependent effects.

Across all substrates and temperatures, Mbur consistently ranked as the top‐performing variant or among the top three (together with G1 and Alv for ncAA substrate **2**). For substrate **2**, which previously demonstrated superior performance (Figure 3), Mbur shows relatively better efficiency at 0.3 to 1 mM, with this advantage being even more pronounced at 18°C. Most notably, for 5 out of these 8 conditions, Mbur exhibited higher absolute performance at 18°C than at 37°C. We observed that for Mbur, the peak signal was reached after 72 h at 0.3 mM concentrations for substrates **1**,**2,** and **3** as well as at 1 mM for substrates **4** and **5**. However, for substrates **1** and **2** at 1 mM, the peak signal was achieved after 48 h. This suggests that substrates with lower recognition benefit from longer cultivation times at lower temperatures. It was also generally observable that at 18°C, the non‐expressing population was almost non‐existent compared to cultures grown at 37°C, a trend observed across all constructs (Figures S98–S100).

It is well established that the overall protein production rates in *E coli* decrease significantly at lower temperatures [43]. In this study, the general protein production rate at 18°C across all constructs was only between 20%‐50% of that observed at 37°C (Figure S15B). Consequently, an absolute increase in performance at lower temperatures was unexpected, as an OTS must compensate for the reduced protein production rate to achieve such an effect. Remarkably, for substrate **3** (at 0.3 mM), and substrates **4** and **5**, Mbur exhibited∼2.5 times higher performance at 18°C compared to 37°C. None of the other constructs showed similar absolute performance increase at lower temperatures, except for *Mm*, which displayed slightly improved performance for ncAA **3** at 1 mM. These findings indicate that the psychrophilic origin of Mbur confers a functional advantage under reduced‐temperature conditions, enabling sustained or enhanced ncAA incorporation despite globally diminished protein synthesis rates.

### 2.4 Investigating the Substrate Promiscuities of Selected +N Variants

As shown in Figure 1 and 4, the ΔN variants exhibit lower substrate promiscuity compared to the +N class, which is why this analysis focuses exclusively on the +N variants. Psychrophilic enzymes are often characterized by lower substrate affinity and specificity, attributed to their increased flexibility, or in some cases, enhanced active site accessibility. These structural features often correlate with elevated substrate promiscuity, a common trait among psychrophilic enzymes [24].

Notably, increased conformational flexibility has previously been shown to enhance aminoacyl‐tRNA synthetase promiscuity in engineered systems [44]. From an enzyme engineering perspective, higher promiscuity is highly desirable as it facilitates the engineering of enzymes for new substrate recognition [45, 46].

**FIGURE 5:**
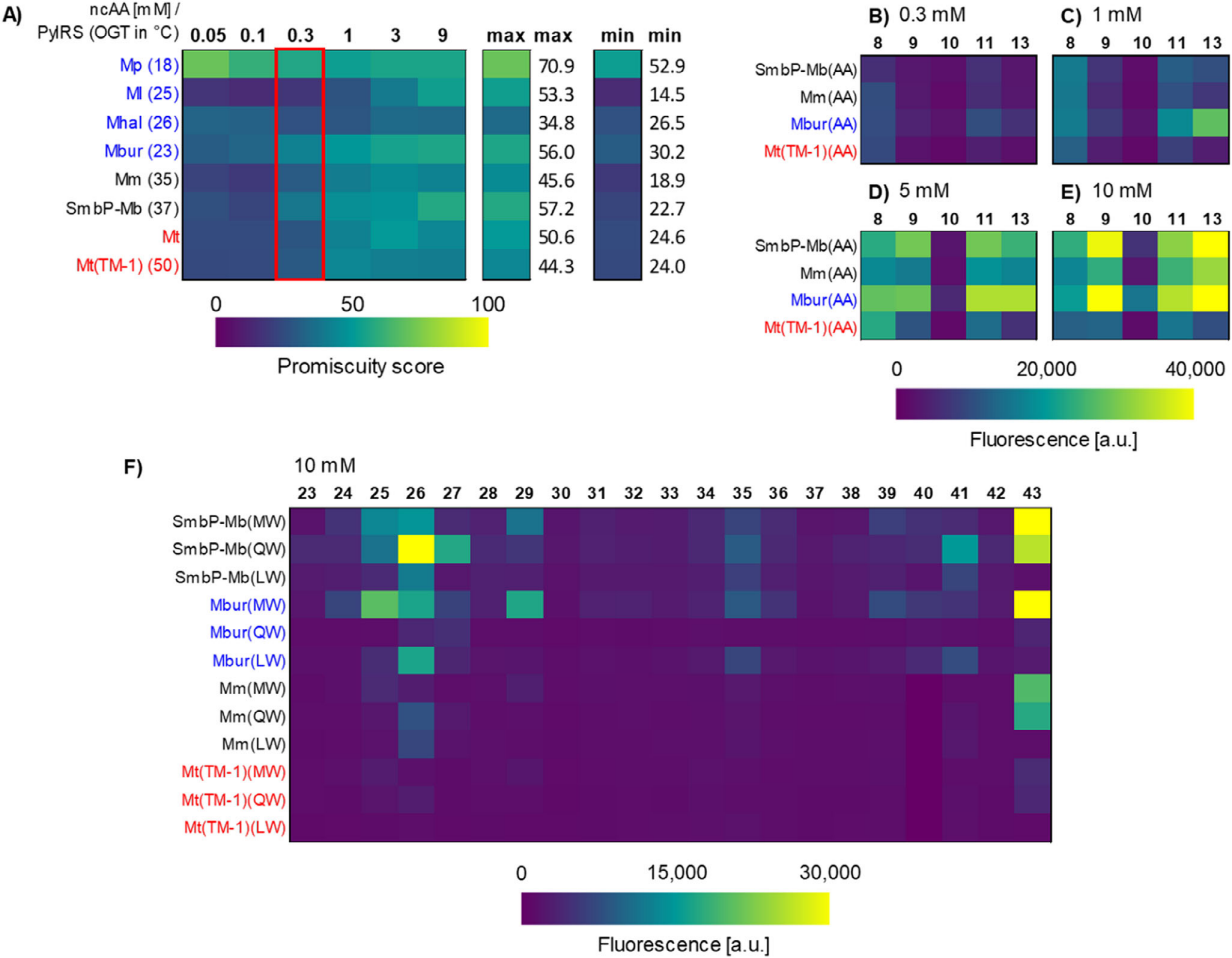
Promiscuity landscape and activity heatmaps of PylRS variants. (A) Heat maps showing the promiscuity score for each PylRS construct at the indicated ncAA concentrations. Maximum (max) and minimum (min) promiscuity scores are provided in an additional column. The optimal growth temperature (OGT) of each PylRS construct is shown in brackets. The ncAA 0.3 mM condition is highlighted (see main text for explanation). (B–E) Heatmaps based on fluorescence of sfGFP(1x amber) expression in *E. coli* BL21(DE3) at ncAA concentrations 0.3, 1, 5, and 10 mM. (F) Selected PylRS mutants tested with 10 mM ncAA.Figures showing the number of replicates and error bars are provided in Figures S40–S44 for panels (B–E); Figures S45–S53 for panel (F). Letters in brackets indicate the N311 and the C313 mutations (in *M. barkeri* numbering). Substrate numbering corresponds to Figure 1. Growth for the Mm and Mt(TM‐1) constructs with substrate **35** was extremely low; these values were therefore excluded. Background suppression was construct‐dependent and ranged from 1100 to 2500 a.u.

Our fluorescence heatmaps (Figure 2) indicated that the Mbur variant may also exhibit higher promiscuity, enabling more efficient incorporation of the tested ncAAs, compared to other variants. Since all PylRS variants display some degree of promiscuity, we sought to quantify this by deriving corresponding promiscuity scores. A standard method to determine enzyme promiscuity is to compare in vitro parameters for new substrates with those of the native substrate [47]. However, since OTSs are complex systems were in vitro parameters might not clearly reflect in vivo efficiency [10], we adapted the concept to generate cell‐based promiscuity scores (Figure 5A).

In this approach, incorporation efficiencies for all substrates (**1**–**5**) were normalized to the best‐performing substrate for each ncAA concentration and mapped onto a scale from 0 to 100 to yield the substrate promiscuity scores (see Supplementary Information S1: Section 2.9 for equations). A theoretically fully promiscuous enzyme would score 100, indicating uniform incorporation efficiency across all substrates. Conversely, a perfectly specific enzyme recognizing only one substrate would have the lowest possible score of 0. Among the functional constructs, the lowest promiscuity score in Figure 5A was 14.5, observed for Ml at 0.1 mM, while the highest score was 57.2 for SmbP‐Mb, followed closely by 56 for Mbur.

Data on intracellular ncAA concentrations are generally limited. However, substrates **2** and **3** show over 90% cell uptake from the medium, for concentrations up to 10 mM [48]. Given that substrate **2** is highly hydrophobic and substrate **3** very hydrophilic, this provides a useful estimate for the uptake efficiency of the remaining ncAA set. These findings suggest that ncAA uptake is generally not a limiting factor for the Pyl analogs used in this study.

Mp showed the highest promiscuity score at low ncAA concentrations but was unfortunately barely functional (Figure 2). No clear trends were observed between promiscuity and the thermal origin of the different PylRS variants. As expected, the promiscuity score increased with rising ncAA concentrations (Figure 5A). This is logical, as both good and bad substrates reach their activity plateaus at different increasing concentrations, as reflected in our data (Figures 2 and 3). Therefore, a high promiscuity score at low ncAA concentrations indicates higher general promiscuity, as the activity plateau has not yet been reached. Notably, Mbur and SmbP‐Mb exhibited the highest promiscuity scores at low ncAA concentrations (0.3 mM, Figure 5A, red box). These results indicate that Mbur combines high in vivo efficiency with broad substrate tolerance, particularly under substrate‐limited conditions.

To evaluate the predictive power of the promiscuity score, we selected the two OTS variants with the highest promiscuity score (Mbur and SmbP‐Mb) at 0.3 mM ncAA concentration and compared their performance to lower‐scoring variants (Mm and Mt(TM‐1)). We used reported mutations for recognizing Phe and Tyr analogs, as well as small aliphatic ncAAs, and transplanted them into the chosen PylRS variants.

The mutation pair N346A:C348A (designated AA), originally described for Mm, enables recognition of Phe and Tyr analogs (**7**‐**22**), [49] Similarly, the mutations responsible for recognizing small aliphatic ncAAs (**23**–**43**) included N311M:N313W, N311Q:N313W, N311L:N313W referred to as MW, QW, and LW, respectively, and were reported for Smbp‐Mb^50^. Additionally, we tested special‐case variants for S‐allyl‐cysteine (**43**) and S‐propargyl‐cysteine (**40**), which originated from SmbP‐Mb and carried the mutations C313W:W382T [50, 51].

Following an initial pre‐screen of the Phe and Tyr analogs (Figure S16), the ncAAs substrates with good activity were further analyzed in a concentration‐dependent manner to gain deeper insights (Figure 5B–E). In these tests, Mbur and SmbP‐Mb demonstrated the best performance, with Mbur being more effective at lower ncAA concentrations, aligning well with the derived promiscuity scores. Notably, Mbur successfully incorporated **10**, which none of the other PylRS were able to incorporate.

Given that psychrophilic enzymes, including Mbur, often contain a higher proportion of amino acids with polar or charged side chains, we hypothesized that this might provide an advantage in recognizing ncAAs with similar properties. To test this, we evaluated substrates **18**–**22** using the AA variants shown in Figure 5B–E. Mbur was able to incorporate ncAAs **18**–**21**, with Sulfotyrosine (**18**) being incorporated most effectively. In contrast, SmbP‐Mb produces weaker signals for substrates **18** and **19,** while Mm and Mt(TM‐1) failed to incorporate any of these substrates (Figure S17).

Since these analogs are most likely synthesized by Tyr derivatization, the purchased ncAAs likely contained some degree of Tyr contamination (up to 5% for substrates 18–22). Therefore, we tested Tyr at various concentrations as a control. Assuming a maximum of 0.5 mM Tyr contamination at a 10 mM substrate concentration, we supplied up to 1.5 mM Tyr to the cultures. However, no Tyr incorporation was detected. For substrates **40** and **43**, Mbur outperforms the other variants at lower ncAA concentrations and with multiple stop codons, even reaching wild‐type levels of sfGFP production (Figures S18 and S21).

Overall, more ncAAs served as substrates for Mbur and SmbP‐Mb than for Mm and Mt(TM‐1) (Figure 5F), supporting the predictive value of the derived promiscuity scores. These findings suggest that the promiscuity scoring may serve as a practical guide for selecting scaffolds in future OTS engineering projects, consistent with analogous observations in other enzyme systems [52, 53].

### 2.5 Validation of Small‐Scale Fluorescent Assays by Scaled‐Up Protein Production and Purification

**FIGURE 6:**
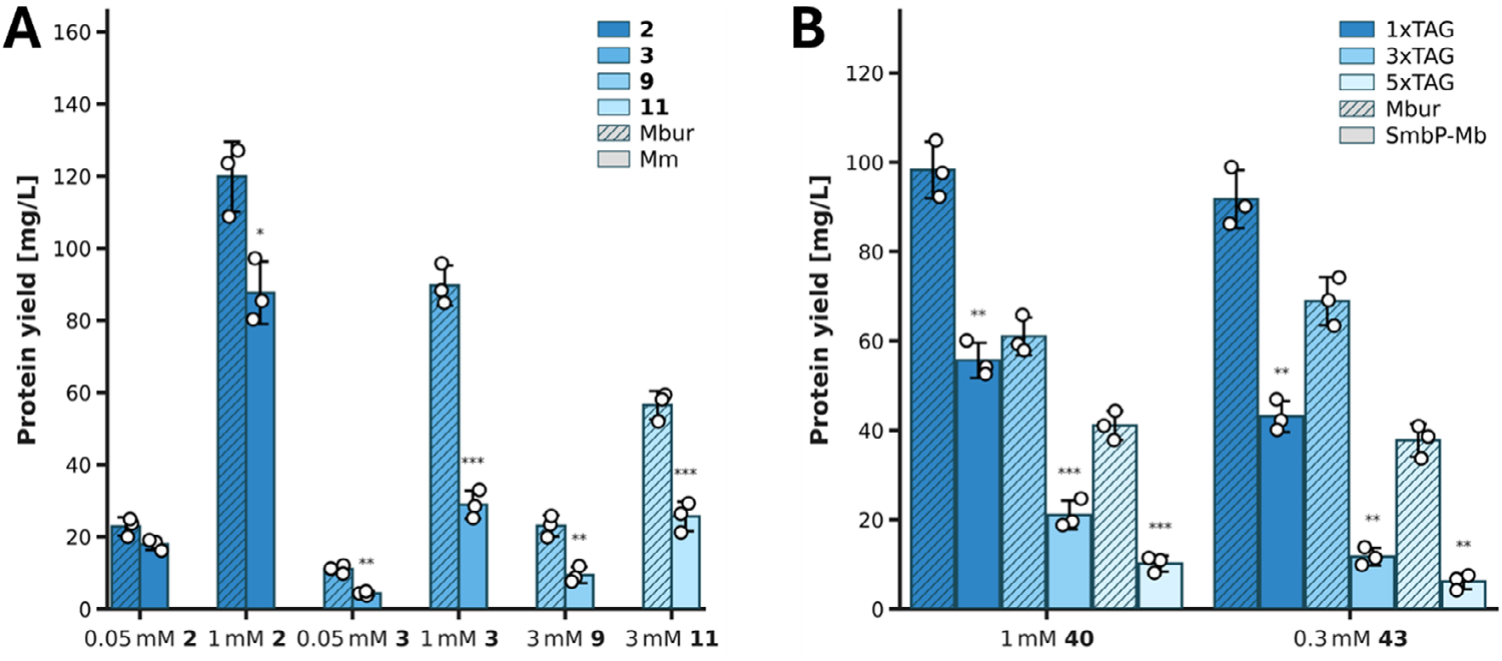
Protein yields of sfGFP from small‐scale shake flask cultures. Yields are reported as milligrams of purified protein per liter of culture. (A) *E. coli* BL21(DE3) expressing sfGFP(1x TAG) as reporter construct. (B) *E. coli* B‐95.ΔA expressing the reporter constructs indicated in the panel legend. Substrate concentrations are specified in the legend, with numbering corresponding to Figure 1. Statistical comparisons of all constructs versus Mbur (striped) were performed using unpaired two‐sample *t*‐tests (Welch's *t*‐test, *n* = 3 biological replicates). Adjusted *p*‐values are indicated as follows: *p < 0.05, **p < 0.01, ***p < 0.001. Detailed information on the PylRS variants and ESI‐MS analysis of purified proteins is provided in Tables S1 and S2.

Protein yields obtained from scaled‐up production and purification (Figure 6) closely mirrored the trends observed in the small‐scale fluorescence assays, supporting the reliability of the high‐throughput screening approach. One notable exception was substrate **40**, for which Mbur produced higher‐than‐anticipated protein yields (Figure S18B). Nevertheless, within each dataset, fluorescence intensity and purified protein yield exhibited strong agreement.

For single‐site ncAA incorporation, yield improvements with Mbur ranged from 25% (for 1 mM substrate **2**) and 200% (for 1 mM substrate **3**) relative to Mm (Figure 6A). The most remarkable improvements were observed in the multi‐site incorporation of five ncAAs (**43** and **40**), with an improvement of over 400% and 300%, respectively (Figure 6B). Notably, the performance gap between SmbP‐Mb and Mbur increased with the number of incorporated ncAAs (Figure 6B), highlighting the cumulative advantage of the Mbur scaffold under increased translational demand.

To assess the predictive robustness of the 96‐well plate assays, we analyzed the small scale 96‐well plate sfGFP fluorescence data in dependence on the isolated protein yields of the shaking flask cultures by linear regression and derived the R^2^ values (see supplements Figure S97). Linear regression analysis revealed a notably strong correlation between the 96‐well plate assays for each ncAA, with R^2^ values consistently exceeding 0.97. Even when combining data from cultivations with different ncAAs, the R^2^ values remained high, reaching at least 0.85, indicating a strong correlation of small‐scale fluorescence and shake flask protein yields.

However, the combined R^2^ values were slightly lower compared to those from a single ncAA. This suggests that protein yields are influenced by factors beyond the specific ncAA concentration, potentially including ncAA toxicity leading to growth impairment. Overall, these results demonstrate the robustness of the data and indicate that protein yields from 96‐well plate assays are reliably translatable to shake flaks cultivations. These findings validate the use of high‐throughput fluorescence screening to identify scalable OTS improvements, with Mbur consistently outperforming established PylRS variants under both single‐ and multi‐site incorporation conditions.

## 3 Discussion

Our study demonstrates that psychrophilic enzymes, exemplified by the Mbur variant, represent a powerful and previously underexplored source of efficient orthogonal translation systems (OTSs). Mbur consistently outperformed established systems like Mm and Alv in both single and multi‐site ncAA incorporations, including under challenging conditions such as low ncAA concentrations and reduced cultivation temperatures. Moreover, Mbur incorporated several non‐Pyl derivatives more efficiently and derivatives that could not be incorporated before (substrate 10, for example) by other PylRSs, showcasing exceptional substrate promiscuity. These findings validate the use of cell‐based promiscuity scores as predictive tools for OTS engineering and introduce psychrophilic aaRSs as promising scaffolds for future genetic code expansion strategies.

While psychrophilic enzymes are often characterized by enhanced catalytic efficiency at low temperatures [54], this is typically accompanied by reduced thermal stability at elevated temperatures [55]. As a consequence, only a subset of psychrophilic scaffolds is expected to retain sufficient structural integrity to function efficiently under standard laboratory conditions in *E. coli*. Consistent with this, only one of the four psychrophilic candidates tested in this study (Mbur) exhibited superior performance.

The observation that only one of four psychrophilic candidates is superior does not contradict the concept but rather reflects the balance between flexibility and stability required for function in a mesophilic host. Increased conformational flexibility can facilitate substrate accommodation and mutational tolerance, but only when supported by sufficient structural robustness. In this context, psychrophilic enzymes can be viewed as a high‐variance reservoir from which rare variants with an optimal balance of flexibility and stability can be identified. Mbur PylRS represents such a case.

We initiated this work to address the persistent limitation of low in vivo efficiency in PylRS‐based systems. To this end, we systematically compared eight +N PylRS homologs from different organisms and included 15 ΔN class variants, resulting in a total set of 23 OTSs. Among these, we identified a highly active variant from the psychrophilic (cold‐active) organism *Methanococcoides burtonii* (Mbur). Its superior performance was particularly evident at reduced temperatures and low ncAA concentrations (Figures 3 and 4), suggesting intrinsic catalytic advantages rather than mere expression effects.

The enhanced activity of Mbur cannot be attributed to elevated intracellular expression levels alone, as normalization of in vivo performance to enzyme abundance (Figure S10, Figure 2) confirms intrinsic functional differences between variants. While direct structural measurements of flexibility were beyond the scope of this study, the observed performance profile is consistent with established characteristics of psychrophilic enzymes, which typically exhibit increased conformational plasticity and reduced activation energy barriers at lower temperatures [19, 54, 55].

The ability of Mbur to maintain in vivo efficiency at reduced temperatures and low ncAA concentrations, therefore aligns with known principles of cold adaptation, rather than representing an isolated performance effect. We emphasize that our data support a functional correlation with psychrophilic origin, while acknowledging that detailed structural and thermodynamic analyses will be required to directly quantify conformational flexibility–activity relationships.

The superior incorporation efficiency was consistently observed across diverse substrate classes, demonstrating robustness rather than substrate‐specific bias. Particularly remarkable was the multi‐site ncAA incorporation with BocK (**2**) and especially S‐allyl‐cysteine (**43**). To the best of our knowledge, no previous PylRS mutant has achieved such high ncAA incorporation efficiency for non‐Pyl‐analogs, approaching that of the wild‐type reference. This is practically significant, as improved efficiency at lower ncAA concentrations reduces experimental cost and expands feasibility for multi‐site modification strategies.

Currently, only two OTSs—*Mj*TyrRS [56, 57, 58] and AfTyrRS [57] – are known to approach wild‐type recombinant protein production yields. The identification of Mbur as a third high‐performance OTS scaffold expands the toolbox for genetic code expansion. Importantly, additional high‐efficiency OTS platforms increase the potential for multiplexed ncAA incorporation using liberated codons [59, 60]. When the Mm was used in a recoded *E. coli* strain, OTS efficiency is higher for some of these newly created codons, but only to a limited extent [60]. This result suggests that the in vivo efficiency of the PylRS OTS remains a bottleneck, even when free codons are available. In contrast, Mbur, maintained high performance even in an RF1‐deficient strain (Figures 3, 4, and 6) suggesting improved scalability in recoded organisms.

The high performance of Mbur, even at very low ncAA concentrations, opens new possibilities for coupling with ncAA‐producing metabolic pathways, potentially eliminating the need for external ncAA supply, as we recently elaborated [2]. Efficient incorporation below 1 mM intracellular concentration is critical, as canonical amino acid pools are typically maintained within this range, and engineering metabolic pathways to achieve substantially higher ncAA concentrations remains challenging [61].

This high efficiency at low ncAA concentration also has further implications. The low cost and minimal concentration required for the efficient incorporation of **43** allow recombinant proteins containing one or even multiple bioorthogonal alkene functions to be produced at wild‐type levels with negligible additional cost. The alkene function can be addressed with the inverse electron demand Diels–Alder (IEDDA) reaction, using a tetrazine moiety [62, 63]. More broadly, 17 of the 43 incorporated ncAAs provide reactive handles for bioorthogonal chemistry (Figure 1), underscoring the translational relevance of enhanced OTS efficiency.

Promiscuity is a central property in enzyme engineering, as it lowers the mutational barrier for evolving new substrate specificity [52, 53]. Our results demonstrate that Mbur exhibits elevated substrate breadth relative to other PylRS variants. Importantly, mutations conferring recognition of diverse ncAAs were directly transplanted from previously engineered PylRS variants without further optimization. Despite this, Mbur not only retained functionality but also frequently outperformed the donor scaffolds. Such behavior is uncommon in aaRS engineering, where transferred mutations typically require additional stabilization or compensation [15, 64, 65, 66, 67, 68]. This observation strongly supports the intrinsic mutational tolerance and adaptability of the Mbur scaffold.

Collectively, these Findings Position Mbur as a Highly Promising Template for Expanding the ncAA Repertoire and Advancing Synthetic Biology Applications

### 3.1 Outlook

Our findings suggest that psychrophilic organisms could represent a promising reservoir for identifying efficient OTS scaffolds. Psychrophilic enzymes, characterized by their flexible architecture, maintain functionality at low temperatures due to structural adaptations such as reduced hydrophobic core packing, elongated loops, fewer hydrogen bonds, and less dense salt bridges [69]. These features are frequently associated with increased conformational plasticity and substrate breadth [70].

Although relatively few psychrophilic enzymes have been extensively studied [70], viral proteins exhibit similar features, such as elongated loops and less densely packed cores [71]. Tokuriki et al. developed a mutational gradient robustness model for viral proteins, demonstrating lower fitness costs per mutation compared to thermophilic proteins, thereby enhancing mutational tolerance and adaptability [71, 72]. Such properties likely contribute to the enhanced mutational tolerance observed for Mbur. Psychrophilic enzymes therefore, provide a compelling framework for exploring evolutionary innovation and engineering flexibility.

From a biocatalysis perspective, cold‐active enzymes often exhibit higher Km values, lower activation free energy (ΔG‡), and greater entropic contributions (TΔS‡), enabling efficient turnover at reduced temperatures [23, 24, 73]. Although detailed kinetic analysis of Mbur remains to be conducted, the observed performance patterns align with established principles of psychrophilic enzyme behavior.

Historically, orthogonal translation research has largely focused on mesophilic and thermophilic aaRS scaffolds. Our results demonstrate that psychrophilic enzymes constitute a valuable and underutilized alternative. These advantages may extend beyond aaRSs to other translational components, including orthogonal ribosomes and elongation factors. Leveraging the inherent flexibility and mutational tolerance of psychrophilic macromolecules may open new directions in genetic code expansion and synthetic biology.

In our study, we identified a psychrophilic PylRS enzyme combining high in vivo efficiency with broad substrate tolerance and strong multi‐site incorporation capacity. These properties are highly desirable for protein engineering and synthetic biology yet remain largely unexplored in psychrophilic systems. We anticipate that broader exploration of cold‐adapted enzymes will reveal additional scaffolds with comparable or superior properties, accelerating the practical implementation of expanded genetic code.

### 3.2 Materials and Methods

#### 3.2.1 Canonical and Non‐Canonical Amino Acids

Canonical amino acids were purchased from Carl Roth. Non‐canonical amino acids were obtained from Fluorochem, Iris Biotech, Chempur, Sigma‐Aldrich (Merck), Chiralix, Toronto Research Chemicals, Carl Roth, ThermoFisher Scientific, and TCI Deutschland (see Table S4).

### 3.3 Plasmid Vector Construction, PylRS and Reporter Sequences

The pTECH vector was a gift from Dieter Söll (Addgene plasmid #104073) [11]. Genes were ordered as codon‐optimized fragments from Twist Bioscience. All plasmids were assembled using Golden Gate cloning and confirmed by Sanger sequencing. Point mutations were introduced by non‐overlapping inverse PCR. All PylRS and reporter sequences used in this study are provided in the supplementary materials.

### 3.4 Analysis of SUMO‐sfGFP Expression by Intact Cell Fluorescence

*E. coli* BL21(DE) cells were used for small‐scale expression of reporter constructs. Electrocompetent cells were transformed with the orthogonal translation system and reporter plasmids. LB agar plates for plating contained 1% glucose and the appropriate antibiotics.

Single colonies were used to inoculate 2 mL of LB medium (in 14 mL tubes) supplemented with 1% glucose and appropriate antibiotics, and cultures were grown overnight to saturation. Assays were performed in 96‐well plate format. Cultures were diluted 1:100 into ZYP‐5052 auto‐induction medium to a final volume of 100 µL per well, supplemented with antibiotics and ncAAs.

Cells were grown in black µ‐plates (Greiner Bio‐One, Kremsmünster, Austria) sealed with a gas‐permeable membrane (Breathe‐Easy, Diversified Biotech, Dedham, MA, USA) and incubated with orbital shaking for 24 h at 37°C.

For endpoint measurements (Tecan M200, Männedorf, Switzerland), the sealing membrane was removed, and fluorescence was measured using a gain setting of 85. For OD_600_ measurements, 50 µL of ZYP‐5052 medium was added to clear 96‐well µ‐plates, followed by 50 µL of culture. Fluorescence excitation and emission wavelengths were set to 481 and 511 nm, respectively. Fluorescence values were normalized to the corresponding OD_600_ values.

### 3.5 Temperature Dependent In Vivo Performance and Flow Cytometry

Cells were transformed with the orthogonal translation system, reporter plasmids, and necessary controls. LB agar plates used for plating contained 1 % glucose and corresponding antibiotics. Single colonies of clones were inoculated into 2 mL LB (in 13 mL tubes) supplemented with 1 % glucose and the appropriate antibiotics and grown to saturation overnight. Subsequently, 4 mL ZYP‐5052 medium, supplied with antibiotics and the desired ncAA, was inoculated (1:50) and grown to an OD_600_ = 0.4–0.6 (∼5 h). The culture was then split into 2 × 2 mL aliquots: one was incubated at 18°C and the other one at 37°C. After 24 h, cells were diluted to an OD_600_ of ∼0.025 using sterile filtered PBS (0.8 µL of the culture in 500 µL PBS) and analyzed by flow cytometry. For cultures incubated at 18°C, this procedure was repeated at additional timepoints of 48 and 72 h

Cytometric analyses were conducted using an Attune NxT flow cytometer (ThermoFisher Scientific, Waltham, MA, USA) equipped with 488 and 561 nm lasers. The fraction of cells containing the desired and reporter proteins was determined using a gating strategy targeting single cells while excluding aggregates and other background noise.

For the cultures grown at 18°C, the maximum value observed within the 24–72 h interval was used for Figure 4.

### 3.6 Determination of Cytosolic Expression of PylRS Variants via Split‐GFP Assays

Cells were transformed with the orthogonal translation system encoding the N‐terminal sfGFP(11)‐tagged PylRS and the reporter plasmid pET28a_sfGFP(1–10). LB agar plates used for selection contained 1% glucose and the appropriate antibiotics.

Single colonies were used to inoculate 100 µL of LB medium supplemented with 1% glucose and appropriate antibiotics in transparent 96‐well plates (Greiner Bio‐One, Kremsmünster, Austria). Plates were sealed with a gas‐permeable membrane (Breathe‐Easy, Diversified Biotech, Dedham, MA, USA) and incubated for 24 h at 37°C.

For expression assays, cultures were diluted 1:100 into ZYP‐5052 auto‐induction medium to a final volume of 100 µL per well, supplemented with antibiotics. Cells were grown in transparent 96‐well plates sealed with a gas‐permeable membrane and incubated with orbital shaking (300 rpm) for 24 h at 37°C.

For endpoint measurements, 1 µL of culture was diluted into 250 µL of PBS in 96‐well plates and analyzed using an Attune NxT flow cytometer (Thermo Fisher Scientific, Waltham, MA, USA).

### 3.7 Protein Expression and Purification

For expression of SUMO‐sfGFP variants, *E. coli* strains were cultured in 10 mL of ZYP‐5052 medium supplemented with the indicated ncAA concentrations and appropriate antibiotics. The expression cultures were inoculated from starter cultures at a 1:100 dilution. Shake flasks were incubated for 24 h at 37°C with shaking at 200 rpm. Cells were harvested by centrifugation and either stored at –80°C or processed immediately for protein purification.

Cell pellets were resuspended in lysis buffer (50 mM sodium phosphate, 300 mM NaCl, 20 mM imidazole, pH 8.0) and lysed using B‐PER Bacterial Protein Extraction Reagent (Thermo Fisher Scientific, Waltham, MA, USA) according to the manufacturer's instructions, supplemented with phenylmethanesulfonyl fluoride (PMSF; 1 mM final concentration), DNase, and RNase. Cleared lysates were loaded onto an equilibrated Ni‐NTA column and purified either using a P‐1 peristaltic pump (Pharmacia Biotech, now Cytiva, Marlborough, MA, USA) or by gravity flow.

After washing with 10 column volumes of lysis buffer, bound proteins were eluted with elution buffer (50 mM sodium phosphate, 300 mM NaCl, 500 mM imidazole, pH 8.0). The first 1.5 mL (1 mL for gravity flow), corresponding to the void volume, was discarded. The subsequent 1 mL eluate was collected and dialyzed using cellulose tubing against 1 L of dialysis buffer (50 mM sodium phosphate, 300 mM NaCl, pH 8.0) for at least 2 h with three buffer changes.

Protein concentrations were determined by measuring the absorbance of the sfGFP chromophore at 488 nm.

### 3.8 Intact Protein ESI‐MS

Intact protein mass measurements of purified SUMO‐sfGFP variants were performed by electrospray LC‐MS using a Waters H‐Class instrument equipped with a Waters Acquity UPLC Protein BEH C4 column (300 Å, 1.7 µm, 2.1 mm × 50 mm). Gradient elution was carried out from 5% MeCN in aqueous 0.01% formic acid to 95% MeCN over 6 min at a flow rate of 0.3 mL/min. Mass analysis was performed using a Waters Xevo G2‐XS QTof mass spectrometer. Proteins were ionized in positive ion mode with a cone voltage of 40 V. Raw data were processed using the maximum entropy deconvolution algorithm. Processed data were exported and plotted using QtiPlot (version 0.9.9.7).

## Conflicts of Interest

The authors declare no conflicts of interest.

## Supporting information




**Supporting File**: advs75333‐sup‐0001‐SuppMat.docx.

## Data Availability

The data that support the findings of this study are available in the supplementary material of this article.
