# Supplementary material for: Cold Orthogonal Translation: A Psychrophilic Pyrrolysyl‐tRNA Synthetase Boosts Genetic Code Expansion in E. coli
Source: Adv Sci (Weinh). 2026 Apr 15;13(38):e13600. doi: 10.1002/advs.202513600 (PMC13335545; doi:10.1002/advs.202513600)
Supplement: Supplementary file 1 — Supporting File: advs75333‐sup‐0001‐SuppMat.docx. [file ADVS-13-e13600-s001.docx]

Revised Manuscript advs. 202513600R1

Supplementary Information

**Cold Orthogonal Translation: A Psychrophilic Pyrrolysyl-tRNA Synthetase Boosts Genetic Code Expansion in *E. coli***

Nikolaj G. Koch^1,2,6,7^, Peter Goettig^3^, Michael A. Nash^6,7^, Juri Rappsilber^1,5^*,
Nediljko Budisa^2,4^*

^1^ Bioanalytics Group, Institute of Biotechnology, Technische Universität Berlin, 10623 Berlin, Germany

^2^ Biocatalysis Group, Institute of Chemistry, Technische Universität Berlin, 10623 Berlin, Germany

^3^ Department of Pharmaceutical and Medicinal Chemistry, Institute of Pharmacy, Paracelsus Medical University, 5020 Salzburg, Austria

^4^ Chemical Synthetic Biology Group, Department of Chemistry, University of Manitoba, Winnipeg, MB R3T 2N2, Canada

^5^Wellcome Centre for Cell Biology, University of Edinburgh, Edinburgh EH9 3BF, United Kingdom.

^6^Department of Chemistry, Institute of Physical Chemistry, University of Basel, 4058 Basel, Switzerland

^7^Department of Biosystems Science and Engineering, ETH Zurich, 4056 Basel, Switzerland

*Correspondence to: Juri.Rappsilber@tu-berlin.de or Nediljko.Budisa@umanitoba.ca

[1. Supplementary Results 4](#_Toc226384622)

[1.1 Phylogenetic PylRS Analysis 4](#_Toc226384623)

[1.2 Determination of the PylRS Variable Regions (Linker) 4](#_Toc226384624)

[1.3 Analyses of the Amino Acid composition of PylRS 5](#_Toc226384625)

[1.3.1 The special case of the PylRS linker of *Methanosarcina* 6](#_Toc226384626)

[1.4 Structural Biology Considerations on PylRS 8](#_Toc226384627)

[1.5 Complete heatmaps for +N and ΔN PylRS variants 9](#_Toc226384628)

[1.6 Cytosolic expression of PylRS variants determined by split-GFP assays 12](#_Toc226384629)

[1.7 Inducible Promotor Validation 14](#_Toc226384630)

[1.8 Comparison of pUltra and pTECH OTS setups 15](#_Toc226384631)

[1.9 Multi-Site ncAA incorporation by +N and ΔN PylRS variants 17](#_Toc226384632)

[1.10 Temperature-dependent expression of wild-type sfGFP and background suppression 21](#_Toc226384633)

[1.11 Full Heatmaps for double Alanine and Glycine PylRS constructs 22](#_Toc226384634)

[1.12 Additional data for promiscuity analysis 23](#_Toc226384635)

[1.13 Testing PylRS constructs for SproC (40) and Sac (43) incorporation 24](#_Toc226384636)

[1.14 Multi-Site ncAAs incorporation in BL21(DE3) 25](#_Toc226384637)

[1.15 Multi-Site Incorporation of Sac (43) and SproC (40) 26](#_Toc226384638)

[1.16 Comparison of *Mj*TyrRS and *Mbur*PylRS performance 29](#_Toc226384639)

[2. Supplementary Data, DNA sequences and mass-profiles of intact ncAA-containing proteins 31](#_Toc226384640)

[2.1 Fluorescence Assays on the influence of ncAA concentration on PylRS-based OTS 31](#_Toc226384641)

[2.2 DNA/RNA Sequences 53](#_Toc226384642)

[2.2.1 Used PylRS 53](#_Toc226384643)

[2.2.2 Reporter Constructs 53](#_Toc226384644)

[2.3 tRNA Sequences and secondary structure prediction 55](#_Toc226384645)

[2.4 Deconvoluted ESI-MS spectra of intact ncAA-containing protein variants 56](#_Toc226384646)

[2.5 ESI-MS data 70](#_Toc226384647)

[2.6 Protein yields 72](#_Toc226384648)

[2.7 Correlation between small-scale fluorescence measurements and shake- flask sfGFP yields 74](#_Toc226384649)

[2.8 Flow Cytometry Dot plots 75](#_Toc226384650)

[2.9 Equations used to calculate the Promiscuity Score 80](#_Toc226384651)

[2.10 OGT References and Source Organisms for +N PylRS Variants Used in Experimental and *In Silico* Analyses 81](#_Toc226384652)

[3. Materials 82](#_Toc226384653)

[3.1 Non-Canonical Amino Acids 82](#_Toc226384654)

[3.2 Oligonucleotides 84](#_Toc226384655)

[4. References 84](#_Toc226384656)

# Supplementary Results

## Phylogenetic PylRS Analysis

Initial sequencing discrepancy: Phylogenetic analysis was performed for all *Methanosarcinales* and *Methanomassiliicoccales* PylRS sequences (**Figure 1**). Sequences were retrieved from NCBI genome databases; for *M. alaskense* from the JGI database. In addition, the sequence of *M. thermophila* was included, which had previously been determined with degenerate primer amplification from genomic DNA prior to publication of the complete genome.^1^

Unexpectedly, the sequence did not match the PylRS sequence extracted from the subsequently published genome of *M. thermophila* (strain TM-1). Because two additional genome-derived sequences (CHTI 55 and MT-1) match the TM 1 sequence, we consider TM-1 to represent the correct reference and refer to it here as *Mt*(TM 1). The alternative *Mt*PylRS variant shows higher sequence similarity to the only known psychrophilic *Methanosarcinae* PylRS from *M. lacustris* and was therefore retained for further analysis.

Selection criteria for psychrophilic candidates: As shown in the phylogenetic tree (**Figure 1A**), six potential psychrophilic candidates were identified. At least one representative was selected from each genus. When multiple variants were available, the sequence from the species with the lowest optimal growth temperature (OGT) was chosen.

Choice of PylRS variants from *Methanococcoides burtonii* and *alaskense*: At the time of project planning (December 2019), only the genome of *M. burtonii* was available and was therefore selected. In retrospect, this choice remains justified, as both variants share >95% sequence identity.

Notably, analysis of the corresponding tRNAs revealed substantial differences in predicted folding free energy (**Figure S67**). The calculated free energy of *M. burtonii* tRNA^Pyl^ is ~ 6 kcal/mol higher at 37 °C, suggesting enhanced adaptation to lower temperatures. For this reason, *M. burtonii* would have been selected even if both genomes had been available initially.

## Determination of the PylRS Variable Regions (Linker)

ColabFold^2^ was used to predict one representative PylRS structure from each family. In addition to structural models, this approach provides residue-specific confidence scores (pLDDT). According to the original AlphaFold2 framework, regions with low pLDDT scores reliably correspond to structurally disordered segments.^3,4^

Linker regions between the N- and C-terminal domains were defined based on consistently low-confidence regions. Predicted linker boundaries were cross-validated against family-wide sequence alignments. Where predicted start or end residues were not conserved within a family, additional structure predictions were performed to refine boundary assignment.

The C-terminal linker boundary was typically defined by a conserved AA sequence. Minor variability at the N-terminal start occasionally resultrd in shifts of one residue . The predicted AlphaFold2 structures are shown in Figures S3 and S4, with corresponding PDB IDs.

## Analyses of the Amino Acid composition of PylRS

Clustering and arrangement into a heatmap: To analyze the AA composition, PylRS sequences from  *Methanosarcina,* *Methanococcoides*, *Methanohalophilus* and *Methanolobus* were separated into catalytic core (linker removed) and linker region. Both segments were clustered and visualized as heat maps (**Figure S1**). Heatmaps display amino acid percentages, grouped according to physicochemical properties, as well as total enzyme and linker length.

Comparison of AA usage: Across genera, no pronounced trends in amino acid usage were observed for the catalytic core.. For example, the proportion of nonpolar residues varies by less than 2.5%, and enzyme lengths differ by no more than three residues when the linker region is excluded. The analysis indicates that while there are no discernible patterns in AA usage among the PylRS enzymes studied, their lengths remain fairly consistent, at least when the variable linker region is excluded (vide infra).

Consistency in enzyme length: In contrast, linker regions exhibit greater heterogeneity. The most abundant residues within linkers align with known enrichment patterns of intrinsically disordered proteins (IDPs), particularly P, E, S, Q, K, A, and G (**Figure S1**).^5^ A notable difference is lysine content: *Methanococcoides* and *Methanohalophilus* linkers contain substantially more lysine than the other genera. A higher K content could promote or fine tune linker-tRNA interactions because lysine is positively charged at physiological pH and RNAs are negatively charged molecules.^6^ In addition, the linker of *Methanosarcina* contains much less acidic and basic AAs than the others. Despite compositional differences, calculated intrinsic disorder scores (according to Theillet et al.)^5^ are comparable across genera, suggesting functional conservation of linker flexibility.

Understanding the substantial differences in linker composition is challenging in the absence of structural data; however, all available evidence strongly indicates that this region is pivotal in fine-tuning enzymatic activity to suit the specific environmental conditions of the respective organisms. These tuning effects may extend to influencing the interaction between the respective PylRS and its corresponding tRNA^Pyl^, as variations are observed at certain positions depending on the organism's origin within the family.^6^ Previous studies demonstrate that altering the linker length can have effects on wild-type and engineered PylRS variants, although the precise mechanisms underlying this phenomenon remain unclear.^7^


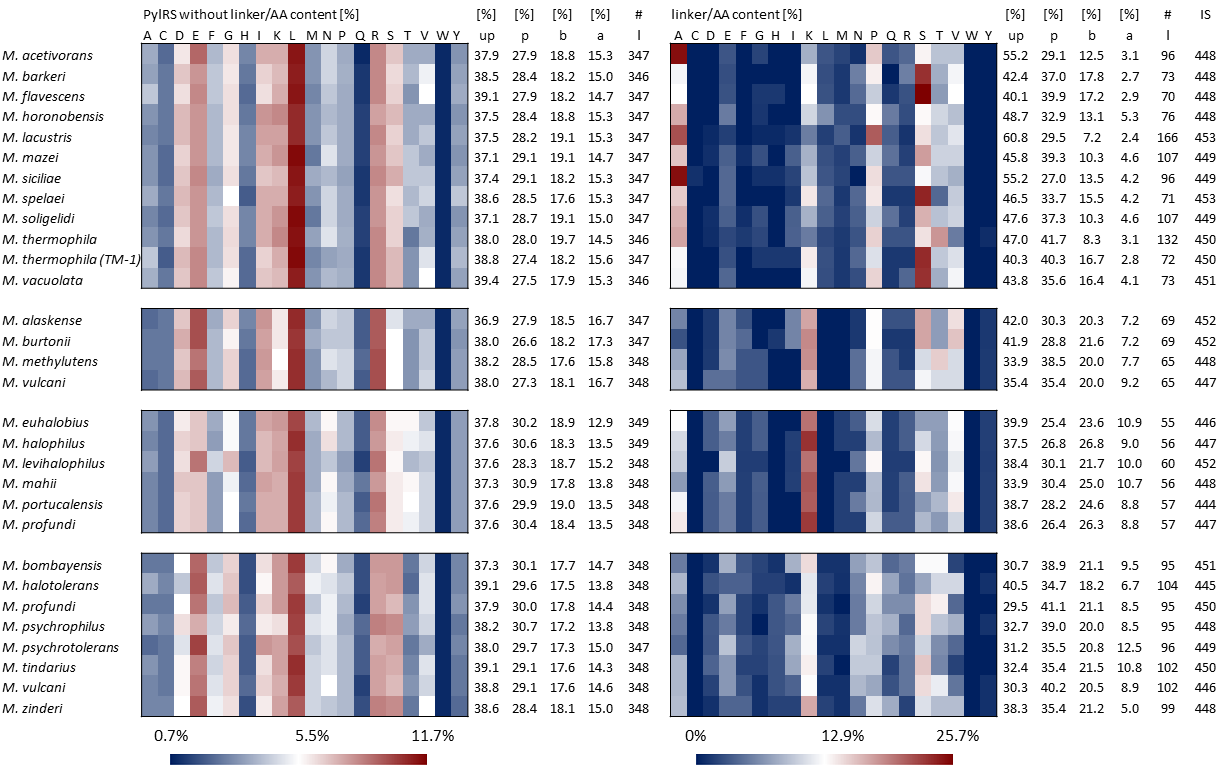


**Figure S1.** Amino acid composition of the PylRS enzymes without linker (left) and with linker (right). The first group is *Methanosarcina*, the second *Methanococcoides*, the third *Methanohalophilus* and the fourth *Methanolobus* (From top to bottom). Abbreviations are up = unpolar, p = polar, b = basic and a = acidic amino acids, in per cent; l = length of the enzyme/linker in number of amino acids; IS = IDP score.

### The special case of the PylRS linker of *Methanosarcina*

Removal of the linker region reveals that catalytic core lengths are nearly identical across PylRS enzymes, differing by at most one residue (**Figure S1**). In contrast, linker lengths within *Methanosarcina* exhibit exceptional variability, ranging from 70 to 166 amino acids. This degree of variation is not observed in other genera of the same family.

Based on this observation, we hypothesized a potential correlation between the thermal origin and the linker length of *Methanosarcina* PylRS enzymes. To examine a potential relationship between thermal adaptation and linker length, a sequence identity matrix was constructed based on the PylRS sequences (**Figure S2**). An alignment of the full-length enzyme was conducted, excluding the linker. This exclusion was warranted as it allowed for the establishment of a relationship between the remainder of the enzyme and the linker length. It is worth noting that significant sequence gaps were considered acceptable for a robust relationship estimation only under certain circumstances.^8^

If trends in temperature adaptation were present, they would likely manifest within the enzyme sequence and exhibit some degree of clustering. Based on these findings, we propose that linker length in PylRS enzymes predominantly influences catalytic activity tuning rather than the enzyme sequence itself, although the sequence's proline content may contribute to rigidity.


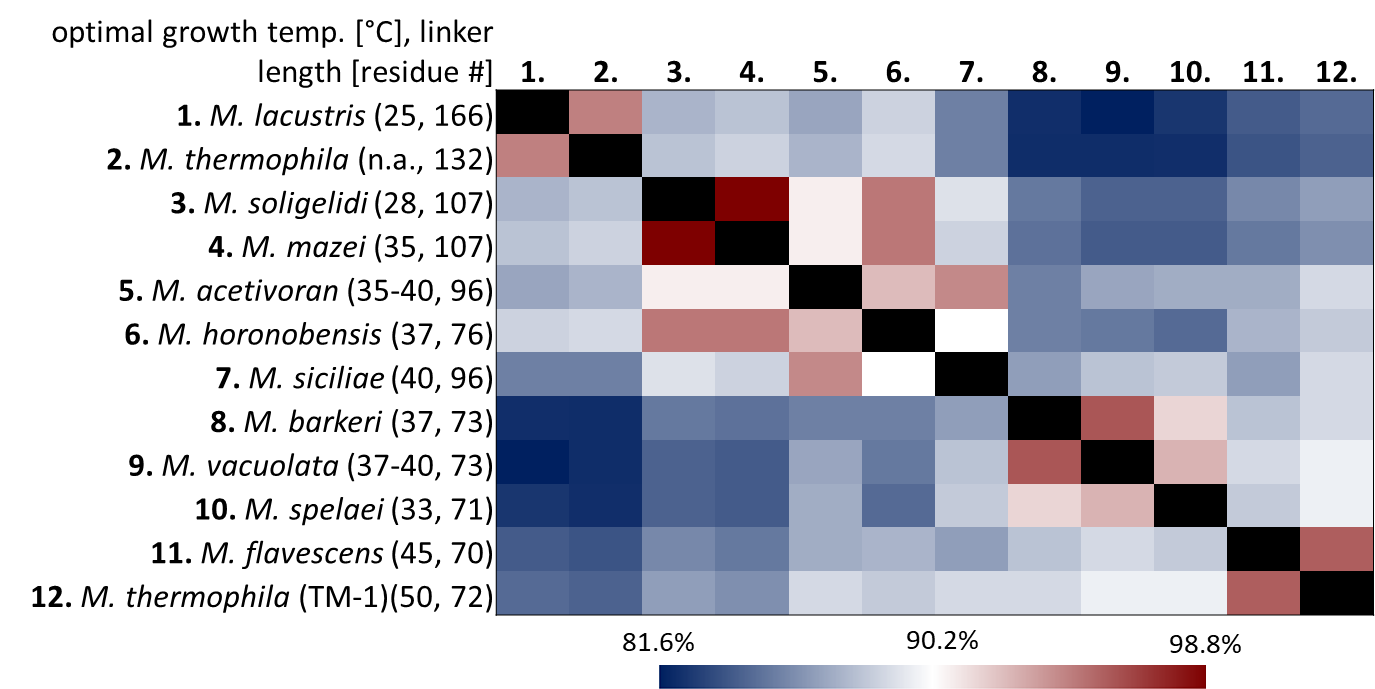


**Figure S2.** Sequence identity matrix of the *Methanosarcina* PylRSs without linker. [AA] = number of amino acids as linker, n.a. = not available.

Sequence clustering mirrors the phylogenetic tree the phylogenic tree (**Figure 1A**). Examination of *Ml*PylRS (**1.**) and *Mt*PylRS(TM‑1) (**12.**) indicates a trend consistent with an inverse relationship between OGT and linker length: the most psychrophilic variants possess the longest linkers. Longer linkers are generally associated with increased conformational flexibility, consistent with adaptation to low-temperature environments. Similar observations have been reported for psychrophilic cellulases, where linker extensions are substantially longer than in mesophilic homologs.^9,10^ In the case of PylRS enzymes, increased proline content accompanies linker elongation. Given the conformational rigidity imposed by proline residues, this enrichment may partially counterbalance excessive flexibility.

Based on sequence identity (>94%) and linker characteristics, the MtPylRS variant analyzed here does not display features typical of thermophilic enzymes. Despite its historical designation, its properties are more consistent with psychrophilic adaptation.

## Structural Biology Considerations on PylRS

The predicted structures of *M. mazei* (magenta) and *M. burtonii* (red) were compared with the experimental structure of the C-terminus of *M. mazei* (PDB ID: 2Q7H, blue)^11^ (**Figure S3A** and **B**). The predicted models are nearly identical, with only minor deviations around residue V401. In contrast, the experimental structure differs primarily at residues Y306 and N346.

This deviation can be rationalized by the fact that the experimental structure was determined in complex with bound adenylated pyrrolysine, and pyrophosphate. Residue N346 is known to interact with the ester carbonyl O of pyrrolysine. Furthermore , the catalytic pockets of predicted structures of most of the *Methanosarcina* PylRS are almost identical, which agrees well with the highly conserved sequences in the *Methanosarcinales* order (data not shown).

To build a structural model of *M. burtonii* (**Figure 1B**), the crystal structure of the C- terminal domain of *Desulfitobacterium hafniense* (*D. hafniense*) PylRS (PDB ID: 2ZNI)^12^ was superimposed with the N-terminus (5UD5)^13^ of *M. mazei,* both in complex with their respective tRNA^Pyl^ molecules. Based on this alignment, the N- and C-terminal domains of *M. burtonii* (**Figure S4B**) were superimposed (**Figure S4C)**. The resulting *Mbur*PylRS structural model is shown in **Figure 1B**.


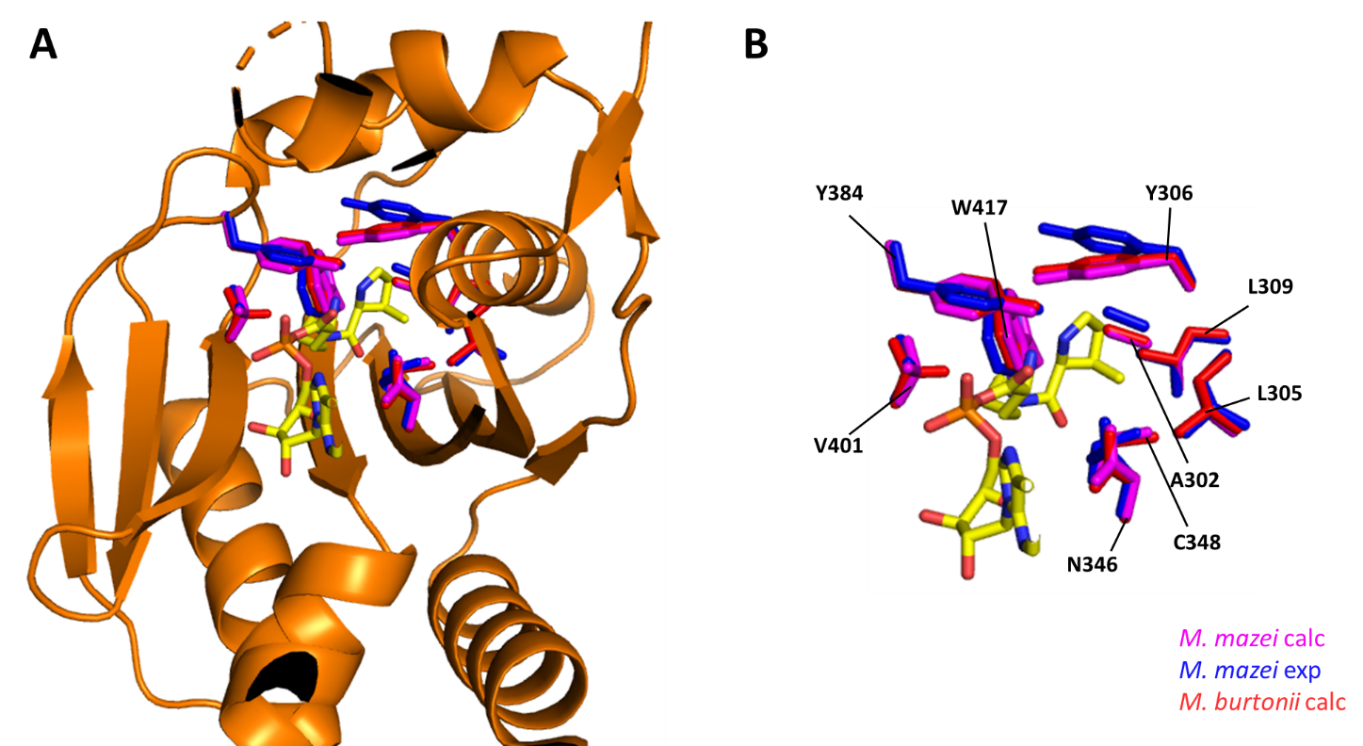


**Figure S3.** Overlay of the C-terminal domain of *M. mazei* (predicted = magenta, experimental determined = blue) and *M. burtonii* (red), with cartoon representation of the enzyme **A**) and without **B**).


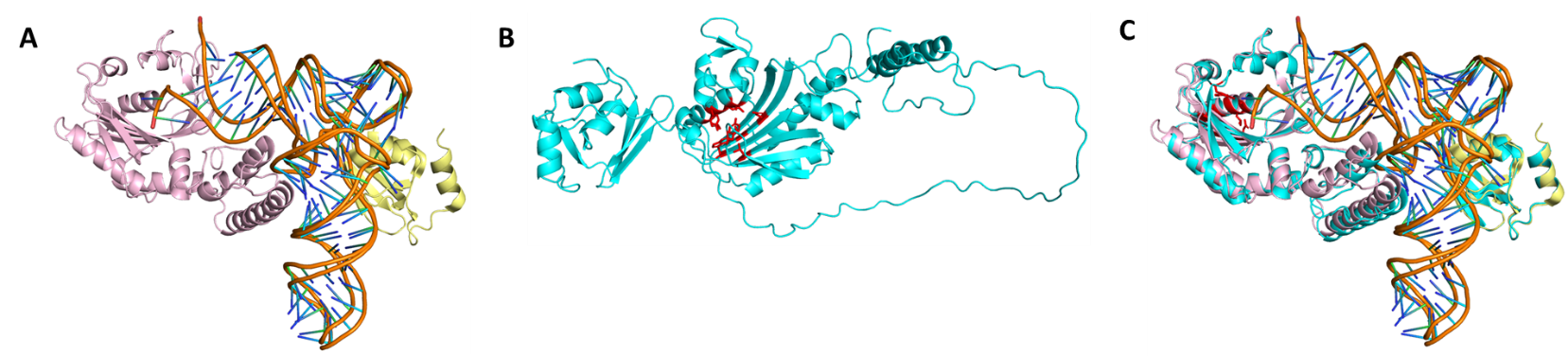


**Figure S4.** Experimental and predicted structures employed for the construction of the structural model depicted in **Figure 1B**. **A**) Superposition of C-terminus of *M. hafniense* (PDB ID: 2ZNI, light pink)^12^ bound to tRNA^Pyl^ with the N-terminus of *M. mazei* (PDB ID: 5UD5, light yellow) bound to tRNA^Pyl^. **B**) Predicted structure of *M. burtonii* (cyan). **C**) Overlay of **A**) and **B**) with deleted linker.

## Complete heatmaps for +N and ΔN PylRS variants

Comprehensive heatmaps for +N and ΔN PylRS variants are shown in **Figures S5** and **S6**. These datasets provide a concentration-resolved overview of ncAA incorporation efficiency across multiple substrates and constructs.

The +N variants generally exhibit higher overall fluorescence intensities and broader activity profiles across increasing ncAA concentrations (Figure S5). In contrast, ΔN variants display reduced maximal signals and narrower dynamic ranges (**Figure S6**), consistent with the performance trends observed in the main text.

Background suppression levels differed between +N and ΔN architectures but remained within defined construct-specific ranges, enabling comparative evaluation of relative activity patterns across substrates and concentration gradients.

**Figure S5:** Heatmaps of sfGFP(1x amber) expression in *E. coli* BL21(DE3) for eight selected +N PylRS variants across varying ncAA concentrations. Panels **A)**, **B)**, **C)**, **D)**, **E)**, **F)** correspond to 0.05, 0.1, 0,3, 1, 3, 9 mM ncAA supplementation, respectively. Bar charts including replicate number and standard deviations are provided in **Figures S25-S41**. Substrate numbering corresponds to **Figure 1**. Background suppression levels ranged from 900 to 1,300 [a.u.] depending on the construct.

**Figure S6:** Heatmaps of sfGFP(1x amber) expression in *E. coli* BL21(DE3) for15 selected ΔN PylRS variants. Substrate numbering corresponds to **Figure 1**. Background suppression levels were construct-dependent and ranged from 200 to 400 [a.u.].

## Cytosolic expression of PylRS variants determined by split-GFP assays

To evaluate whether Mbur tolerates N- or C-terminus sfGFP(11) tagging, we first generated a C-terminal tagged variant. As shown in **Figure S7**, C-terminal fusion of sfGFP(11) markedly reduced stop codon suppression efficiency using the sfGFP(1x amber) reporter.


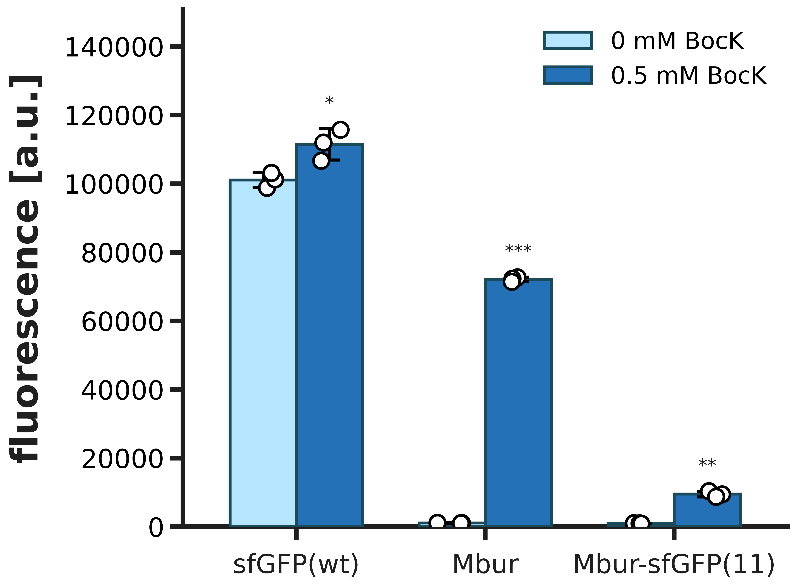


**Figure S7.** 96-well plate-based split-GFP assays to determine cytosolic PylRS abundance. Comparisons between 0 mM and 0.5 mM BocK conditions were analyzed using unpaired two-sample t-tests (Welch’s t-test; n = 3 biological replicates). Significance levels are indicated as follows: *P < 0.05, **P < 0.01, ***P < 0.001. Expression was performed with *E. coli* BL21(DE3) and sfGFP(1 x amber) for the Mbur and Mbur-sfGFP(11) variants.

Because C-terminal tagging substantially reduced *in vivo* activity, we next fused sfGFP(11) to the N-terminus of Mbur. Four different linker sequences were inserted between sfGFP(11) and Mbur.

Constructs were evaluated for: (i) Stop-codon suppression (SCS) efficiency (**Figure S8A**) to assess functional perturbation, and (ii) Fragment complementation efficiency between sfGFP(1‑10) and sfGFP(11) (**Figure S8B**).

To identify the optimal construct, both datasets were integrated to maximize fragment complementation while minimizing loss of SCS activity (**Figure S8C**).

To ensure robustness, this analysis was performed for both Mbur wild-type and a Sac-recognizing mutant. The optimal configuration contained Linker(2), corresponding to the (GGGGS)₃ sequence.


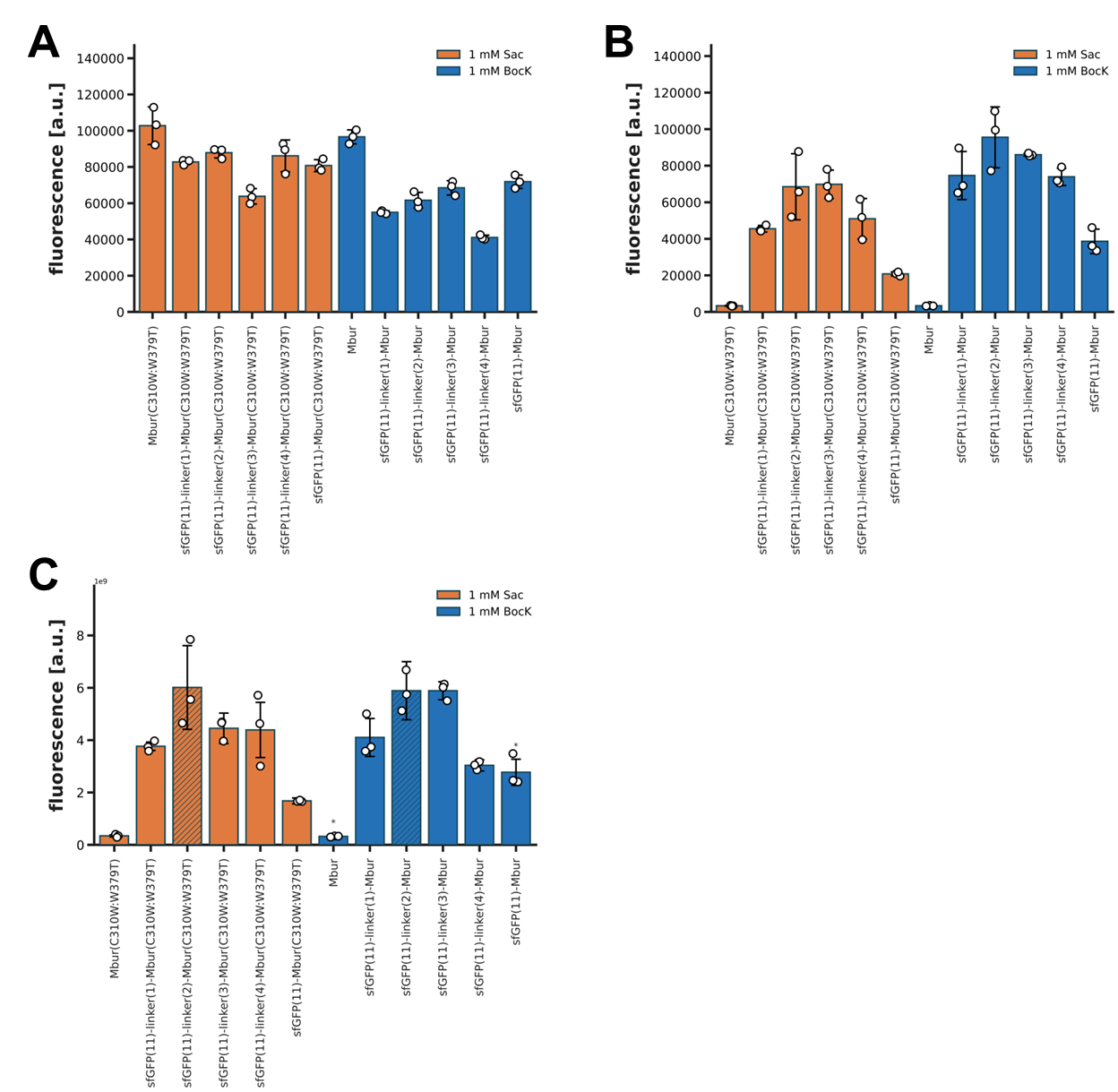


**Figure S8.** 96-well plate-based **A**) sfGFP (1x amber) suppression, **B**) split-GFP complementation, and **C** combined performance metrics . Statistical comparisons were performed between the highest-performing construct (striped) and all other constructs within each substrate condition (1 mM Sac or 1 mM BocK) using unpaired two-sample t-tests (Welch’s t-test; n = 3 biological replicates). P-values were adjusted using the Benjamini–Hochberg false discovery rate (BH/FDR) method. *P < 0.05. Expression was performed with *E. coli* BL21(DE3). Linker sequences: Linker(1) = (GSDGGSGGGSTS), which was from the original split-GFP publication^15^; Linker(2) = (GGGGS)_3_; Linker(3) = (GGGGS)_1_; Linker(4) = EGKSSGSGSESKST, which is an alternatively used flexible linker with better solubility than (GGGGS) linker^16^.


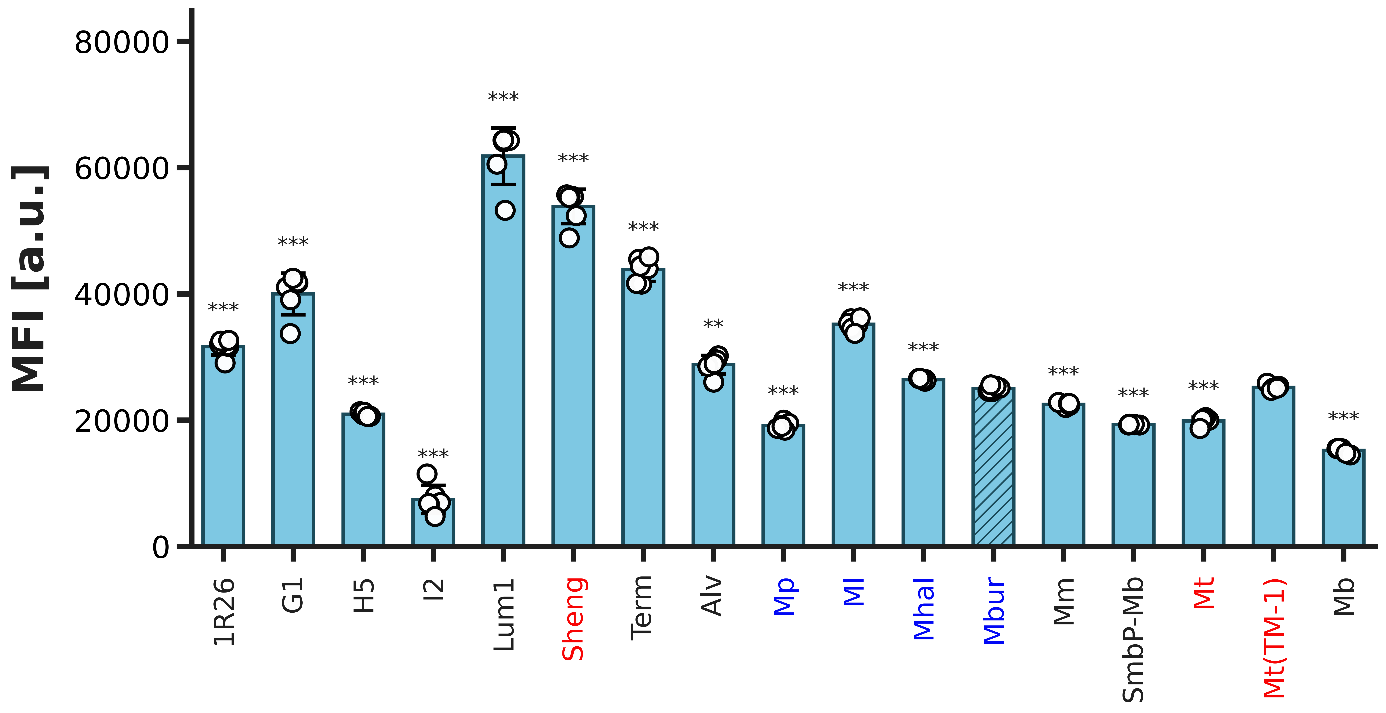


**Figure S9.** Flow cytometry-based split-GFP analysis to quantify cytosolic PylRS abundance. Median fluorescence intensity (MFI) of the positively gated population is shown. Statistical comparisons between Mbur (striped) and all other constructs were performed using unpaired two-sample t-tests (Welch’s t-test; n = 6 biological replicates). P-values were adjusted using the BH/FDR method. *P < 0.05, **P < 0.01, ***P < 0.001. Representative dot plots are provided in the supplementary flow cytometry section (**Figure S101**).


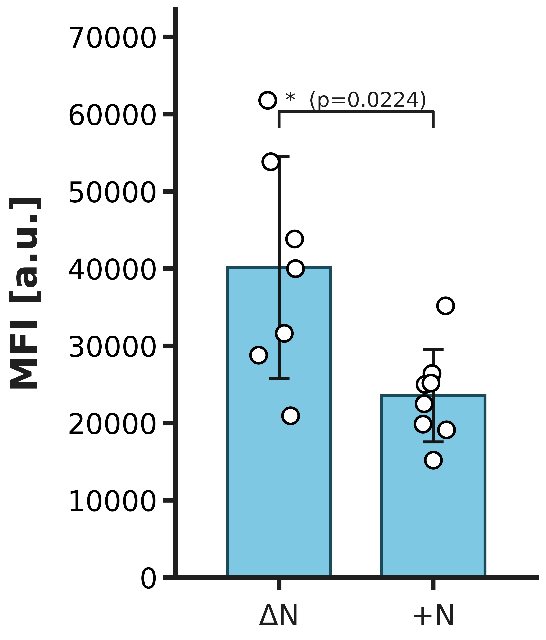


**Figure S10.** Statistical comparison of split-GFP data (**Figure S10**) for ΔN and +N PylRS groups. The SmbP-Mb construct was excluded because it represents an engineered +N variant with improved solubility. The ΔN PylRS I2 construct was excluded due to insufficient detectable cell population during flow cytometry (see main text). Statistical analysis was performed using unpaired two-sample t-tests (Welch’s t-test). The P-value is indicated in the figure. The mean cytosolic abundance differed by 58% between ΔN and +N PylRS groups.

## Inducible Promotor Validation

We evaluated the three strongest promotors that were reported from the “Marionette” collection^14^ and compared them to the constitutive *lpp* promotor. As shown in **Figure S7,** the P_CymRC_ promotor exhibited the highest expression strength among the tested Marionette promotors and showed activity comparable to the lpp promotor. For suppression of 5 amber stop codons, P_CymRC_ was the most efficient system under cultivation conditions with ncAA concentrations below 10 mM.


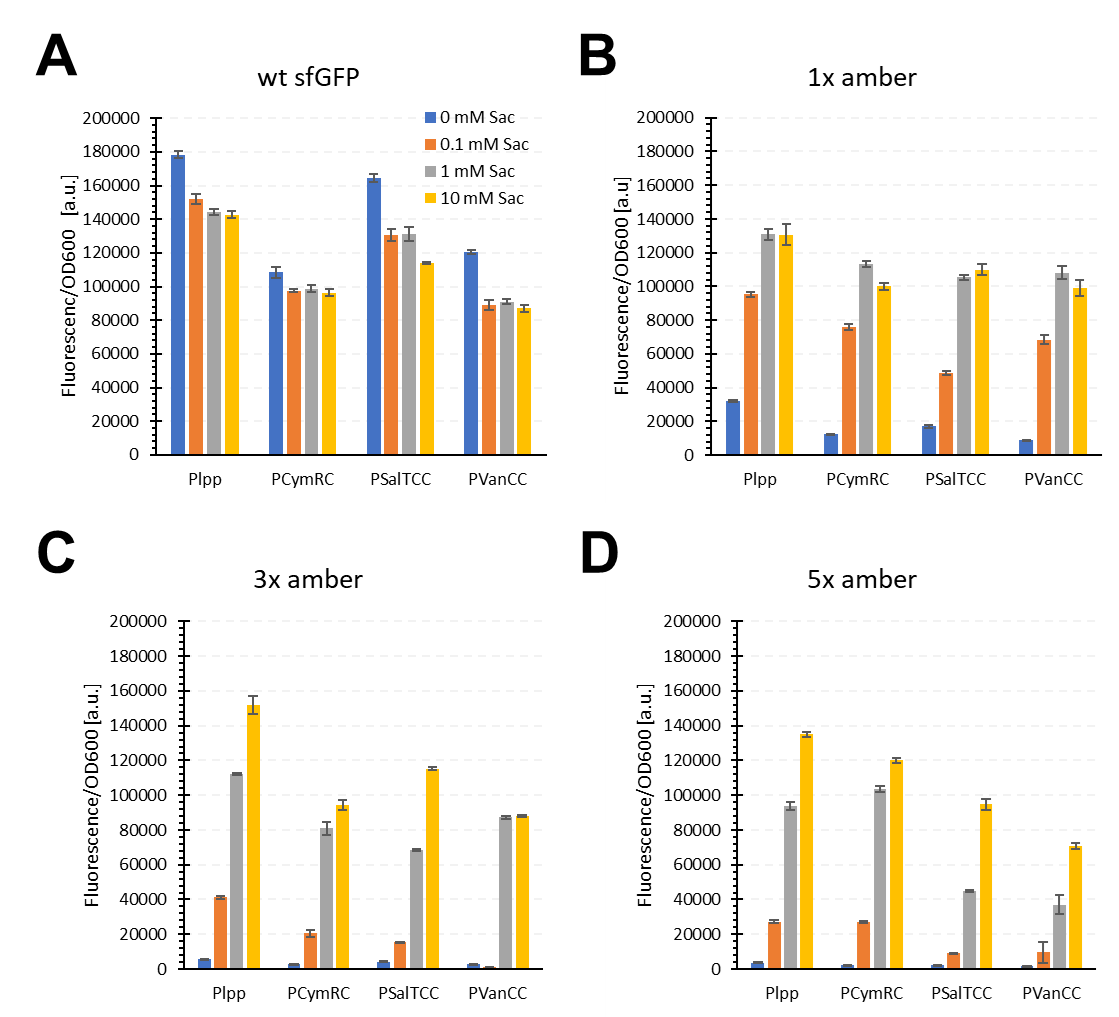


**Figure S11:** Comparison of different inducible promotors from the “Marionette” collection^14^ using the Mbur(C310W:W379T) variant. Fluorescence was measured in intact *E. coli* BL21(DE3) cells expressing the indicated reporter constructs, **A**) wild type sfGFP (no in-frame stop codon), **B**) sfGFP(1x amber), **C**) sfGFP(3x amber), **D**) sfGFP(5x amber). Data represent the mean ± standard deviation of three biological replicates (n = 3). ncAA abbreviations are listed in Section 3.1. Final inducer concentrations were 100 µM cuminic acid (P_CymRC_), 100 µM vanillic acid (P_VanCC_), and 100 µM sodium salicylate (P_SalTCC_).

## Comparison of pUltra and pTECH OTS setups

To evaluate the influence of vector architecture on orthogonal translation efficiency, we compared the pUltra and pTECH OTS systems using MburPylRS and substrate **2** (**Figure S12**). The standard pUltra configuration (**Figure S12 A**) shows a gradual increase in normalized fluorescence with increasing ncAA concentration. Replacing the inducible P_tac_ promoter with the constitutive lpp promoter (**Figure S12 B**) substantially enhances overall expression levels and results in improved performance across the tested concentration range.

The pTECH system (**Figure S12 C**) further increases suppression efficiency, reaching near-saturation levels at lower ncAA concentrations compared to the pUltra configurations. These results demonstrate that OTS architecture and promoter choice significantly influence system performance, particularly under RF1-deficient conditions.

Notably, differences between setups are most pronounced at intermediate ncAA concentrations, indicating that vector design impacts dynamic range rather than merely maximal expression capacity.


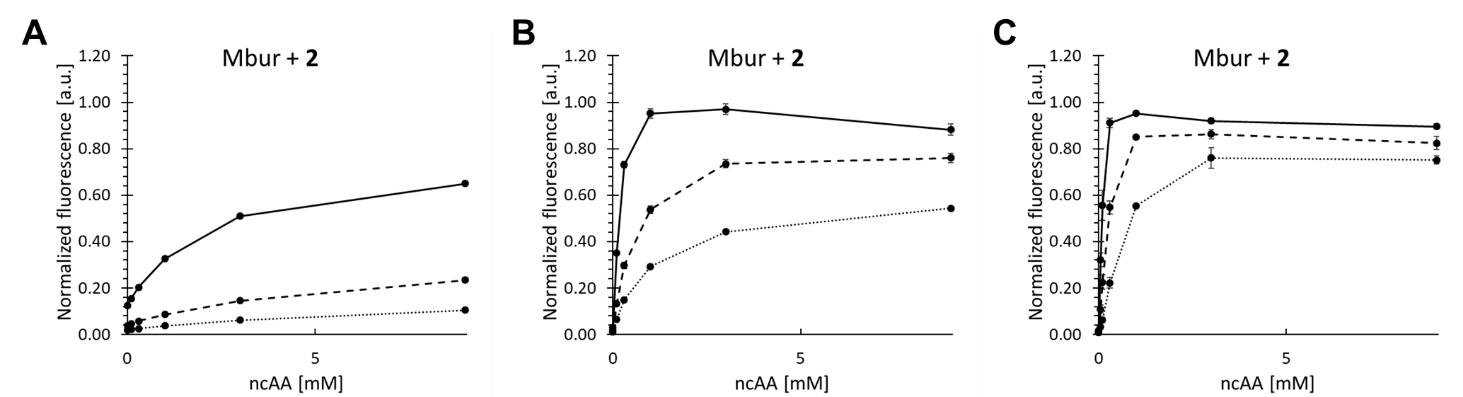


**Figure S12:** Concentration-dependent unnatural protein production using Mbur with substrate **2** under different OTS architectures. **A**) pUltra OTS setup, **B**) pUltra setup with the constitutive *lpp* promoter replacing P_tac_, **C**) pTECH OTS setup. Protein production was performed in RF1-deficient *E. coli* B-95.ΔA. Fluorescence values were normalized to the corresponding wild-type sfGFP reporter constructs (without in-frame stop codon). Error bars represent the standard deviation of three biological replicates.

## Multi-Site ncAA incorporation by +N and ΔN PylRS variants

To evaluate multi-site incorporation efficiency, the four best-performing +N and ΔN PylRS variants were tested in an RF1-deficient E. coli B-95.ΔA strain using reporter constructs containing multiple in-frame stop codons (**Figures S13** and **S14**).

All variants exhibit concentration-dependent increases in normalized fluorescence, with performance generally plateauing between 1 and 3 mM ncAA. The +N variants display higher overall suppression efficiencies across the tested concentration range compared to ΔN variants, particularly at intermediate ncAA concentrations.

These results confirm that the enhanced activity observed for selected +N PylRS variants in single-site suppression experiments is retained under multi-site incorporation conditions in an RF1-deficient background.


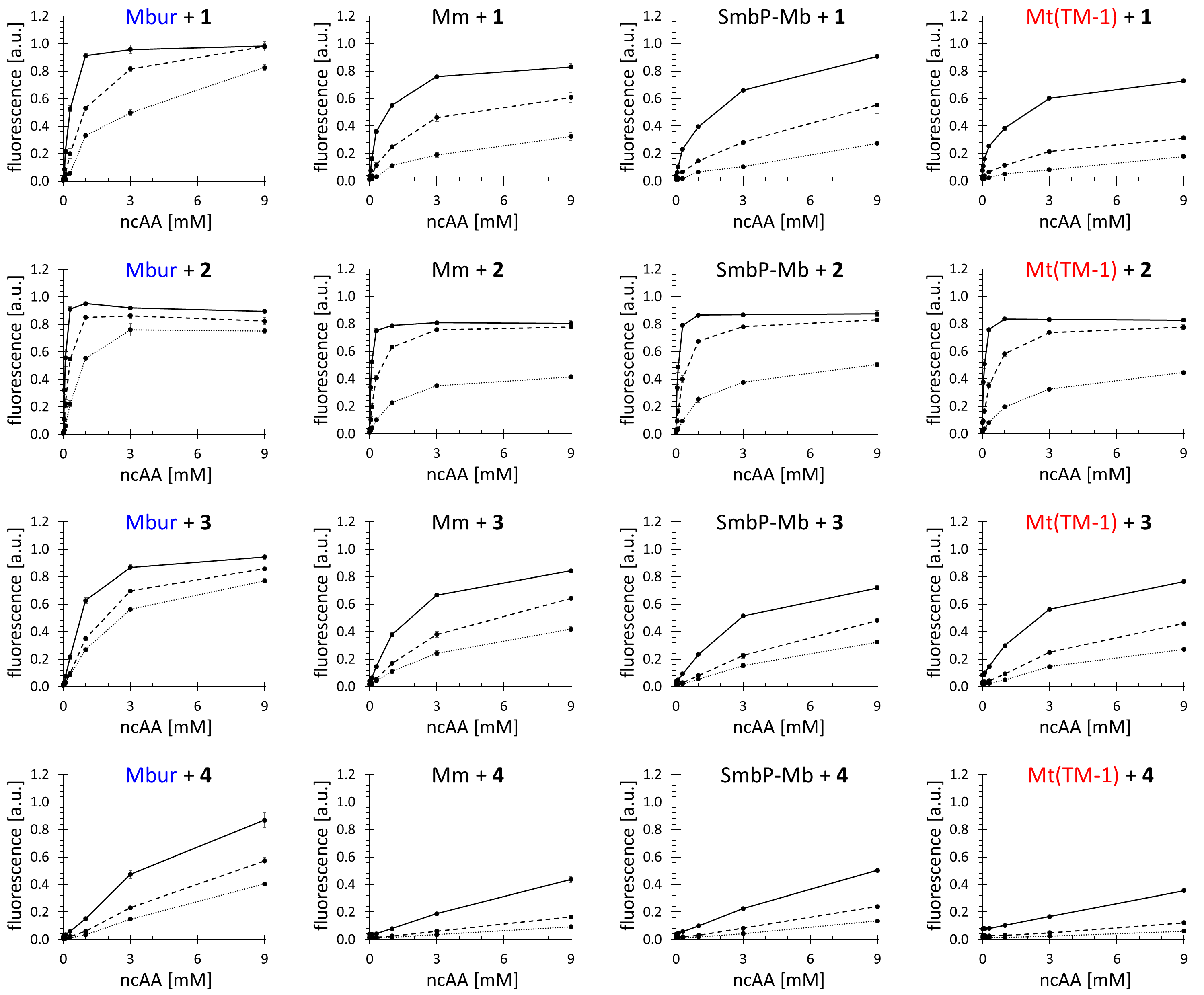


**Figure S13:** Concentration-dependent unnatural protein production using the four best +N PylRS variants (identified in **Figure 2**). Protein production was performed in RF1 deficient *Escherichia coli* B-95.ΔA. Endpoint measurements for ncAA concentrations of 0.05, 0.1, 0.3, 1, 3 and 9 mM. Fluorescence values were normalized their corresponding wild-type sfGFP reporter constructs (without in-frame stop codon). Error bars represent the standard deviation of three biological replicates. Substrates and PylRS constructs are indicated in the panel headings .


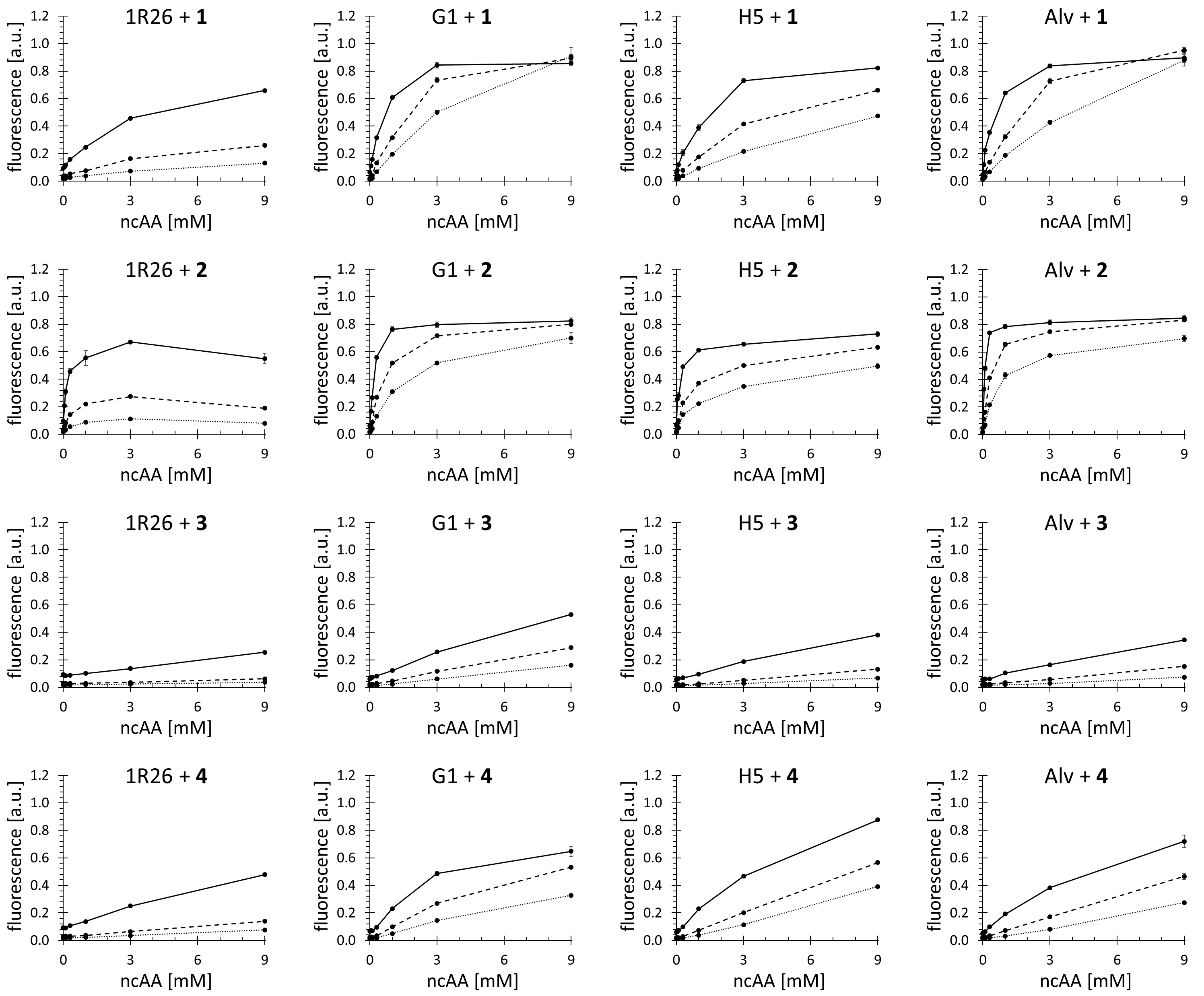


**Figure S14A:** Concentration-dependent unnatural protein production using the four best ΔN PylRS variants (identified in **Figure 2**). Protein production was performed in RF1 deficient *Escherichia coli* B-95.ΔA. Endpoint fluorescence measurements were obtained at ncAA concentrations of 0.05, 0.1, 0.3, 1, 3 and 9 mM. Fluorescence values were normalized to the corresponding wild-type sfGFP reporter constructs (without in-frame stop codon). Error bars represent the standard deviation of three biological replicates. Substrates and PylRS constructs are indicated in the panel headings.


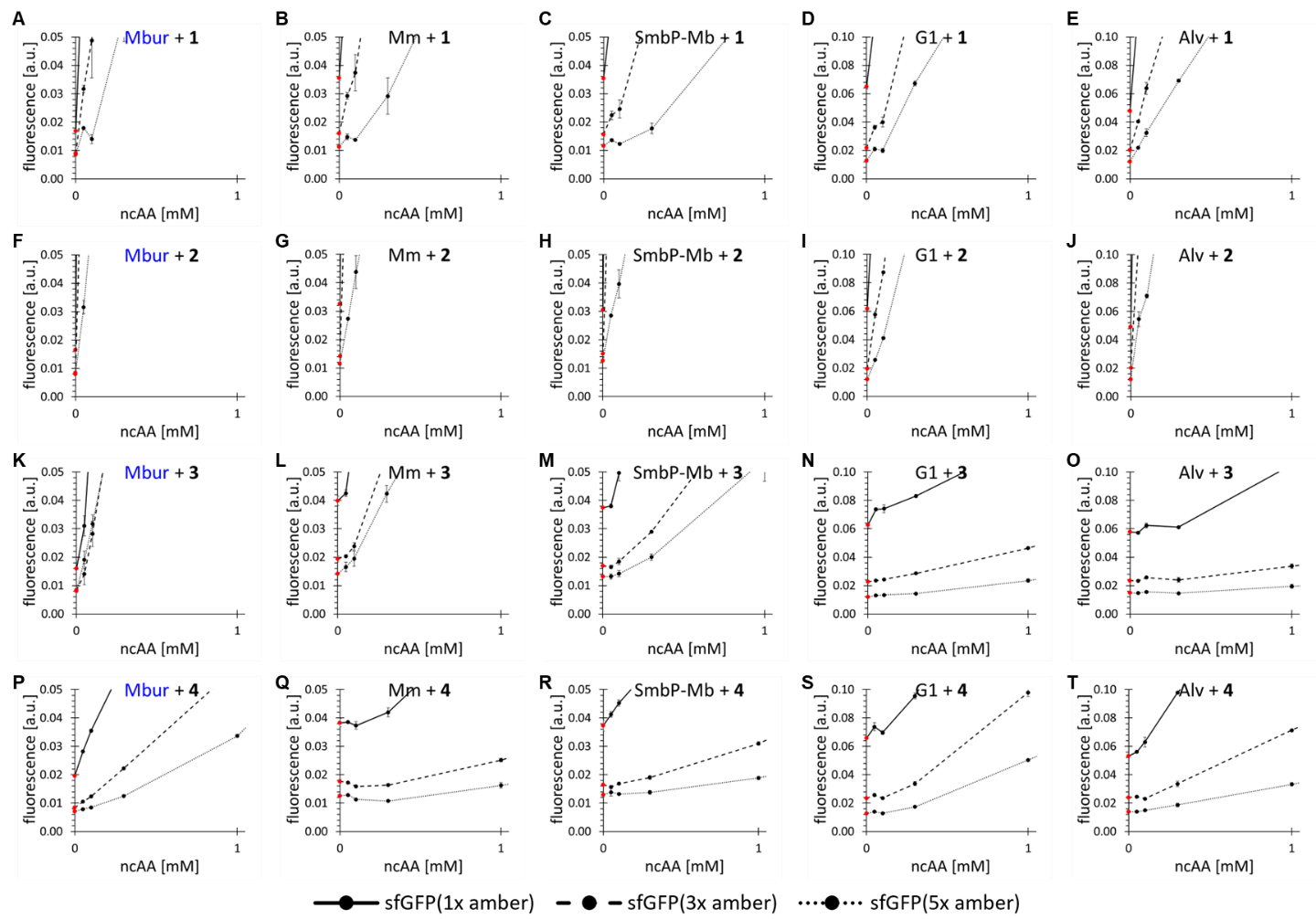


**Figure S14B:** Concentration-dependent sfGFP production using the two best performing +N and ΔN PylRS variants from **Figure 2,** compared with Mbur. Protein expression was performed in RF1 deficient *Escherichia coli* B-95.ΔA. Endpoint measurements were performed at ncAA concentrations (x-axis) of 0.05, 0.1, 0.3, 1, 3 and 9 mM. Fluorescence values were normalized to the corresponding wild-type sfGFP reporter constructs (without an in-frame stop codon). Error bars represent the standard deviation of three biological replicates (n=3). Fluorescence intensity (y-axis) is reported in arbitrary units (a.u.). Red dots are highlighted datapoint at 0 mM ncAA concentration indicating background suppression.

## Temperature-dependent expression of wild-type sfGFP and background suppression

To assess temperature-dependent background suppression and overall expression capacity, we compared sfGFP(5× amber) expression in the absence of ncAA supplementation and wild-type sfGFP expression at 37 °C and 18 °C (**Figure S15**).

In the absence of ncAA (Panel A), background fluorescence levels are low across all constructs, with no evidence of substantial temperature-dependent increases in nonspecific suppression. This indicates that enhanced activity observed under supplemented conditions is not driven by elevated background readthrough at lower temperatures.

Wild-type sfGFP expression (Panel B) shows reduced overall fluorescence at 18 °C compared to 37 °C, consistent with slower cellular growth and protein production at lower temperatures. Importantly, relative differences between PylRS variants remain consistent across temperatures, supporting the conclusion that temperature-dependent performance differences reflect intrinsic OTS properties rather than global expression artifacts.
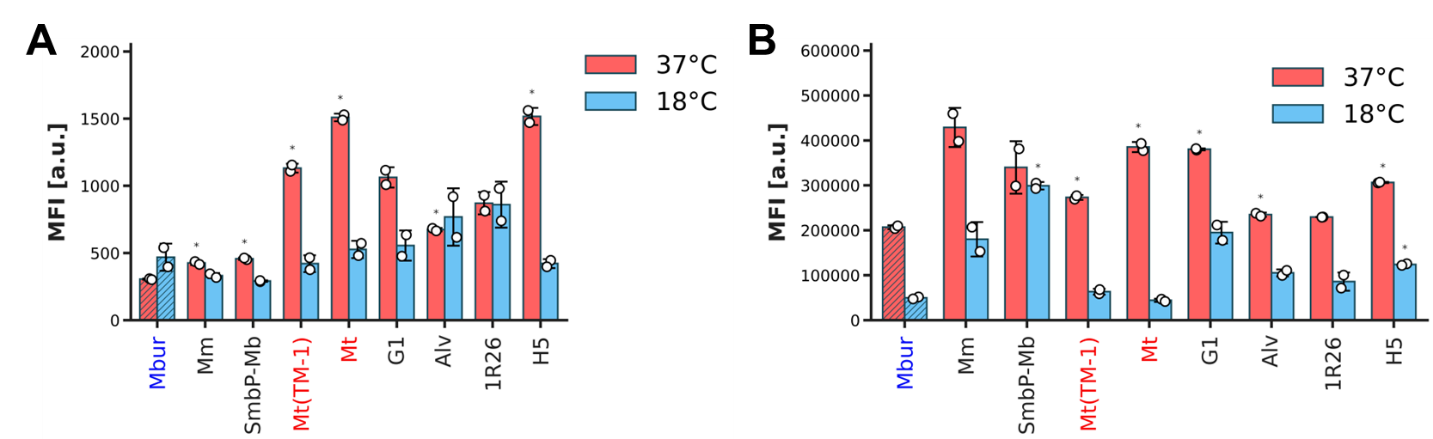


**Figure S15:** Temperature dependent protein production in RF1-deficient *E. coli* B-95.ΔA. **A**) sfGFP(5x amber) expression in the absence of supplied ncAA; **B**) wild-type sfGFP expression (no in-frame stop codon). . Cells were cultivated at 37 °C (red bars) or 18 °C (blue bars). Constructs were analyzed by flow cytometry in biological duplicates (n=2) and the median fluorescence intensity (MFI) of the gated population is shown. Statistical comparisons between Mbur (striped bars) and all other constructs within each temperature group were performed using Welch’s t-tests. P-values were adjusted using the Benjamini–Hochberg false discovery rate (BH/FDR) method. *P < 0.05, **P < 0.01, ***P < 0.001.

## Full Heatmaps for double Alanine and Glycine PylRS constructs

To further evaluate the impact of active-site modifications, selected double alanine and double glycine PylRS mutants were screened for ncAA incorporation efficiency using the sfGFP(1× amber) reporter at 10 mM ncAA (**Figure S16**).

The heatmaps provide a comparative overview of substrate-dependent activity patterns across variants. While individual substitutions alter performance profiles, no uniform gain-of-function trend is observed across all substrates. Instead, activity changes appear substrate-specific, indicating that double mutations differentially affect active-site compatibility rather than globally enhancing catalytic efficiency.

Background suppression levels varied between constructs but remained within a comparable range, allowing relative activity differences to be assessed across variants.

**Figure S16:** Heatmaps representation of sfGFP(1x amber) expression in *E. coli* BL21(DE3) using selected double alanine and double glycine PylRS mutants. Fluorescence measurements were performed at 10 mM ncAA. Substrate numbering corresponds to **Figure 1**. Background suppressions levels were construct-dependent and ranged from 1,100 to 2,500 [a.u.].

## Additional data for promiscuity analysis

To further characterize substrate scope, selected PylRS variants were evaluated for incorporation of structurally diverse charged and polar ncAAs (Figure S17).

Distinct activity patterns were observed across variants. While several constructs exhibit measurable incorporation of multiple substrates at 10 mM supplementation, the magnitude of fluorescence varies considerably between ncAAs. In general, variants optimized for aromatic substrates display reduced efficiency toward more polar or charged analogues, consistent with active-site specificity constraints.

Titration experiments with canonical tyrosine at lower concentrations (0.1–1.5 mM) provide a reference for background incorporation levels and illustrate the relative efficiency differences across substrates. Overall, the data indicate that substrate promiscuity remains limited and variant-dependent, rather than reflecting broad nonspecific activation.


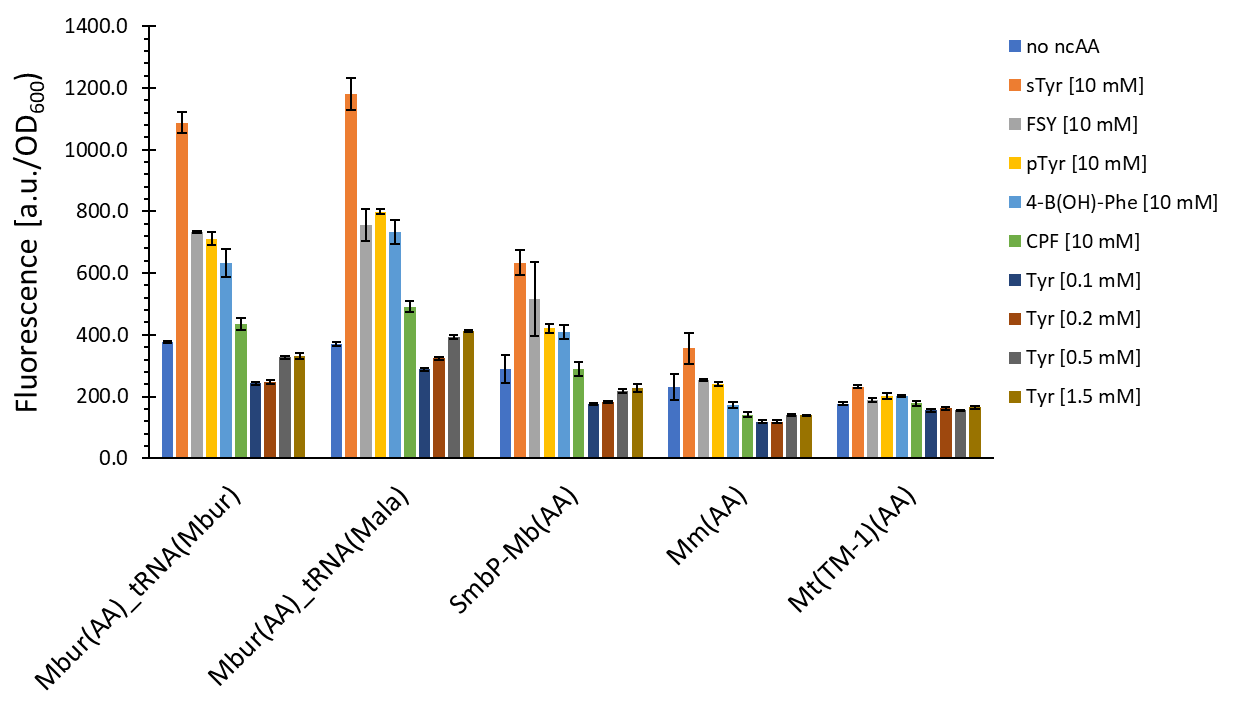


**Figure S17:** Incorporation of charged and polar ncAA by selected PylRS variants. Fluorescence was measured in intact *E. coli* BL21(DE3) cells expressing the sfGFP(1x amber) reporter. Data represents the mean ± standard deviation of three biological replicates (n=3). ncAA abbreviations are listed in Section 3.1, Table S4.

## Testing PylRS constructs for SproC (40) and Sac (43) incorporation

For Sac supplementation (Panel A), SmbP-MbSacRS(S382T) and MmSacRSopt(S417T) show robust concentration-dependent increases in fluorescence, reaching saturation at higher ncAA concentrations. In contrast, MburSacRS(S379T) exhibits a lower maximal signal under these conditions.

For SproC supplementation (Panel B), overall incorporation efficiencies are reduced compared to Sac, with all variants showing lower fluorescence levels across the tested concentration range. Among the constructs, SmbP-MbSacRS(S382T) displays the highest activity at intermediate concentrations, whereas the other variants show more modest responses.

These results indicate that substrate-dependent differences in incorporation efficiency are strongly influenced by the specific SacRS variant and highlight distinct activity profiles for Sac and SproC.


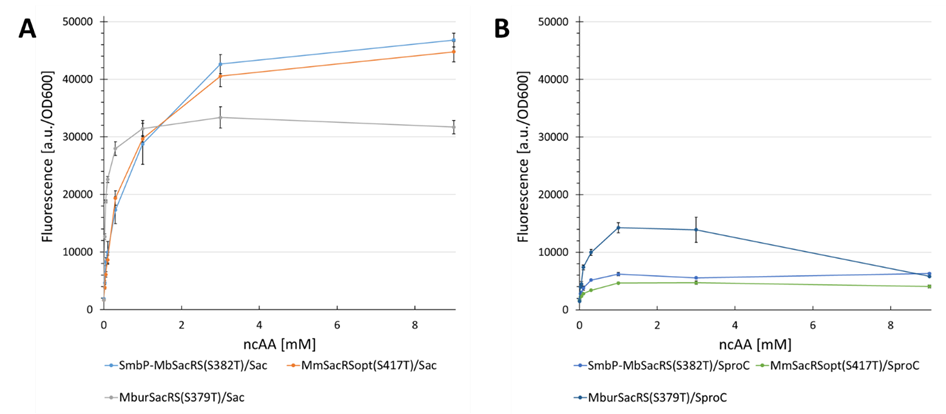


**Figure S18.** Concentration-dependent unnatural protein production for SacRS variants supplemented with **A**) Sac (**38**). **B**) SproC (**35**). Experiments were performed in *E. coli* BL21(DE3) cells and sfGFP(1x amber) reporter. Endpoint fluorescence measurements were obtained at r ncAA concentrations of 0.025, 0.05, 0.1, 0.3, 1, 3 and 9 mM. Error bars represent the standard deviation of three biological replicates.

## Multi-Site ncAAs incorporation in BL21(DE3)

To compare the efficiency of Mbur with the best known PylRS OTS Mm) two substrates (**2** and **4**) were tested in conjunction with sfGFP reporter constructs containing between one and five stop-codons.


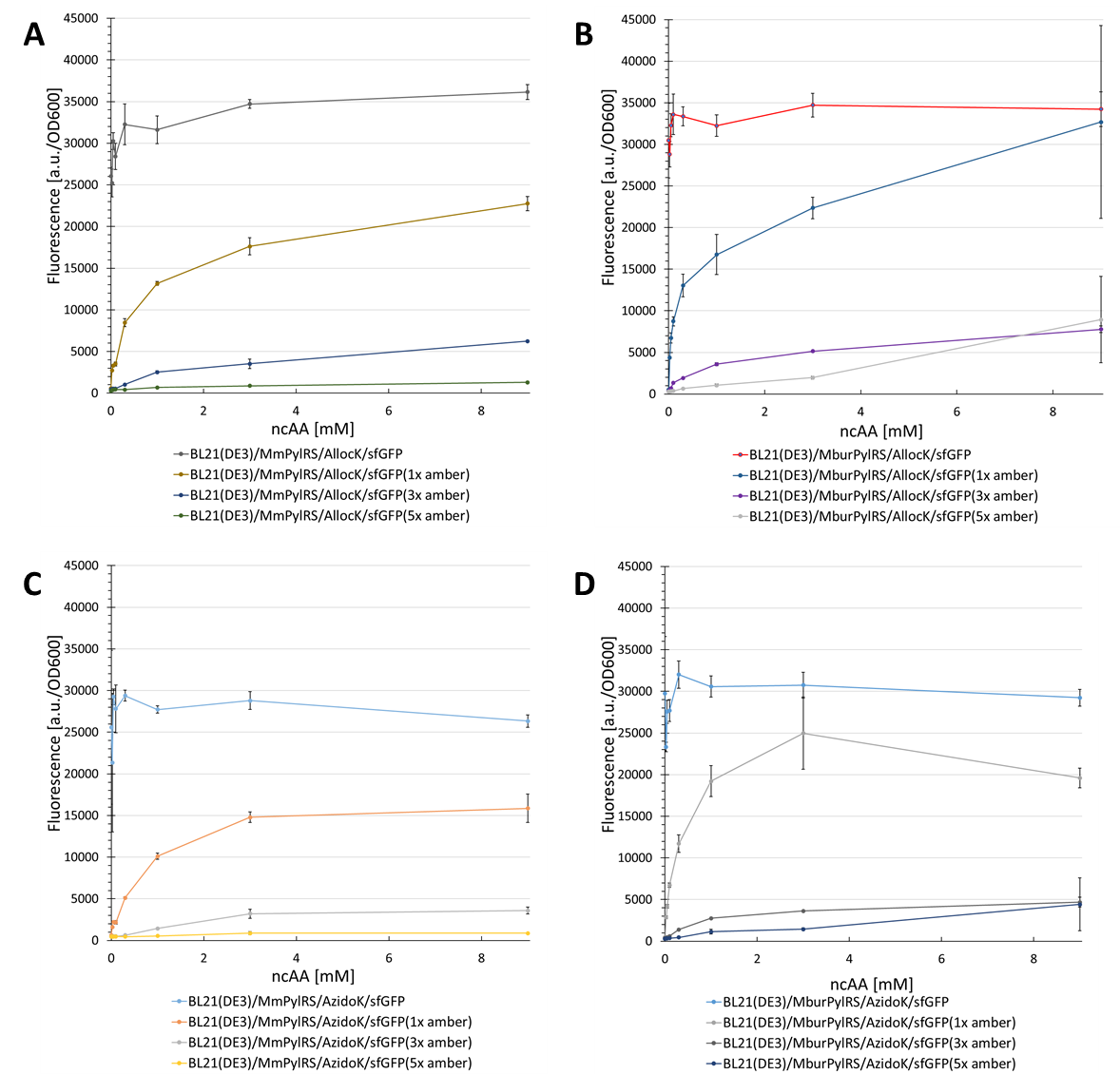


**Figure S19.** Concentration-dependent unnatural protein production with *Mm*PylRS (**A**, **C**) and *Mbur*PylRS (**B**, **D**) for different ncAA/reporter construct combinations.The host for protein production was BL21(DE3). Endpoint measurements for ncAA concentrations of 0.025, 0.05, 0.1, 0.3, 1, 3 and 9 mM.

**Figure S19** shows that for BL21(DE3) expression of sfGFP(1x amber), the performances of both PylRS are comparable when supplied with AllocK(**2**). In the same setup with AzidoK(**4**), the *Mbur*PylRS outperforms *Mm*PylRS, and is twice as efficient. With the suppression of more than one stop-codon in the BL21(DE3) strain, the performance decreases significantly, but the *Mbur*PylRS shows at least twice the efficiency than the *Mm*PylRS in the range between 1 and 3 mM ncAA, albeit at a low level. For protein production in the B95.ΔA strain, the suppression of one stop-codon is similar to the BL21(DE3) experiment, but with generally higher suppression efficiencies (**Figure S19**). Encouragingly, it is possible with *Mbur*PylRS to achieve wild-type level protein production for both ncAAs when fed 1 mM. A comparison of the OTS performances with *Mbur*PylRS/AllocK (**2**) shows that the decrease in suppression efficiency of one to five stop-codons, when supplied with 1 mM, is 49%. With *Mm*PylRS, the decrease is 77%.

The performance does not decrease as much when more than one stop codon is suppressed compared to BL21(DE3), proving the advantage of employing *Mbur*PylRS. At an AzidoK(**4**) concentration of 1 mM and suppression of three and five stop-codons, the performance is three times better than that of *Mm*PylRS. This result suggests that *Mbur*PylRS is not only very efficient at low ncAA concentrations, but also more suitable for incorporation of ncAAs at multiple sites. This advantage might be even more pronounced in the absence of RF‑1 competition, for example, in organisms with liberated codons or with sense codon suppression.

## Multi-Site Incorporation of Sac (43) and SproC (40)

Based on the high efficiency of the SacRS variants with the S→T mutation^17^, the *M. barkeri* and *M. burtonii* variants were investigated for the multi-site incorporation of ncAAs with two different *E. coli* strains. The *M. barkeri* variant instead of the *M. mazei* variant was chosen, because of slightly better performance with SproC (**40**) (and equal Sac (**43**) activity). For reporter protein production in BL21(DE3) with Sac (**43**), the relative performance of *Mbur*SacRS(S379T) compared to SmbP‑*Mb*SacRS(S382T) increases with the number of in-frame stop-codons (**Figure S20**). With one stop codon, the performance is 230% higher (at 0.3 mM), with three stop codons it is 490% higher (also at 0.3 mM), and five stop codons result in no incorporation with SmbP‑*Mb*SacRS(S382T).


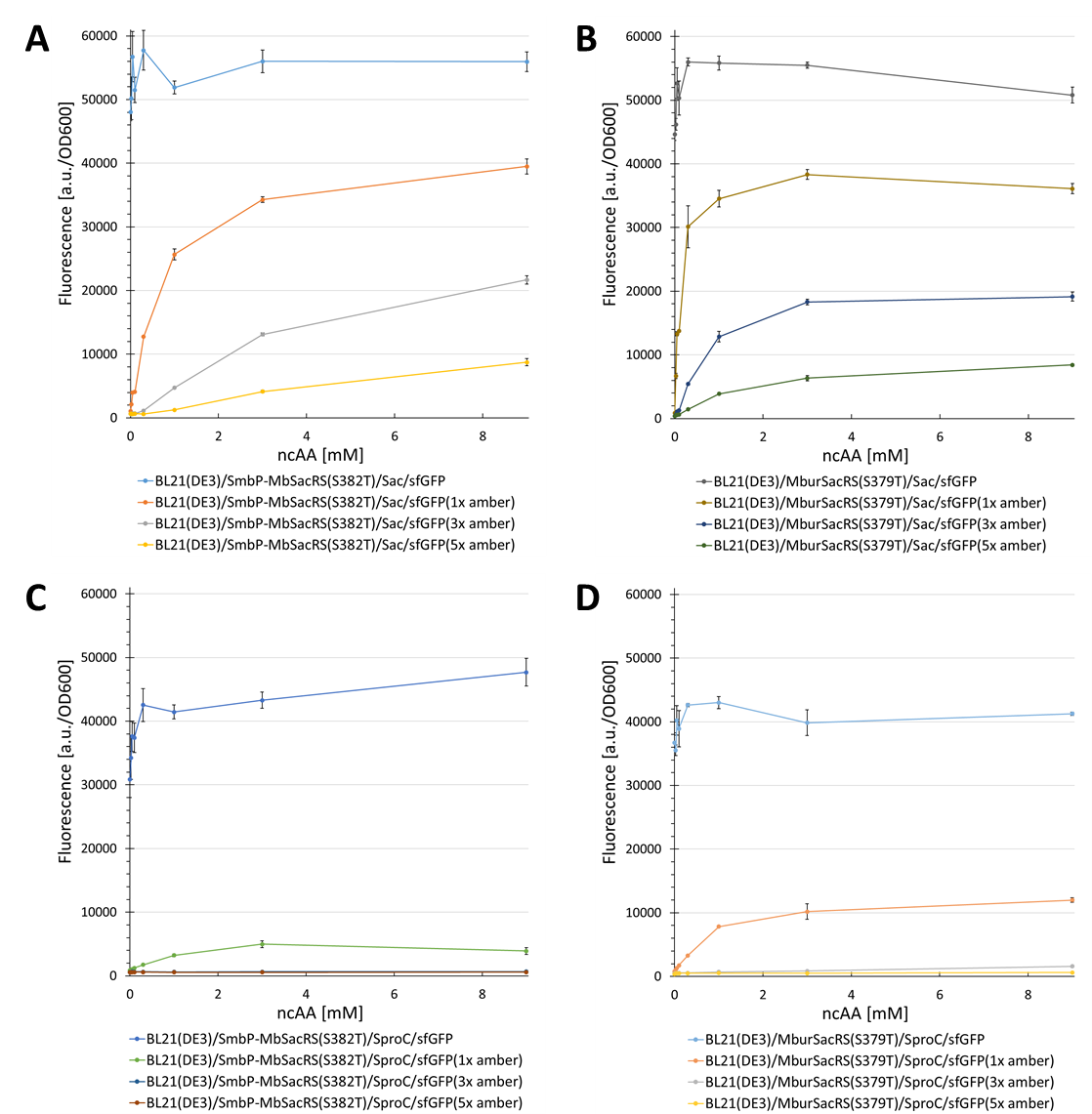


**Figure S20.** Concentration-dependent unnatural protein production with SmbP-*Mb*PylRS (**A**, **C**) and *Mbur*PylRS (**B**, **D**) for different ncAA/reporter construct combinations. The host for protein production was BL21(DE3). Endpoint measurements for ncAA concentrations of 0.025, 0.05, 0.1, 0.3, 1, 3 and 9 mM.

The same trends are observed in the B-95.ΔA strain, with the difference that both OTS show significantly higher incorporation efficiencies when more than one in-frame stop codon is suppressed (**Figure S21**). But as with other *Mbur*PylRS variants, the efficiency is much higher at low ncAA concentrations and surprisingly, the decrease in incorporation efficiency with increasing number of in-frame stop codons is extremely low (for Sac (**43**)). A comparison of the OTSs performances with Sac (**43**) shows that the decrease for the *Mbur*SacRS(S379T) construct (fed with 1 mM) is 25% from the suppression of one to five in-frame stop codons. For SmbP‑*Mb*SacRS(S382T) the decrease is 72%. As mentioned above, the SCS efficiency of *Mbur*SacRS(S379T) is at the same level as the wild-type enzyme when suppressing one stop codon, but when incorporating ncAAs at multi-sites, this mutant even surpasses the wild-type performance (compare **Figure 19** for the good substrate AllocK (**2**)). A PylRS mutant with better catalytic efficiency than the wild-type has never been reported before.


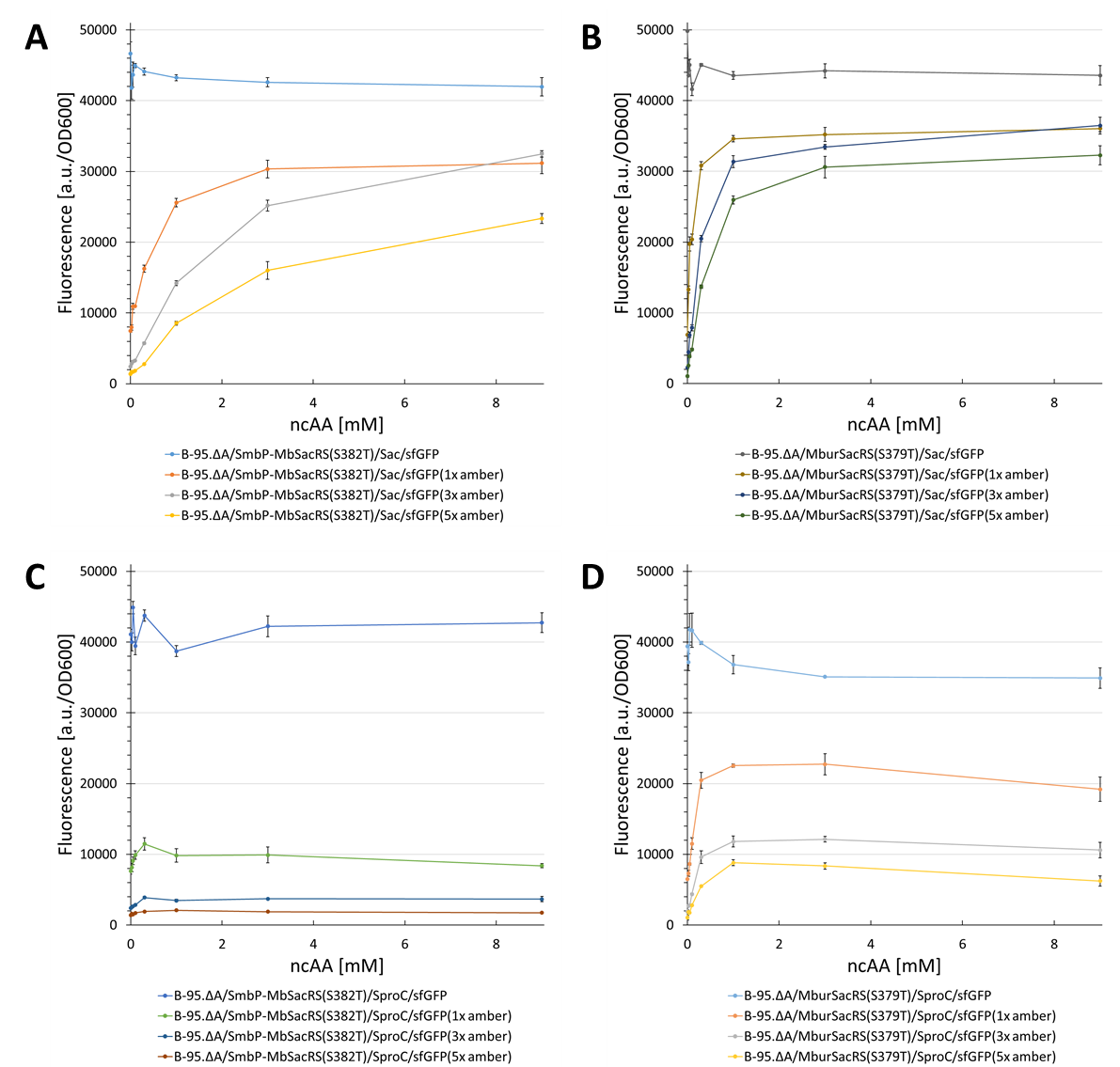


**Figure S21.** Concentration-dependent unnatural protein production with SmbP-*Mb*PylRS (**A**, **C**) and *Mbur*PylRS (**B**, **D**) for different ncAA/reporter construct combinations. The protein production host was B-95.ΔA. Endpoint measurements for ncAA concentrations of 0.025, 0.05, 0.1, 0.3, 1, 3 and 9 mM.

## Comparison of *Mj*TyrRS and *Mbur*PylRS performance


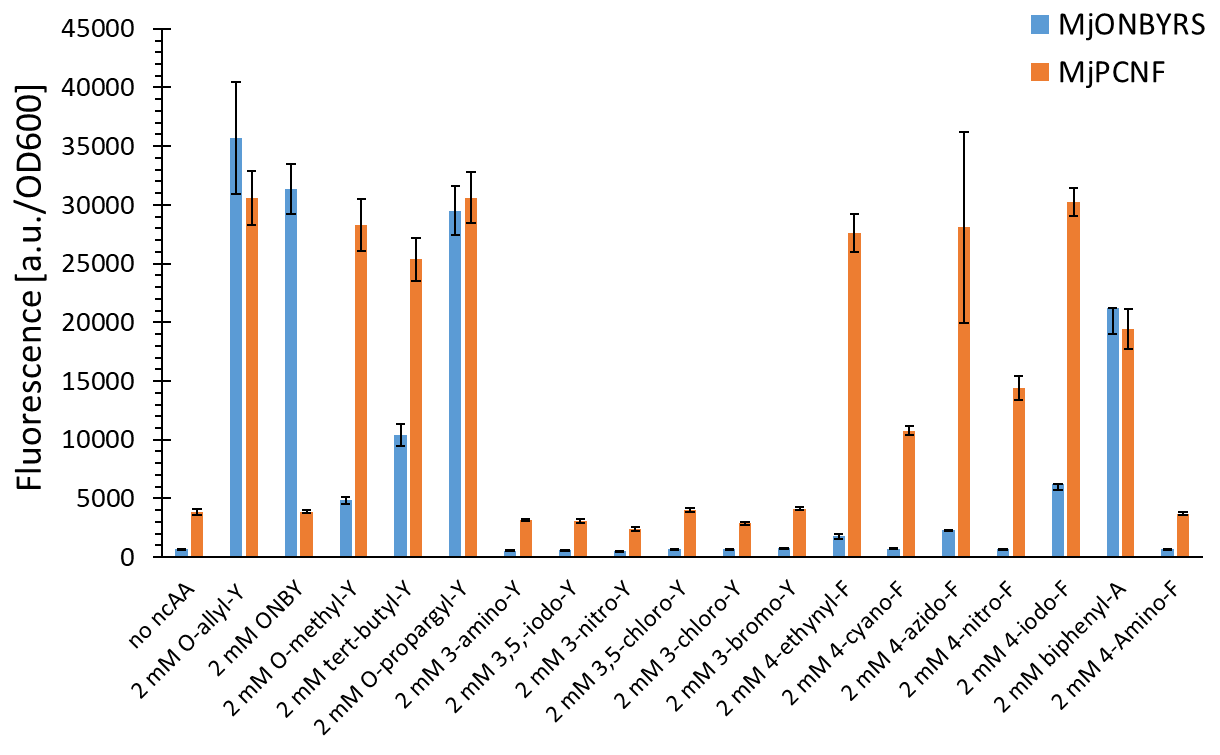


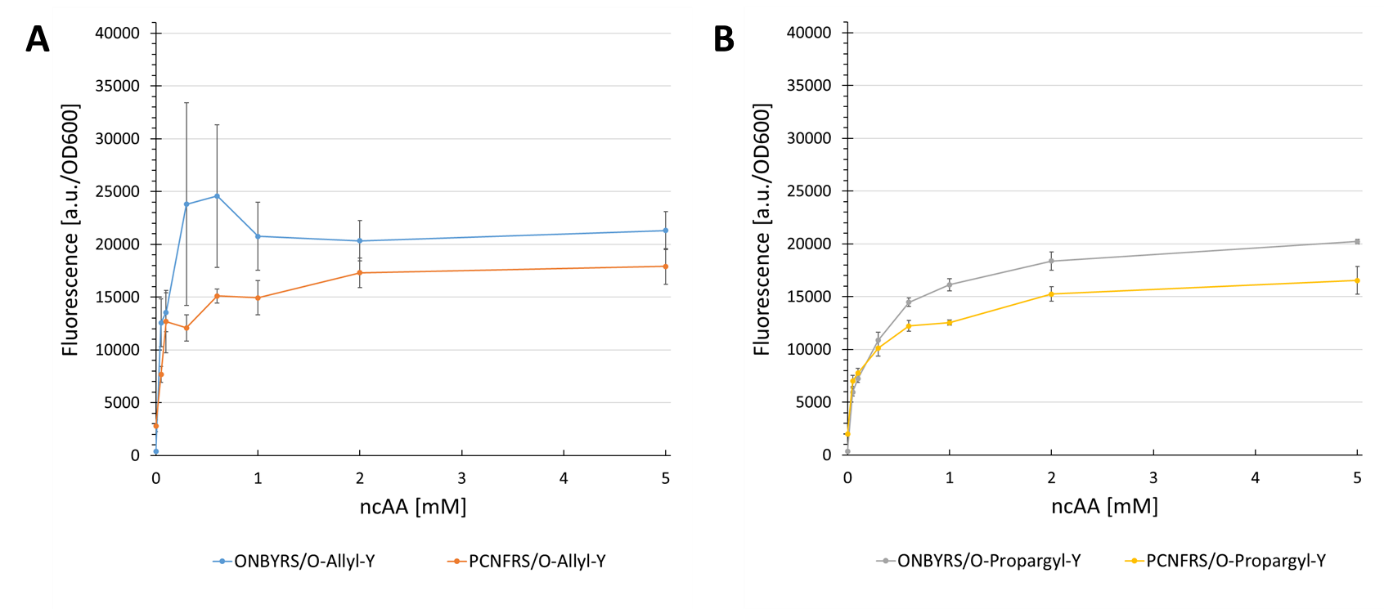
**Figure S22.** Prescreening of several ncAAs with MjONBYRS and MjPCNFRS. Fluorescence intensity of intact E. coli BL21(DE3) cells expressing the SUMO-sfGFP(1x amber) reporter. Endpoint measurements after 24 h with 2 mM ncAAs supplied. The data including the standard deviation represent the mean of three biological replicates.

**Figure S23.** Concentration-dependent protein production for two different MjTyrRS/ncAA combinations. Fluorescence intensity of intact E. coli BL21(DE3) cells expressing the SUMO-sfGFP(1x amber) reporter. Endpoint measurements after 24 h with different ncAA concentrations (0.025, 0.05, 0.1, 0.3, 1, 2, and 5 mM). The data including the standard deviation represent the mean of three biological replicates.


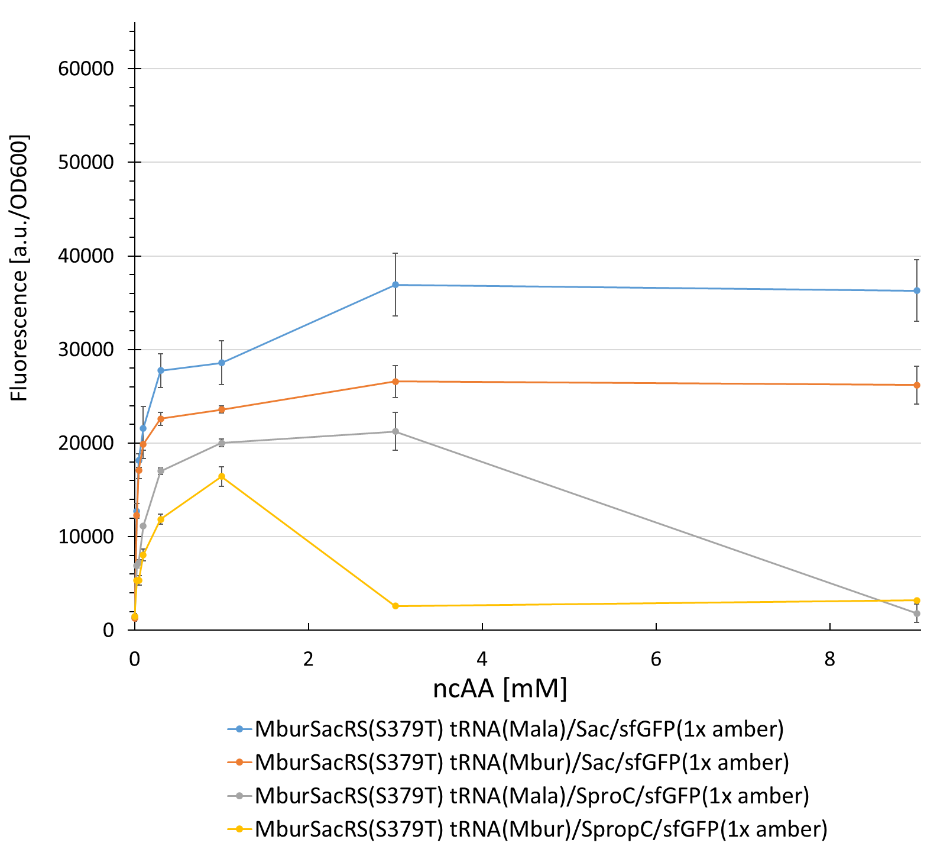


**Figure S24.** Concentration-dependent unnatural protein production with *Mbur*PylRS and different tRNA(organism abbreviation)/ncAA combinations. Using BL21(DE3) cells and sfGFP(1x amber) as reporter. Endpoint measurements for ncAA concentrations of 0.025, 0.05, 0.1, 0.3, 1, 3 and 9 mM.

# Supplementary Data, DNA sequences and mass-profiles of intact ncAA-containing proteins

## Fluorescence Assays on the influence of ncAA concentration on PylRS-based OTS


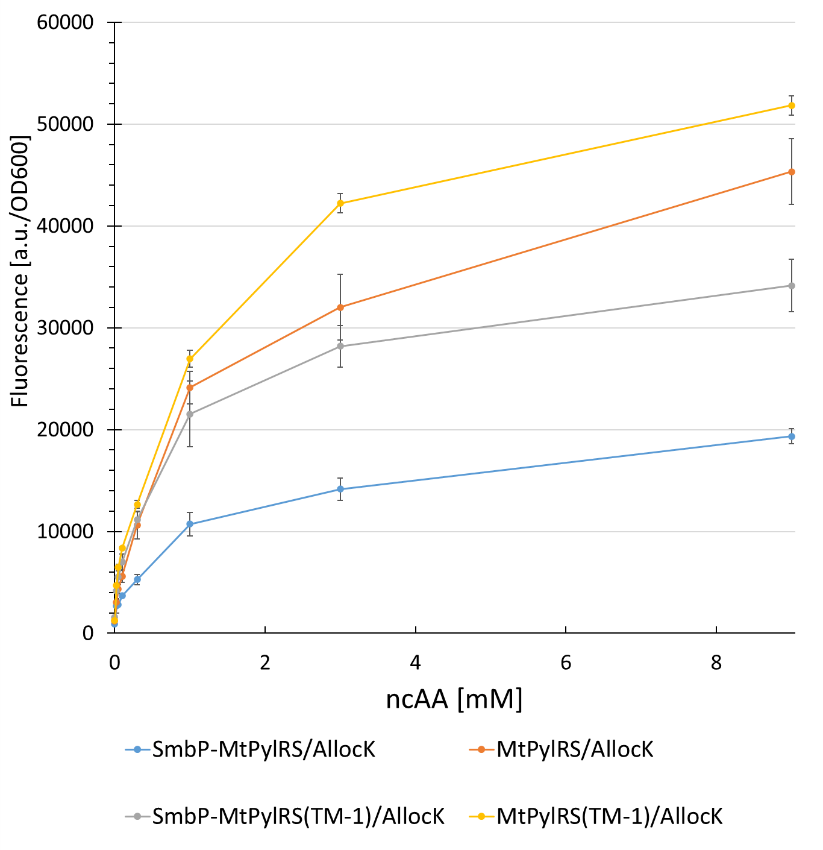


**Figure S25.** Concentration-dependent protein production for four different PylRS variants. Fluorescence intensity of intact *E. coli* BL21(DE3) cells expressing the SUMO-sfGFP(R2 amber) reporter, endpoint measurements after 24 h with different ncAA concentrations (0.025, 0.05, 0.1, 0.3, 1, 3, and 9 mM). Data including standard deviation represents the mean of three biological replicates.


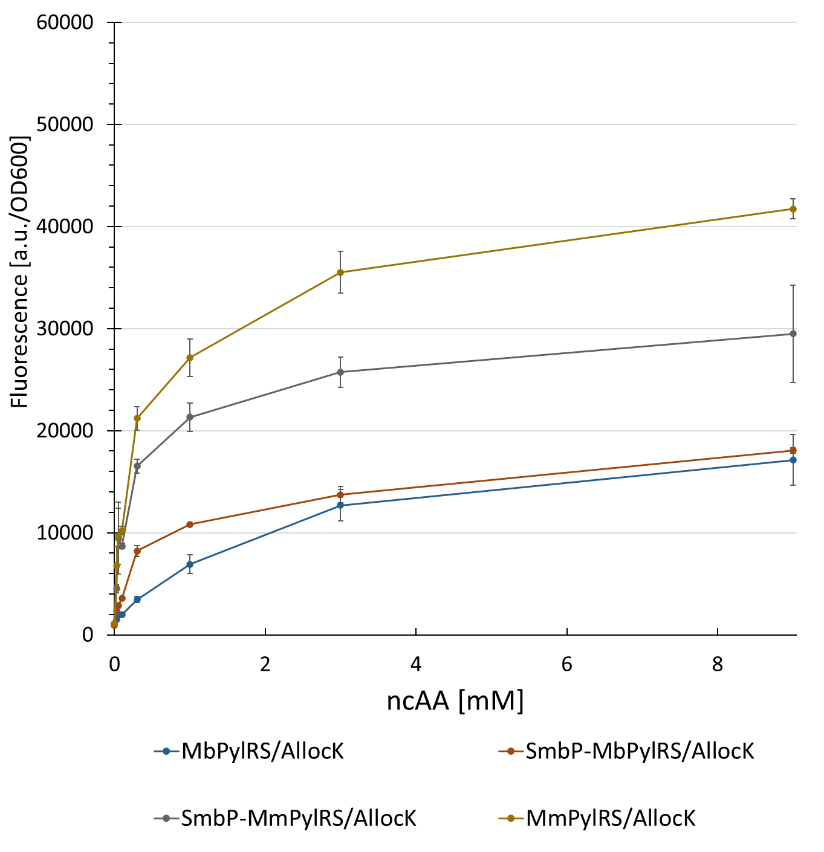


**Figure S26.** Concentration-dependent protein production for four different PylRS variants. Fluorescence intensity of intact *E. coli* BL21(DE3) cells expressing the SUMO-sfGFP(R2 amber) reporter, endpoint measurements after 24 h with different ncAA concentrations (0.025, 0.05, 0.1, 0.3, 1, 3, and 9 mM). Data including standard deviation represents the mean of three biological replicates.


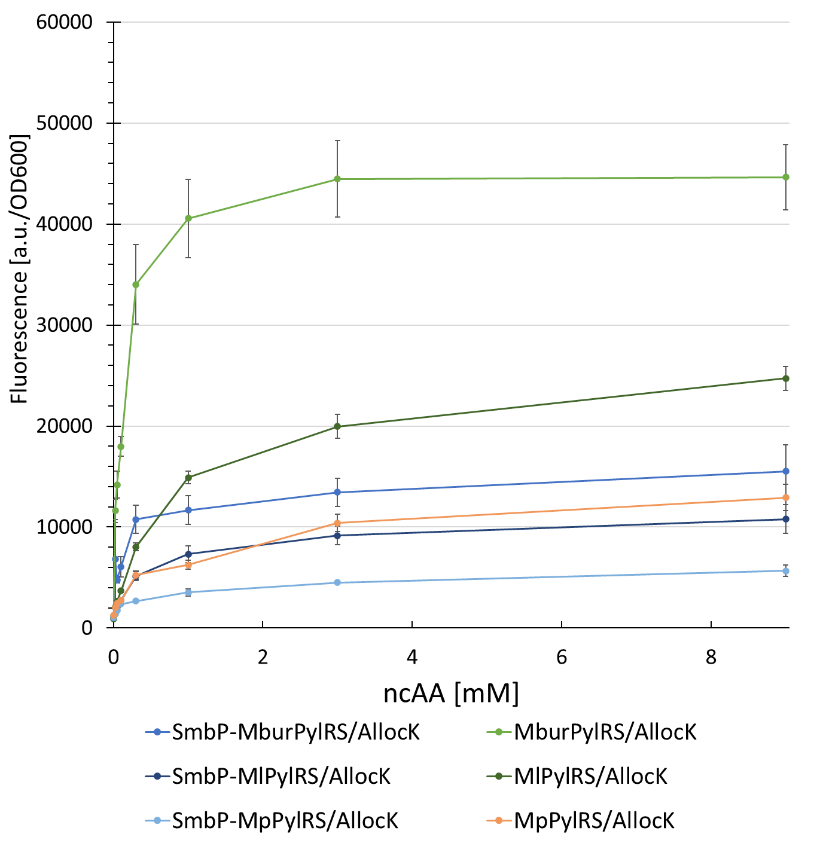


**Figure S27.** Concentration-dependent protein production for six different PylRS variants. Fluorescence intensity of intact *E. coli* BL21(DE3) cells expressing the SUMO-sfGFP(R2 amber) reporter, endpoint measurements after 24 h with different ncAA concentrations (0.025, 0.05, 0.1, 0.3, 1, 3, and 9 mM). The data including the standard deviation represents the mean of three biological replicates.


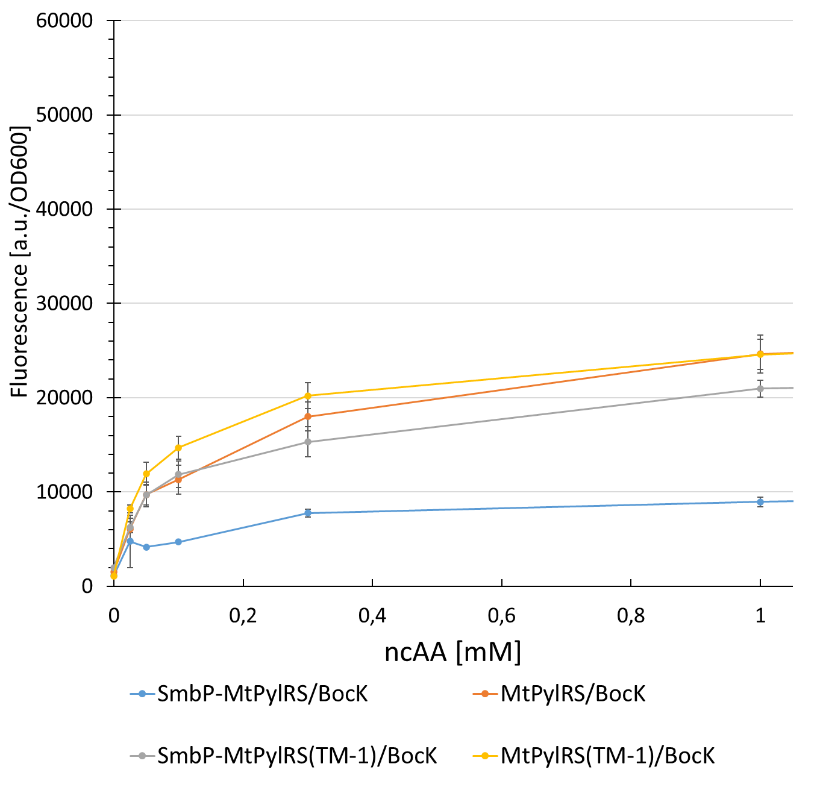


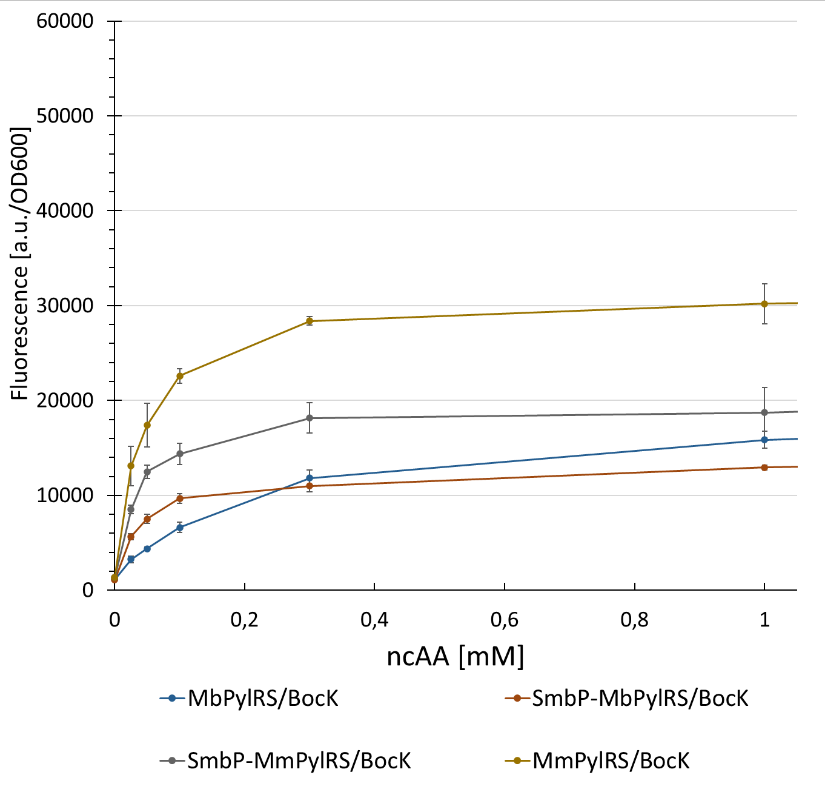
**Figure S28.** Concentration-dependent protein production for four different PylRS variants. Fluorescence intensity of intact *E. coli* BL21(DE3) cells expressing the SUMO-sfGFP(R2 amber) reporter, endpoint measurements after 24 h with different ncAA concentrations (0.025, 0.05, 0.1, 0.3, 1, 3, and 9 mM). Data including standard deviation represents the mean of three biological replicates.

**Figure S29** Concentration-dependent protein production for four different PylRS variants. Fluorescence intensity of intact *E. coli* BL21(DE3) cells expressing the SUMO-sfGFP(R2 amber) reporter, endpoint measurements after 24 h with different ncAA concentrations (0.025, 0.05, 0.1, 0.3, 1, 3, and 9 mM). The data including the standard deviation represents the mean of three biological replicates.


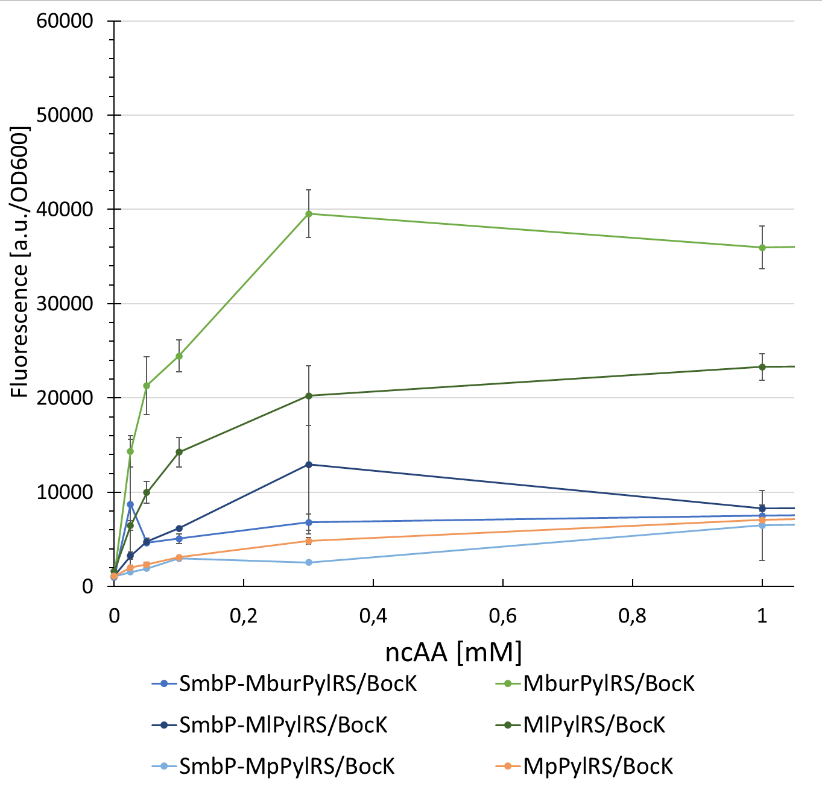


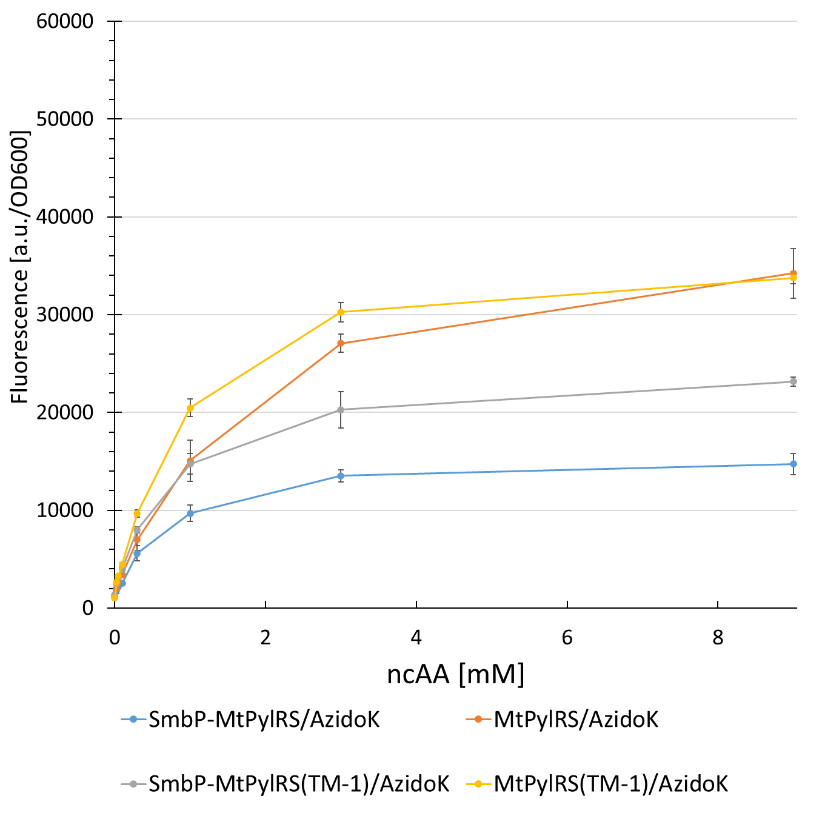
**Figure S30.** Concentration-dependent protein production for six different PylRS variants. Fluorescence intensity of intact *E. coli* BL21(DE3) cells expressing the SUMO-sfGFP(R2 amber) reporter, endpoint measurements after 24 h with different ncAA concentrations (0.025, 0.05, 0.1, 0.3, 1, 3, and 9 mM). Data including standard deviation represents the mean of three biological replicates.

**Figure S31.** Concentration-dependent protein production for four different PylRS variants. Fluorescence intensity of intact *E. coli* BL21(DE3) cells expressing the SUMO-sfGFP(R2 amber) reporter, endpoint measurements after 24 h with different ncAA concentrations (0.025, 0.05, 0.1, 0.3, 1, 3, and 9 mM). Data including standard deviation represents the mean of three biological replicates.


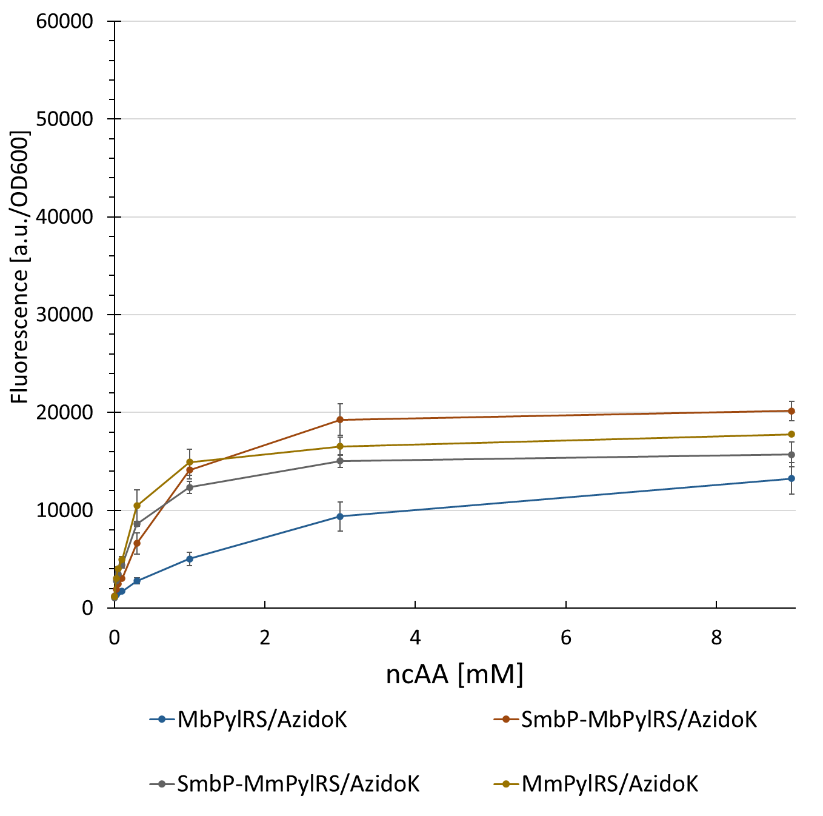


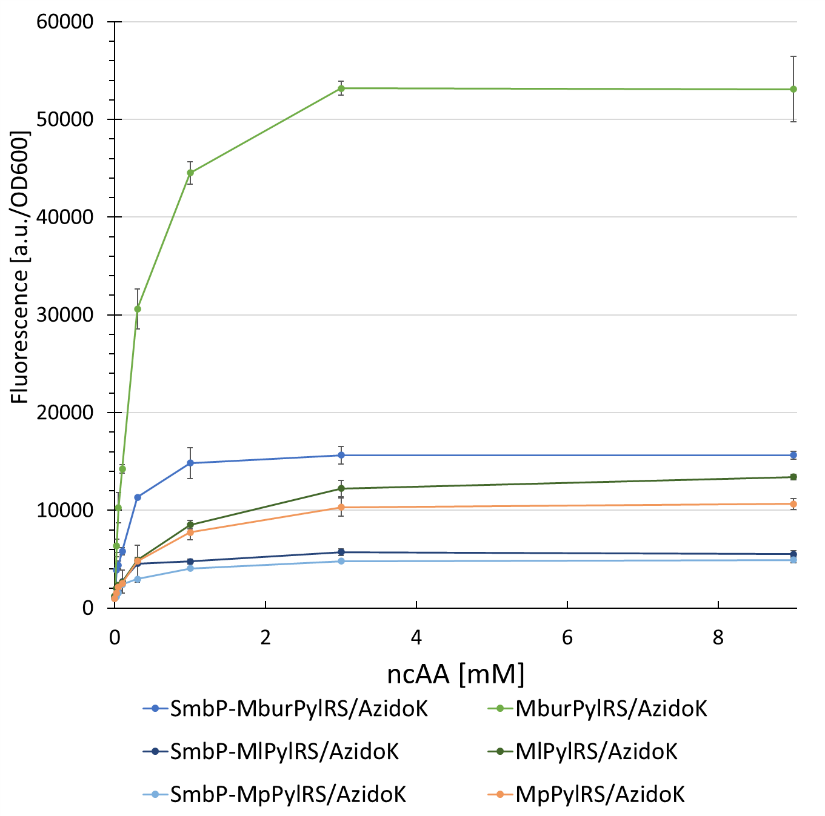
**Figure S32.** Concentration-dependent protein production for four different PylRS variants. Fluorescence intensity of intact *E. coli* BL21(DE3) cells expressing the SUMO-sfGFP(R2 amber) reporter, endpoint measurements after 24 h with different ncAA concentrations (0.025, 0.05, 0.1, 0.3, 1, 3, and 9 mM). Data including standard deviation represents the mean of three biological replicates.

**Figure S33.** Concentration-dependent protein production for six different PylRS variants. Fluorescence intensity of intact *E. coli* BL21(DE3) cells expressing the SUMO-sfGFP(R2 amber) reporter, endpoint measurements after 24 h with different ncAA concentrations (0.025, 0.05, 0.1, 0.3, 1, 3, and 9 mM). Data including standard deviation represents the mean of three biological replicates.


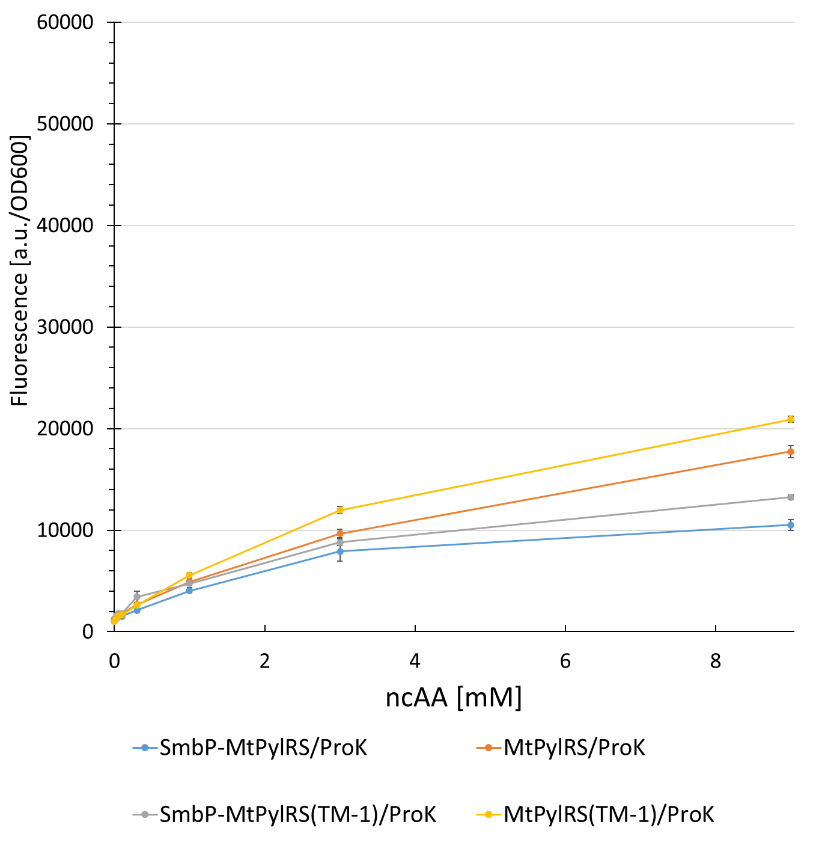


**Figure S34.** Concentration-dependent protein production for four different PylRS variants. Fluorescence intensity of intact *E. coli* BL21(DE3) cells expressing the SUMO-sfGFP(R2 amber) reporter, endpoint measurements after 24 h with different ncAA concentrations (0.025, 0.05, 0.1, 0.3, 1, 3, and 9 mM). Data including standard deviation represents the mean of three biological replicates.


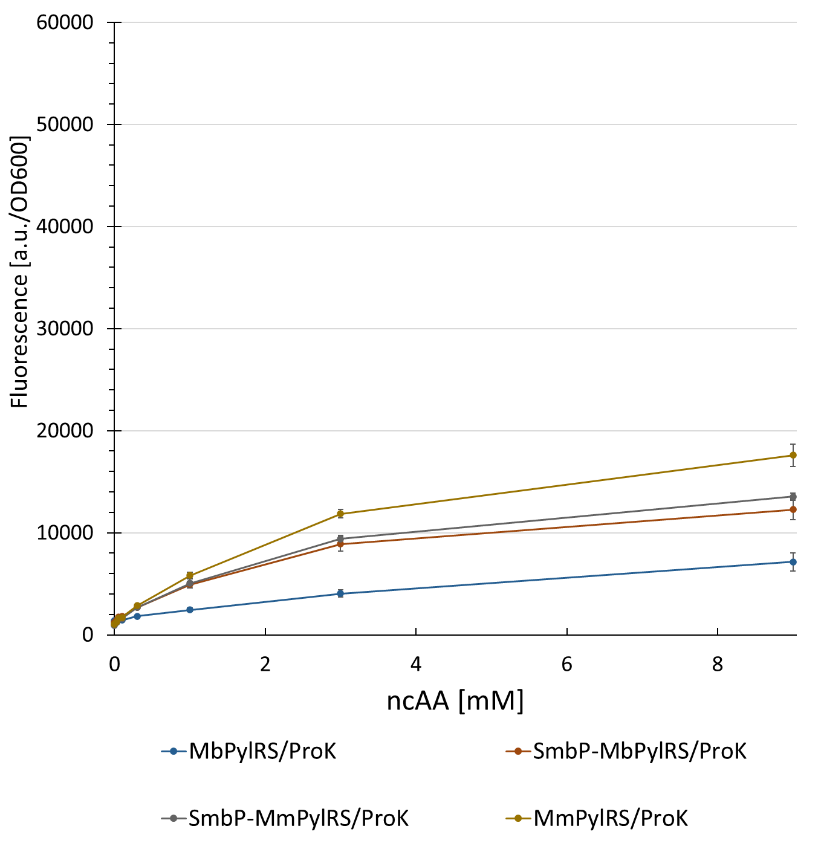


**Figure S35.** Concentration-dependent protein production for four different PylRS variants. Fluorescence intensity of intact *E. coli* BL21(DE3) cells expressing the SUMO-sfGFP(R2 amber) reporter, endpoint measurements after 24 h with different ncAA concentrations (0.025, 0.05, 0.1, 0.3, 1, 3, and 9 mM). Data including standard deviation represents the mean of three biological replicates.


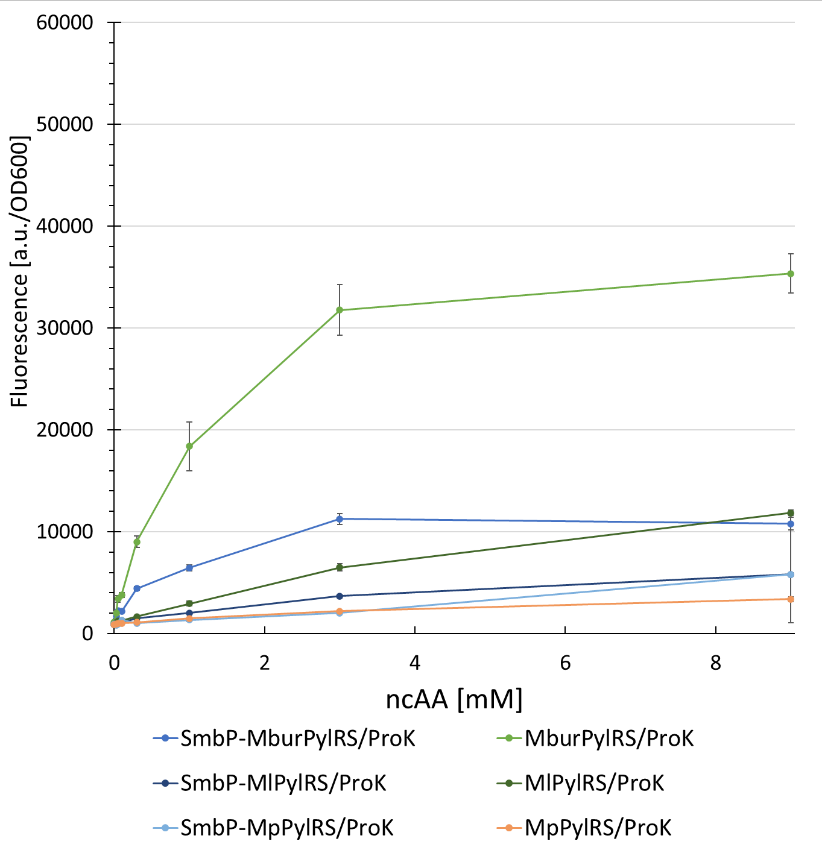


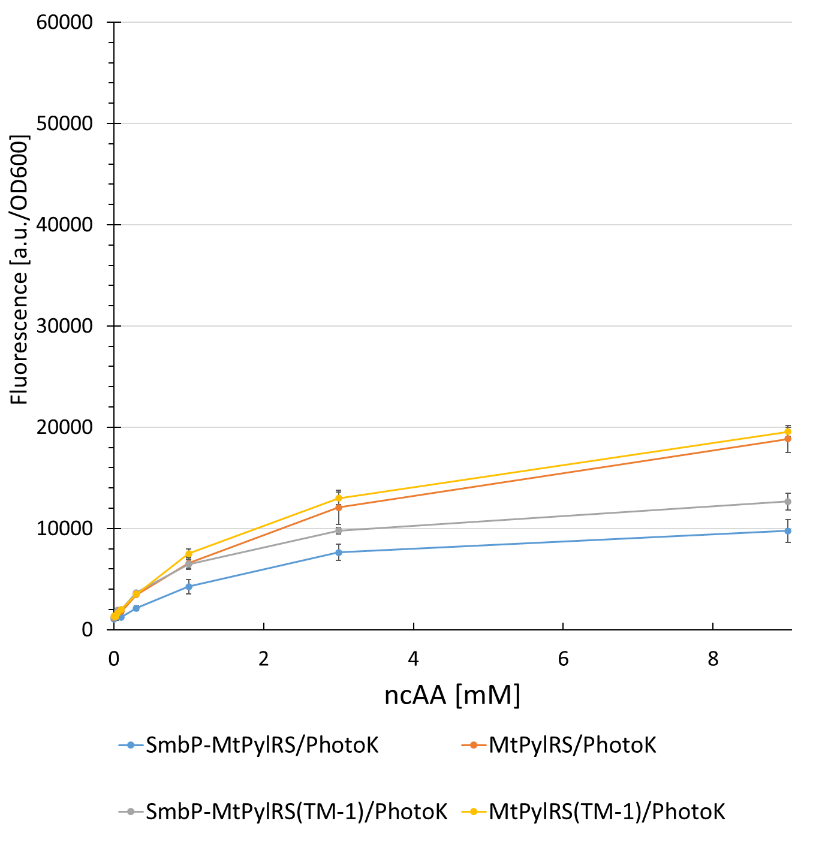
**Figure S36.** Concentration-dependent protein production for six different PylRS variants. Fluorescence intensity of intact *E. coli* BL21(DE3) cells expressing the SUMO-sfGFP(R2 amber) reporter, endpoint measurements after 24 h with different ncAA concentrations (0.025, 0.05, 0.1, 0.3, 1, 3, and 9 mM). Data including standard deviation represents the mean of three biological replicates.

**Figure S37.** Concentration-dependent protein production for four different PylRS variants. Fluorescence intensity of intact *E. coli* BL21(DE3) cells expressing the SUMO-sfGFP(R2 amber) reporter, endpoint measurements after 24 h with different ncAA concentrations (0.025, 0.05, 0.1, 0.3, 1, 3, and 9 mM). TData including standard deviation represents the mean of three biological replicates.


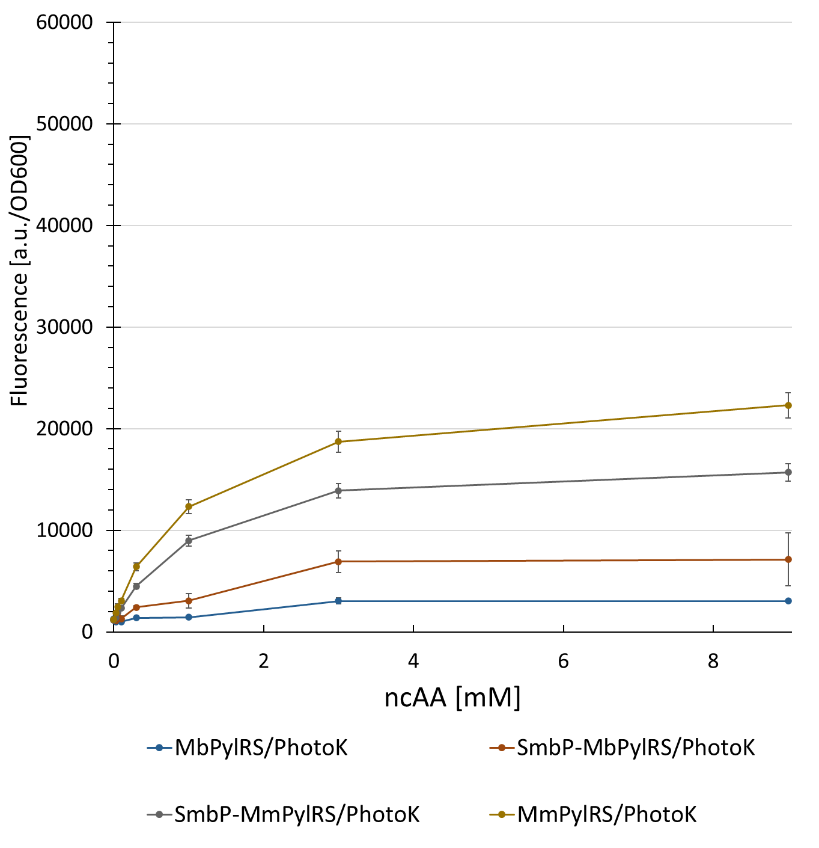


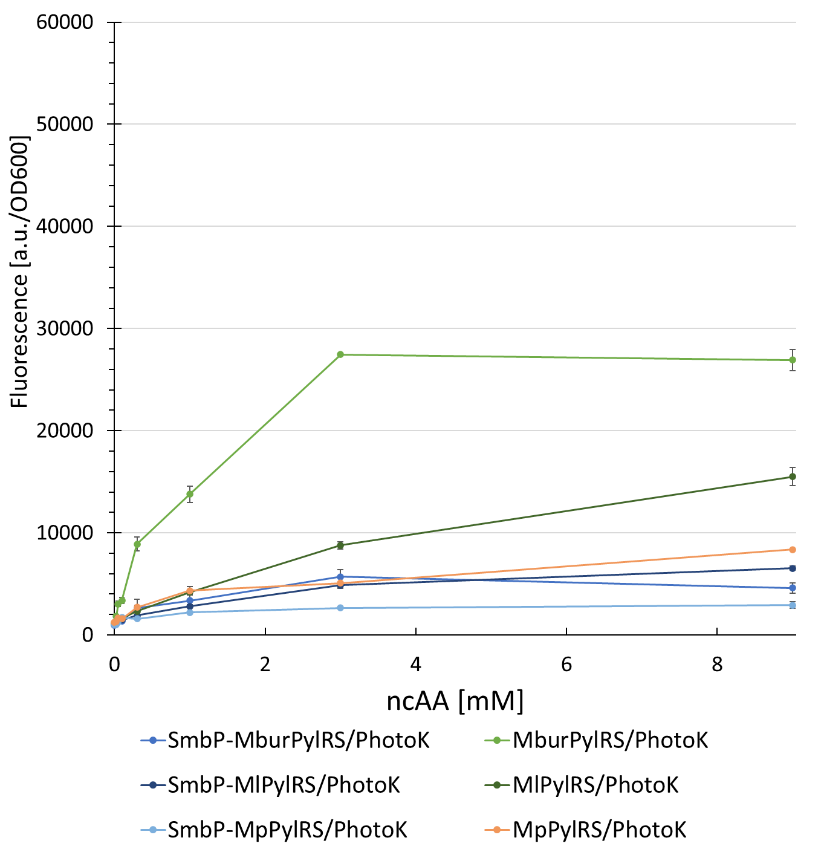
**Figure S38.** Concentration-dependent protein production for four different PylRS variants. Fluorescence intensity of intact *E. coli* BL21(DE3) cells expressing the SUMO-sfGFP(R2 amber) reporter, endpoint measurements after 24 h with different ncAA concentrations (0.025, 0.05, 0.1, 0.3, 1, 3, and 9 mM). Data including standard deviation represents the mean of three biological replicates.

**Figure S39.** Concentration-dependent protein production for six different PylRS variants. Fluorescence intensity of intact *E. coli* BL21(DE3) cells expressing the SUMO-sfGFP(R2 amber) reporter, endpoint measurements after 24 h with different ncAA concentrations (0.025, 0.05, 0.1, 0.3, 1, 3, and 9 mM). Data including standard deviation represents the mean of three biological replicates.


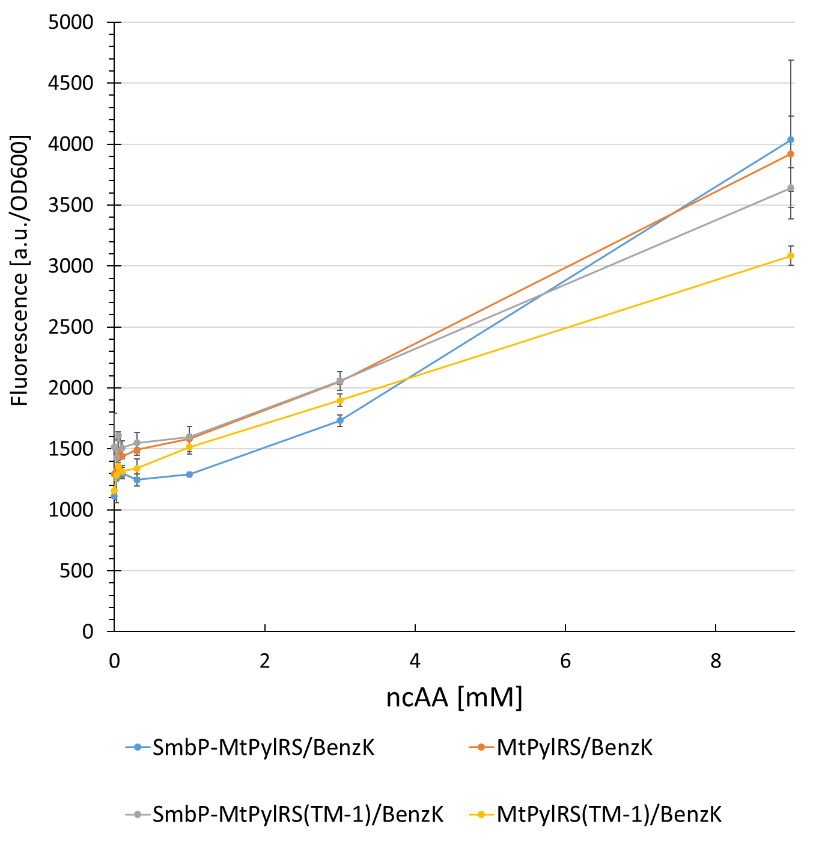


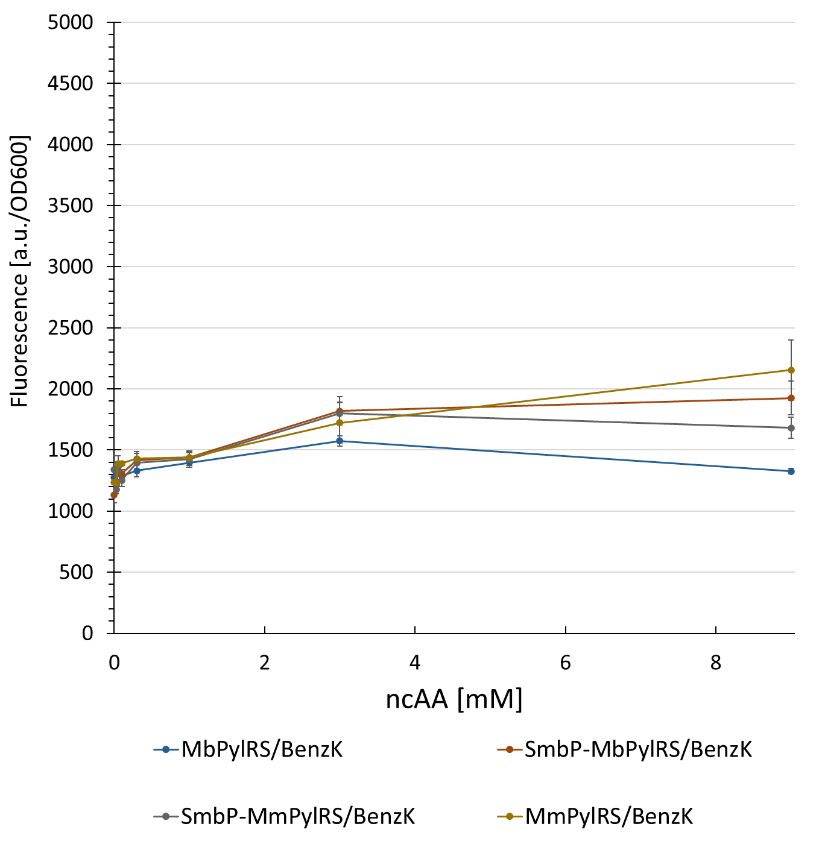
**Figure S40.** Concentration-dependent protein production for four different PylRS variants. Fluorescence intensity of intact *E. coli* BL21(DE3) cells expressing the SUMO-sfGFP(R2 amber) reporter, endpoint measurements after 24 h with different ncAA concentrations (0.025, 0.05, 0.1, 0.3, 1, 3, and 9 mM). Data including standard deviation represents the mean of three biological replicates.

**Figure S41.** Concentration-dependent protein production for four different PylRS variants. Fluorescence intensity of intact *E. coli* BL21(DE3) cells expressing the SUMO-sfGFP(R2 amber) reporter, endpoint measurements after 24 h with different ncAA concentrations (0.025, 0.05, 0.1, 0.3, 1, 3, and 9 mM). Data including standard deviation represents the mean of three biological replicates.


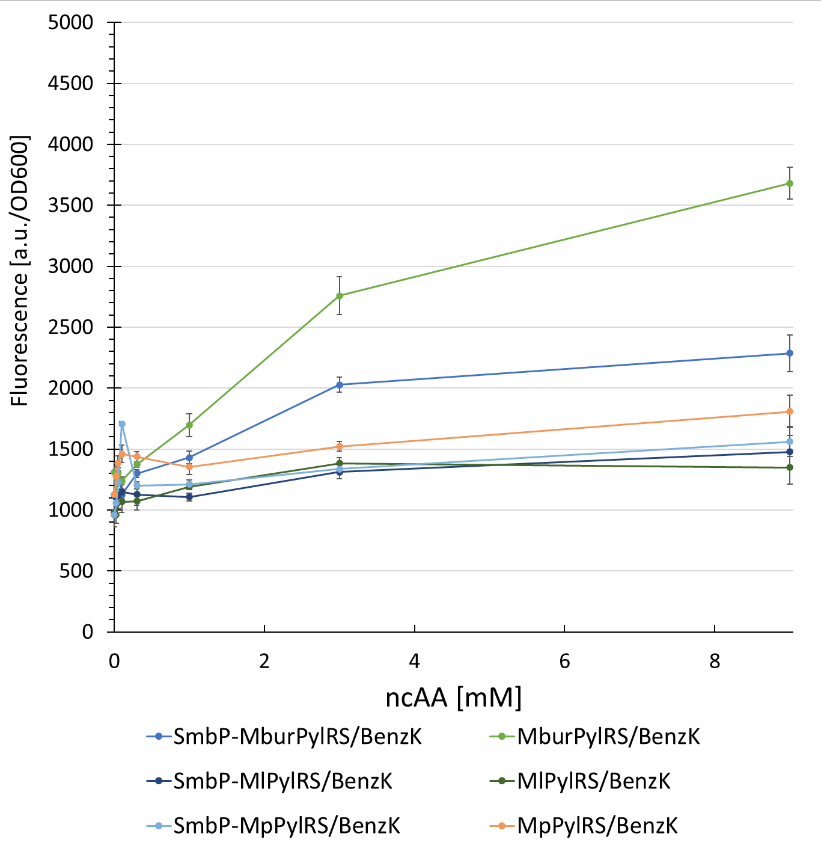


**Figure S42.** Concentration-dependent protein production for six different PylRS variants. Fluorescence intensity of intact *E. coli* BL21(DE3) cells expressing the SUMO-sfGFP(R2 amber) reporter, endpoint measurements after 24 h with different ncAA concentrations (0.025, 0.05, 0.1, 0.3, 1, 3, and 9 mM). Data including standard deviation represents the mean of three biological replicates.


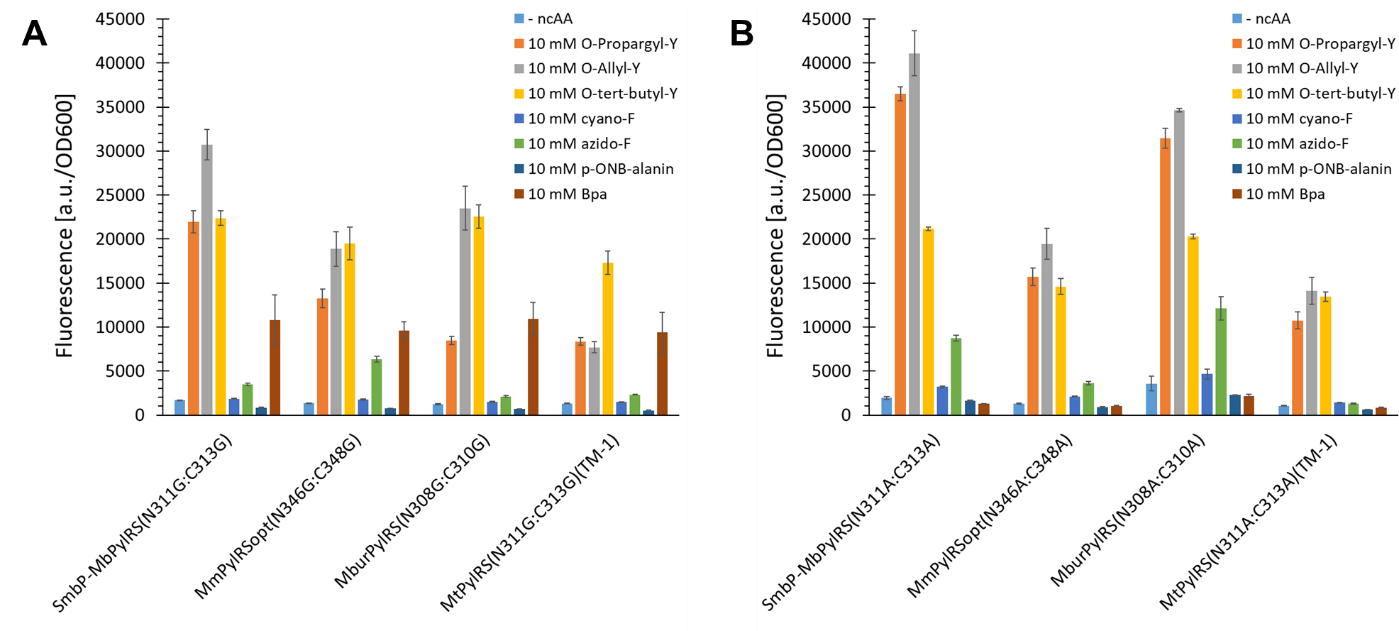


**Figure S43.** Comparison of ncAA (**39**, **40**, **44**, **45**, **46**, **49**, **50**) incorporation efficiency for PylRS double Ala and double Gly mutants. Fluorescence measurement of intact *E. coli* BL21(DE3) cells producing the SUMO-sfGFP(R2amber) reporter protein. Endpoint measurements after 24 h of incubation. Data including standard deviation represents the mean of three biological replicates. 10 mM ncAAs supplied.


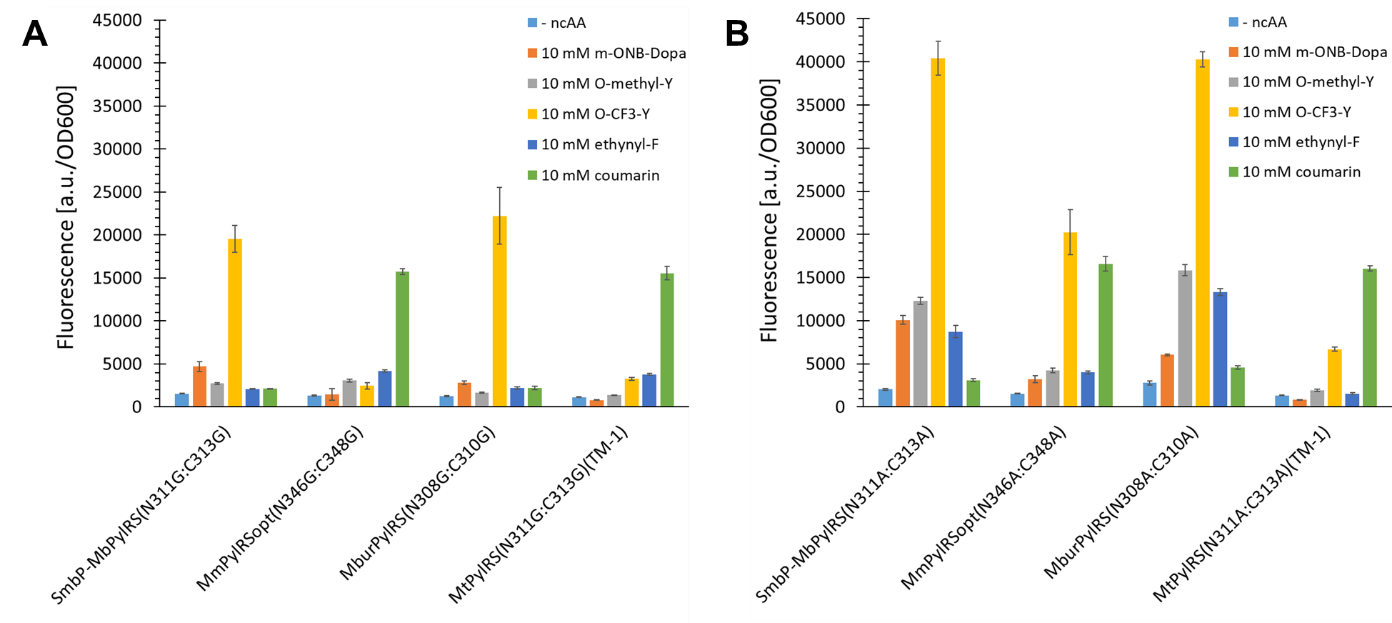


**Figure S44.** Comparison of ncAA (**38**, **47**, **48**, **51**, **52**) incorporation efficiency for PylRS double Ala and double Gly mutants. Fluorescence measurement of intact *E. coli* BL21(DE3) cells producing the SUMO-sfGFP(R2amber) reporter protein. Endpoint measurements after 24 h of incubation. Data including standard deviation represents the mean of three biological replicates. 10 mM ncAAs supplied.


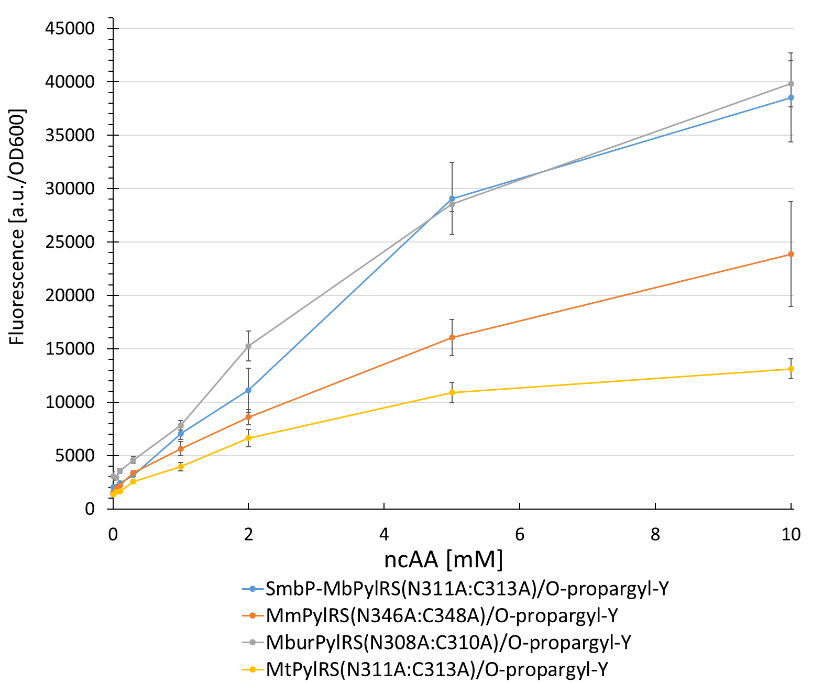


**Figure S45.** Concentration-dependent protein production for four different PylRS double Ala mutants. Fluorescence intensity of intact *E. coli* BL21(DE3) cells expressing the SUMO-sfGFP(R2 amber) reporter. Endpoint measurements after 24 h with different ncAA concentrations (0.5, 0.1, 0.3, 1, 2, 3, and 10 mM). Data including standard deviation represents the mean of three biological replicates.


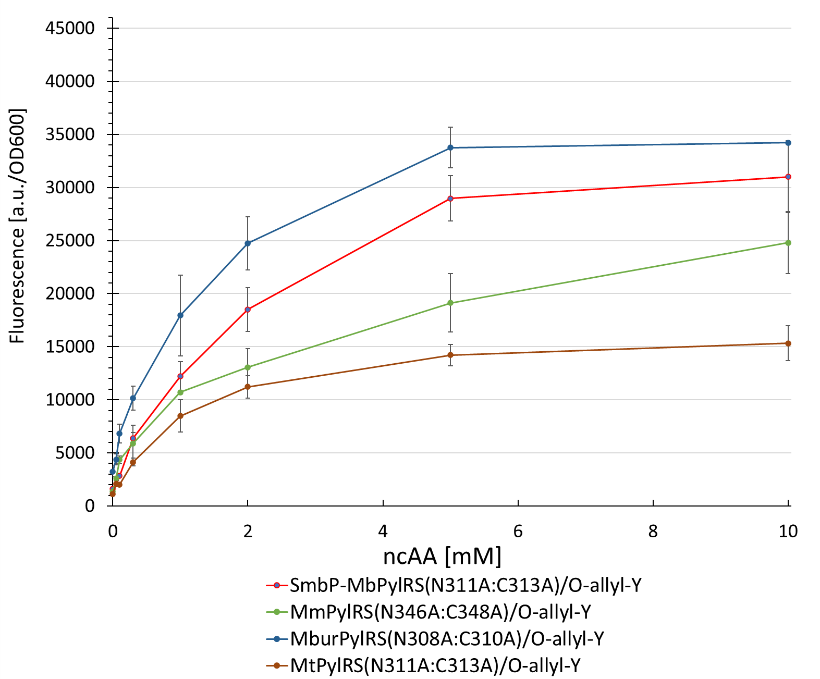


**Figure S46.** Concentration-dependent protein production for four different PylRS double Ala mutants. Fluorescence intensity of intact *E. coli* BL21(DE3) cells expressing the SUMO-sfGFP(R2 amber) reporter. Endpoint measurements after 24 h with different ncAA concentrations (0.5, 0.1, 0.3, 1, 2, 3, and 10 mM). Data including standard deviation represents the mean of three biological replicates.


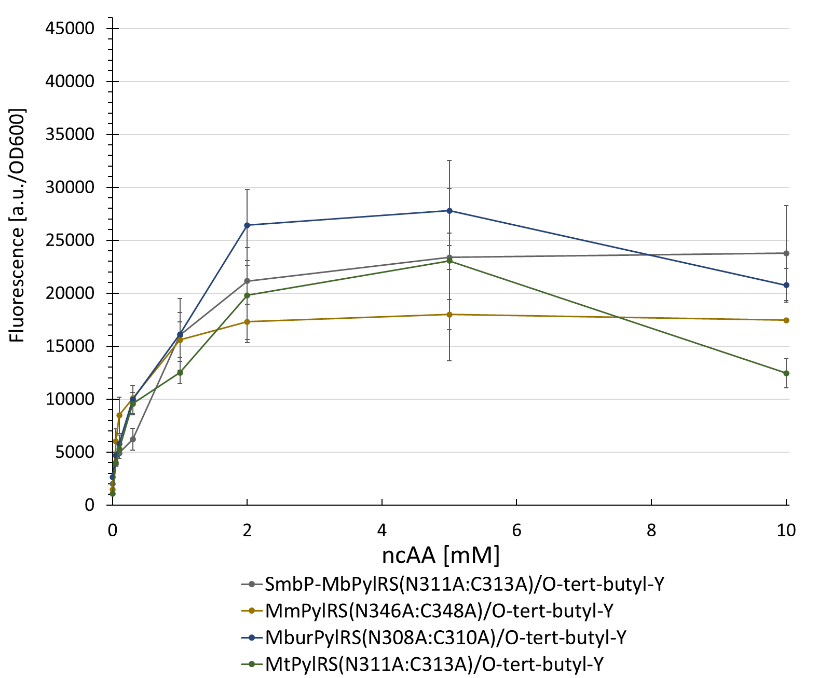


**Figure S47.** Concentration-dependent protein production for four different PylRS double Ala mutants. Fluorescence intensity of intact *E. coli* BL21(DE3) cells expressing the SUMO-sfGFP(R2 amber) reporter. Endpoint measurements after 24 h with different ncAA concentrations (0.5, 0.1, 0.3, 1, 2, 3, and 10 mM). Data including standard deviation represents the mean of three biological replicates.


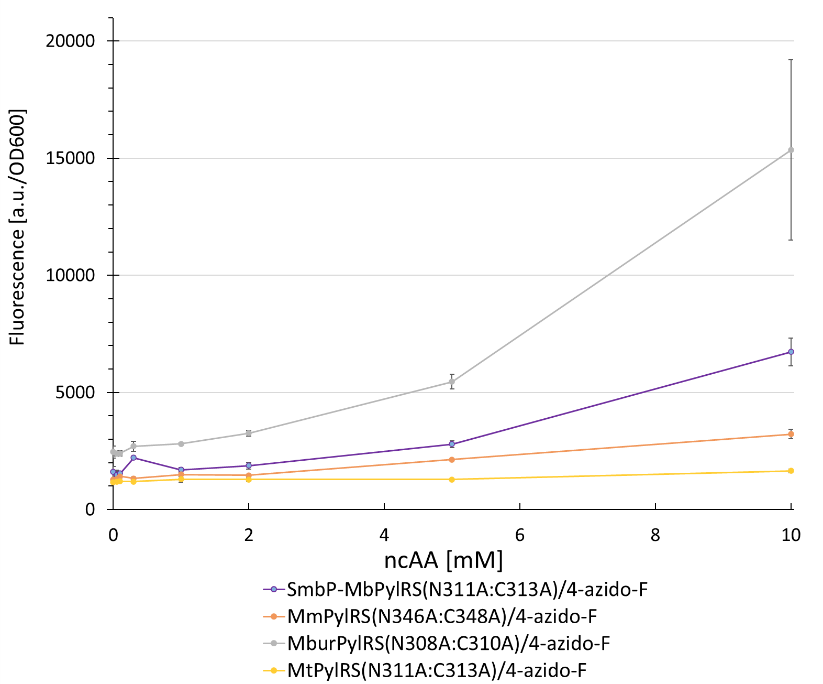


**Figure S48.** Concentration-dependent protein production for four different PylRS double Ala mutants. Fluorescence intensity of intact *E. coli* BL21(DE3) cells expressing the SUMO-sfGFP(R2 amber) reporter. Endpoint measurements after 24 h with different ncAA concentrations (0.5, 0.1, 0.3, 1, 2, 3, and 10 mM). Data including standard deviation represents the mean of three biological replicates.


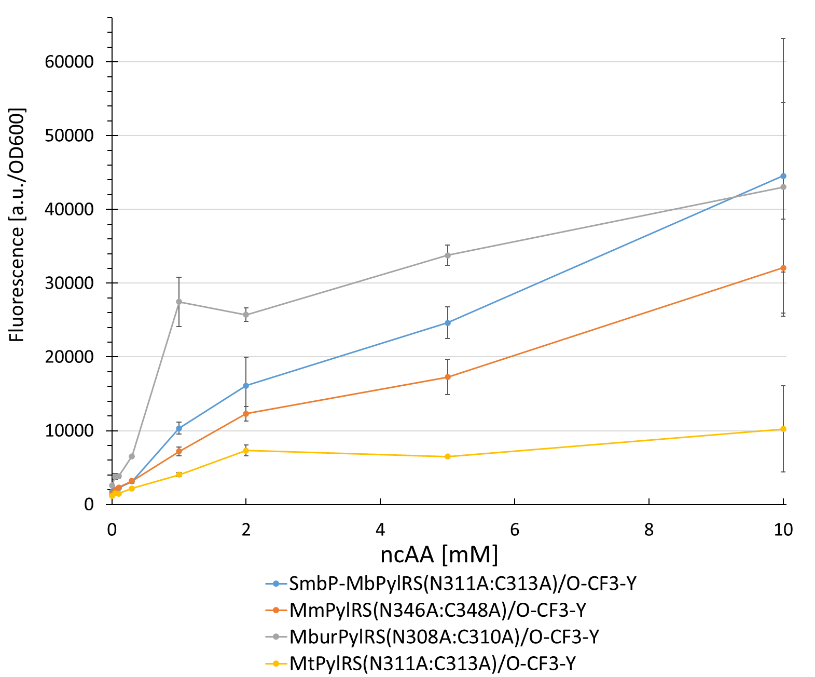


**Figure S49.** Concentration-dependent protein production for four different PylRS double Ala mutants. Fluorescence intensity of intact *E. coli* BL21(DE3) cells expressing the SUMO-sfGFP(R2 amber) reporter. Endpoint measurements after 24 h with different ncAA concentrations (0.5, 0.1, 0.3, 1, 2, 3, and 10 mM). Data including standard deviation represents the mean of three biological replicates.


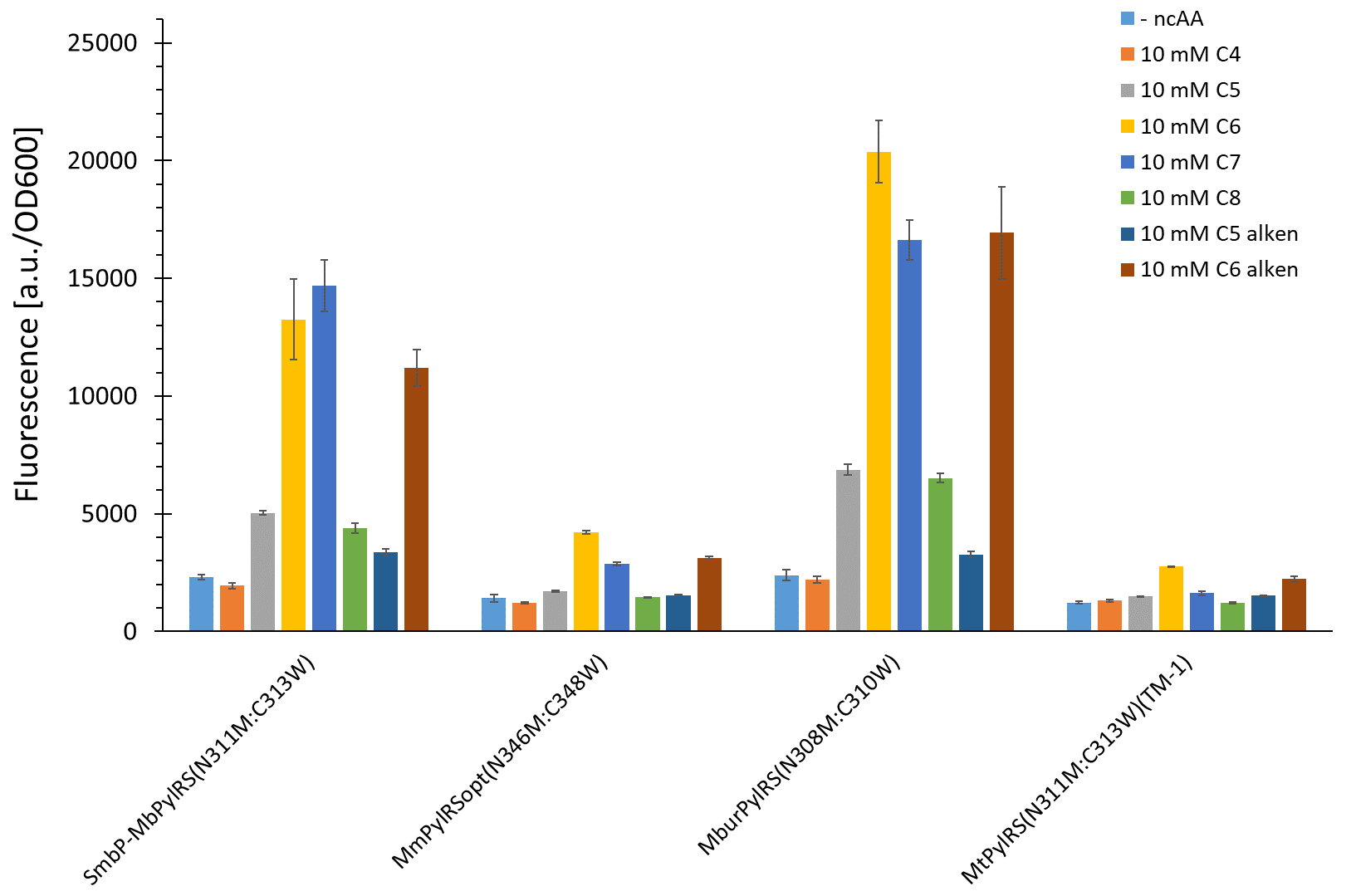


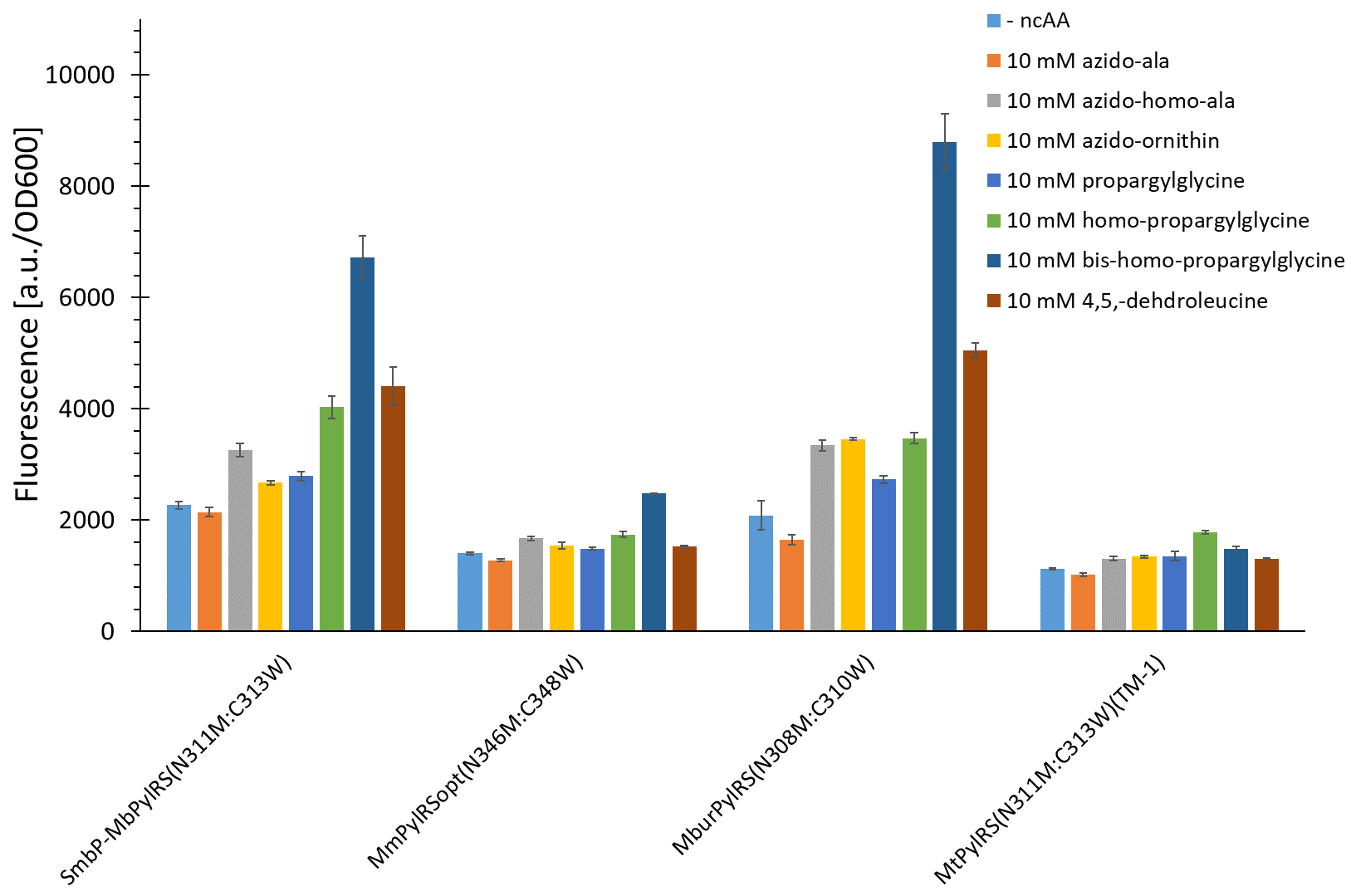
**Figure S50.** Comparison of ncAA (**10**, **7**, **5**, **2**, **3**, **8**, **6**) incorporation efficiency for different PylRS constructs. Fluorescence measurement of intact *E. coli* BL21(DE3) cells producing the SUMO-sfGFP(R2amber) reporter protein. Endpoint measurements after 24 h of incubation. Data including standard deviation represents the mean of three biological replicates. 10 mM ncAAs supplied

**Figure S51.** Comparison of ncAA (**14**, **15**, **16**, **13**, **12**, **11**, **21**) incorporation efficiency for different PylRS constructs. Fluorescence measurement of intact *E. coli* BL21(DE3) cells producing the SUMO-sfGFP(R2amber) reporter protein. Endpoint measurements after 24 h of incubation. Data including standard deviation represents the mean of three biological replicates. 10 mM ncAAs supplied


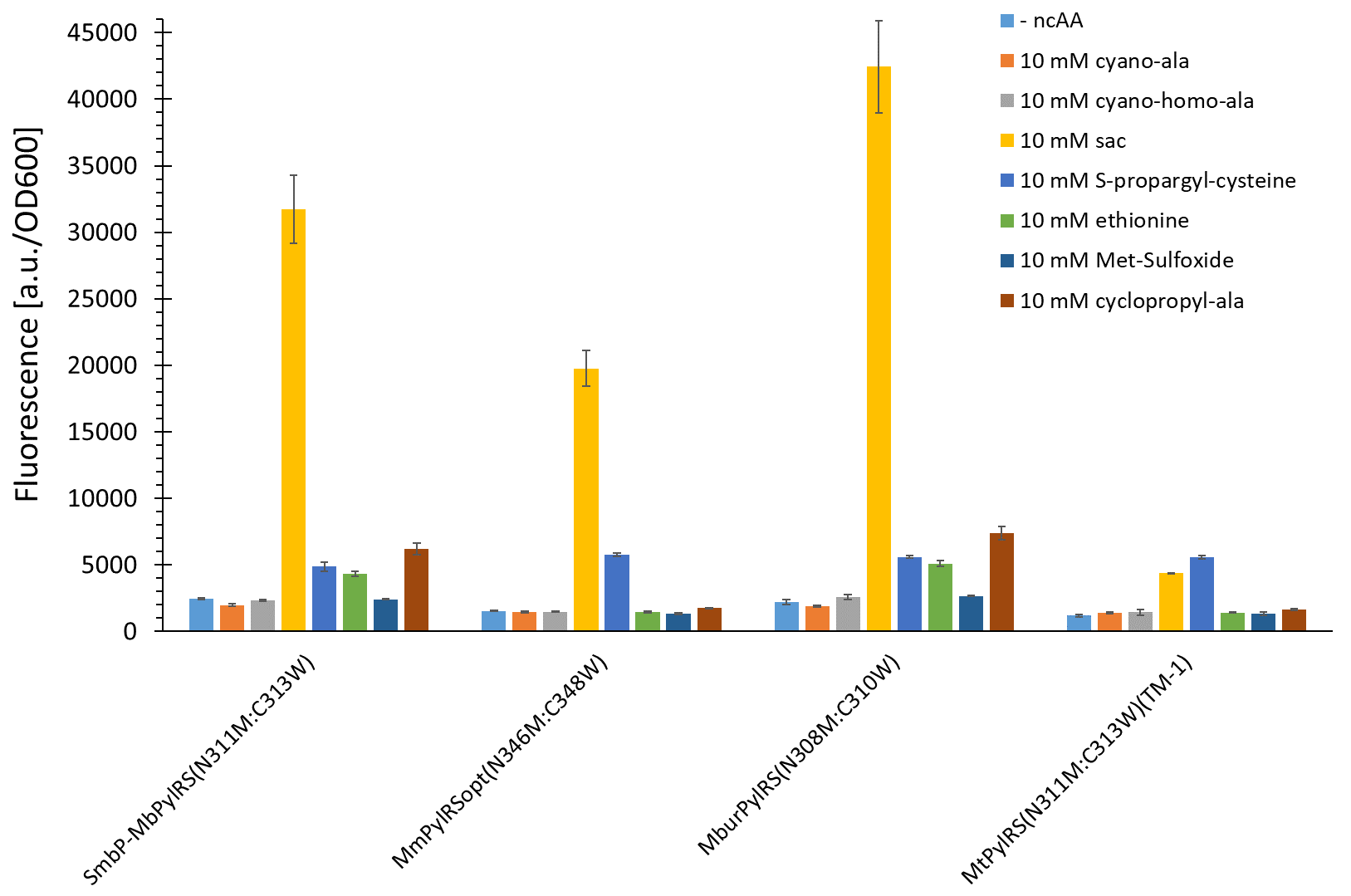


**Figure S52.** Comparison of ncAA (**18**, **19**, **1**, **27**, **25**, **23**, **9**) incorporation efficiency for different PylRS constructs. Fluorescence measurement of intact *E. coli* BL21(DE3) cells producing the SUMO-sfGFP(R2amber) reporter protein. Endpoint measurements after 24 h of incubation. Data including standard deviation represents the mean of three biological replicates. 10 mM ncAAs supplied


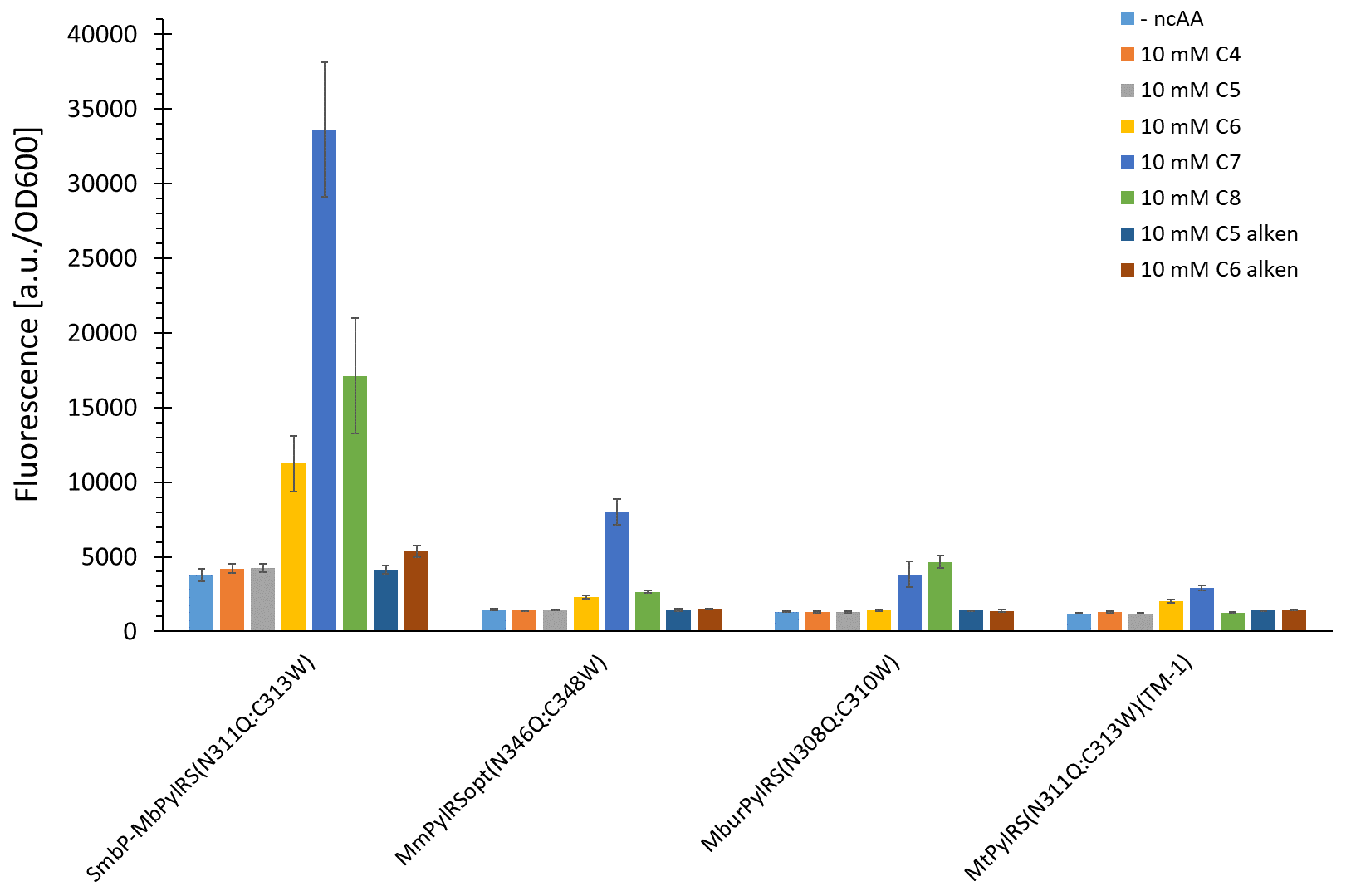


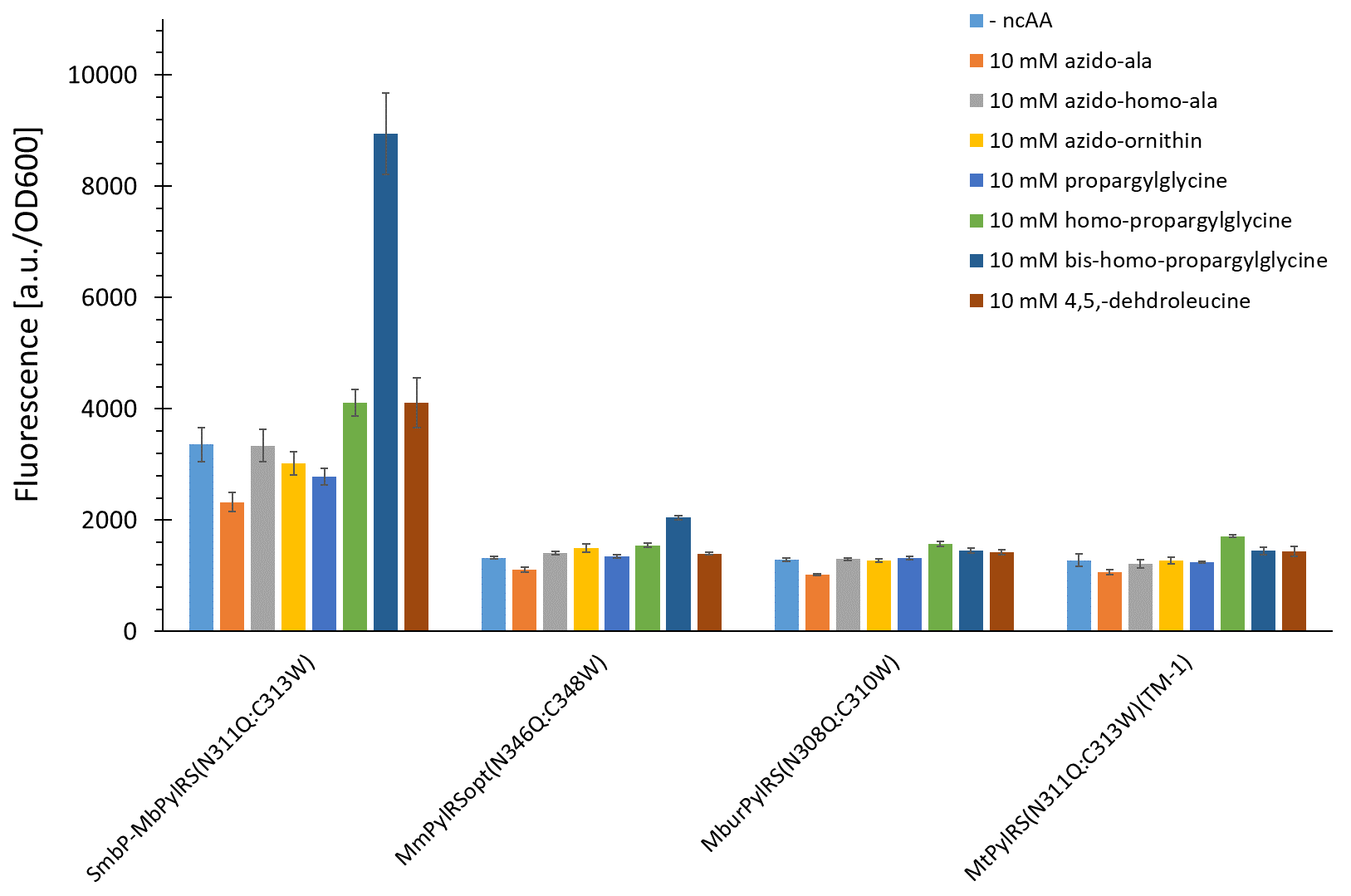
**Figure S53.** Comparison of ncAA (**10**, **7**, **5**, **2**, **3**, **8**, **6**) incorporation efficiency for different PylRS constructs. Fluorescence measurement of intact *E. coli* BL21(DE3) cells producing the SUMO-sfGFP(R2amber) reporter protein. Endpoint measurements after 24 h of incubation. Data including standard deviation represents the mean of three biological replicates. 10 mM ncAAs supplied

**Figure S54.** Comparison of ncAA (**14**, **15**, **16**, **13**, **12**, **11**, **21**) incorporation efficiency for different PylRS constructs. Fluorescence measurement of intact *E. coli* BL21(DE3) cells producing the SUMO-sfGFP(R2amber) reporter protein. Endpoint measurements after 24 h of incubation. Data including standard deviation represents the mean of three biological replicates. 10 mM ncAAs supplied.


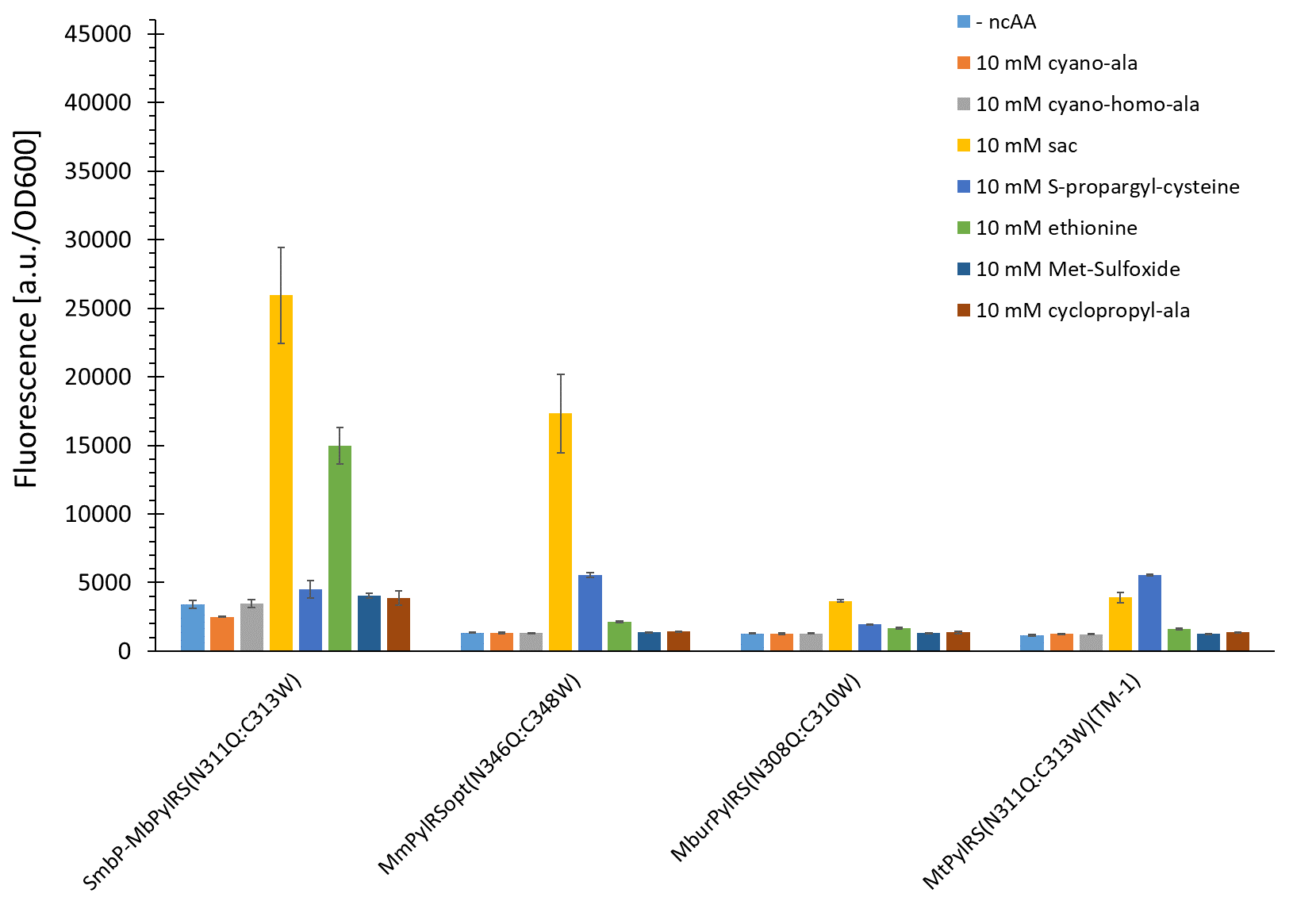


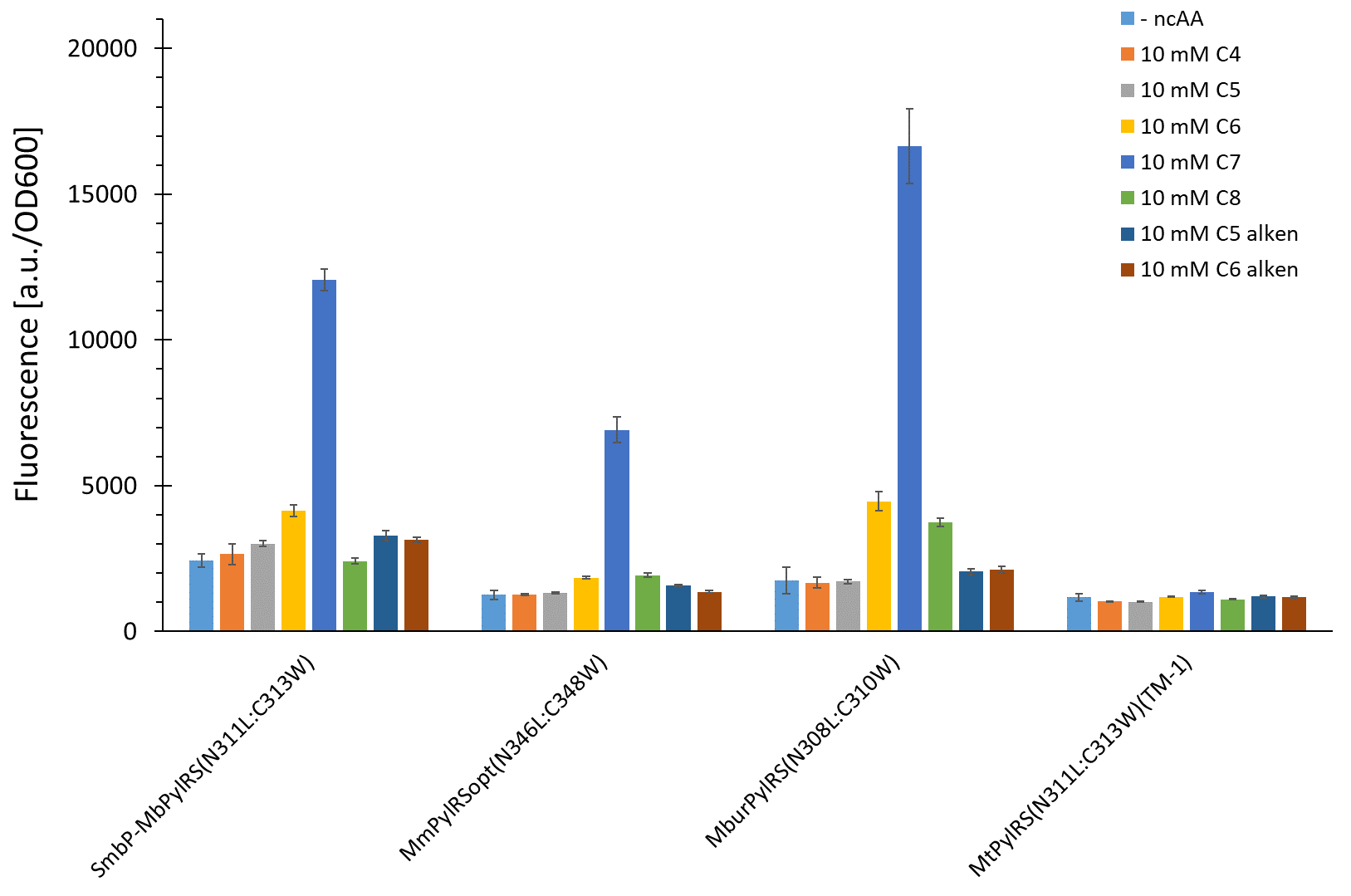
**Figure S55.** Comparison of ncAA (**18**, **19**, **1**, **27**, **25**, **23**, **9**) incorporation efficiency for different PylRS constructs. Fluorescence measurement of intact *E. coli* BL21(DE3) cells producing the SUMO-sfGFP(R2amber) reporter protein. Endpoint measurements after 24 h of incubation. Data including standard deviation represents the mean of three biological replicates. 10 mM ncAAs supplied.

**Figure S56.** Comparison of ncAA (**10**, **7**, **5**, **2**, **3**, **8**, **6**) incorporation efficiency for different PylRS constructs. Fluorescence measurement of intact *E. coli* BL21(DE3) cells producing the SUMO-sfGFP(R2amber) reporter protein. Endpoint measurements after 24 h of incubation. Data including standard deviation represents the mean of three biological replicates. 10 mM ncAAs supplied


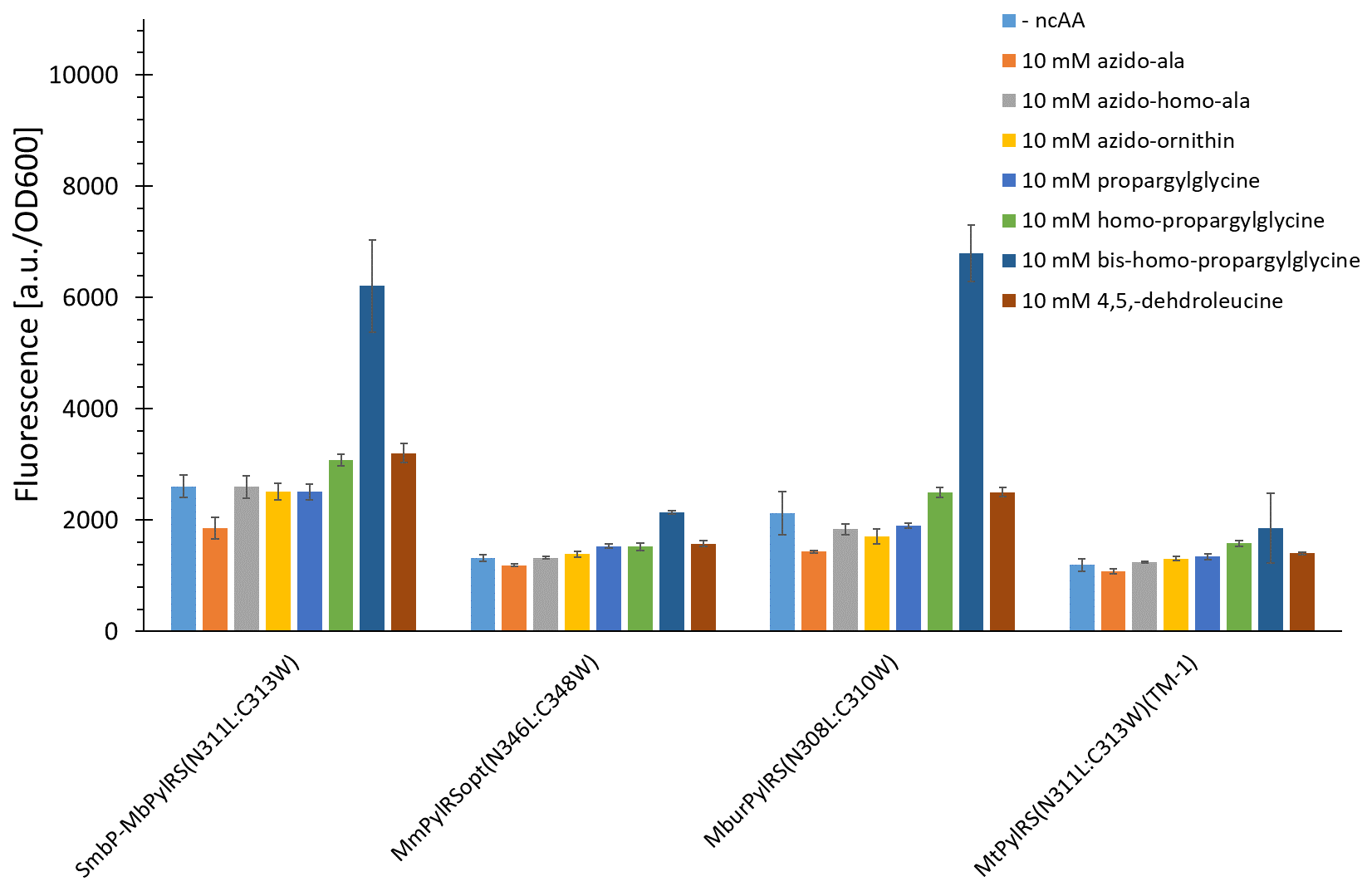


**Figure S57.** Comparison of ncAA (**14**, **15**, **16**, **13**, **12**, **11**, **21**) incorporation efficiency for different PylRS constructs. Fluorescence measurement of intact *E. coli* BL21(DE3) cells producing the SUMO-sfGFP(R2amber) reporter protein. Endpoint measurements after 24 h of incubation. Data including standard deviation represents the mean of three biological replicates. 10 mM ncAAs supplied.


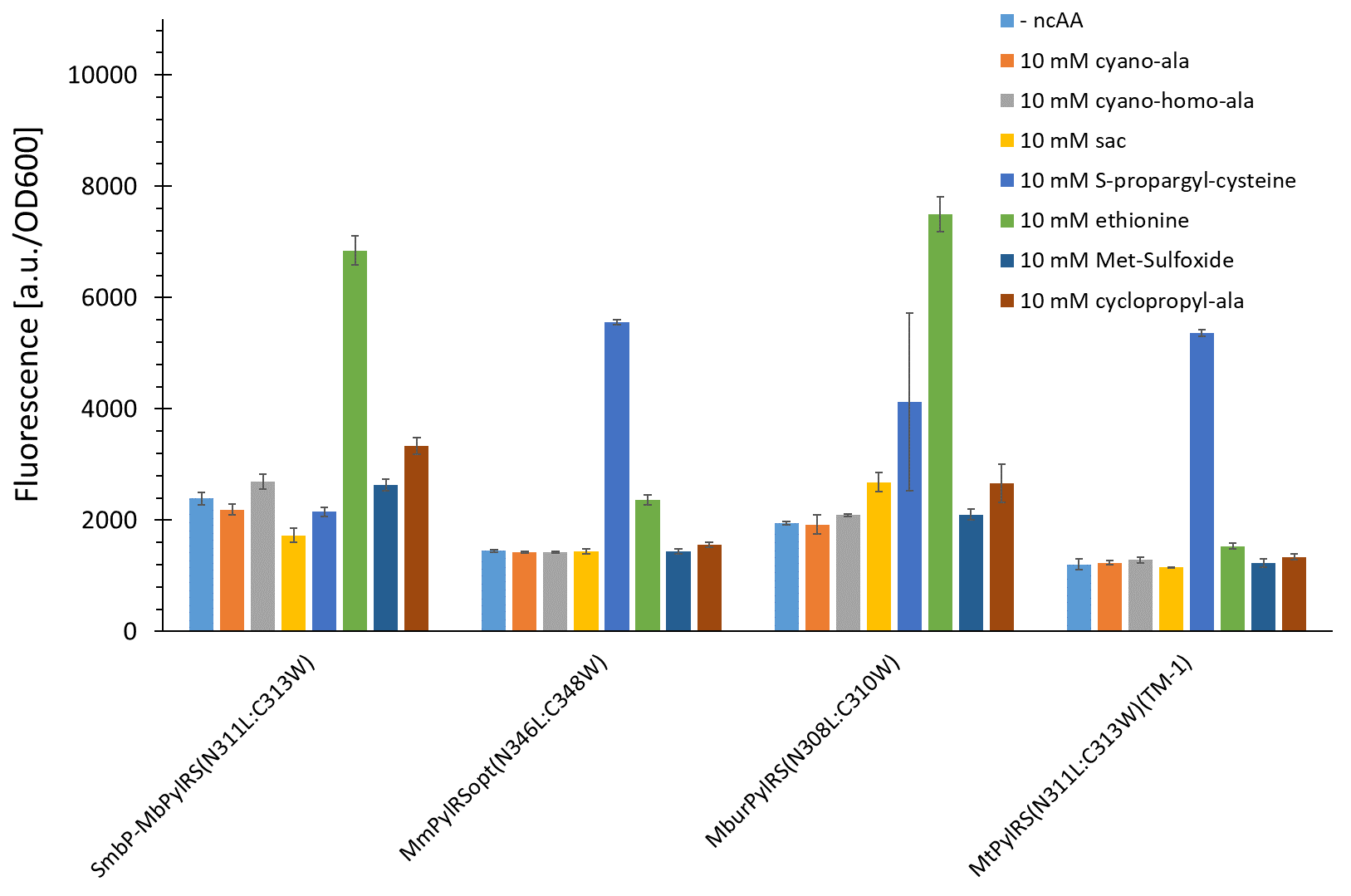


**Figure S58.** Comparison of ncAA (**18**, **19**, **1**, **27**, **25**, **23**, **9**) incorporation efficiency for different PylRS constructs. Fluorescence measurement of intact *E. coli* BL21(DE3) cells producing the SUMO-sfGFP(R2amber) reporter protein. Endpoint measurements after 24 h of incubation. Data including standard deviation represents the mean of three biological replicates. 10 mM ncAAs supplied


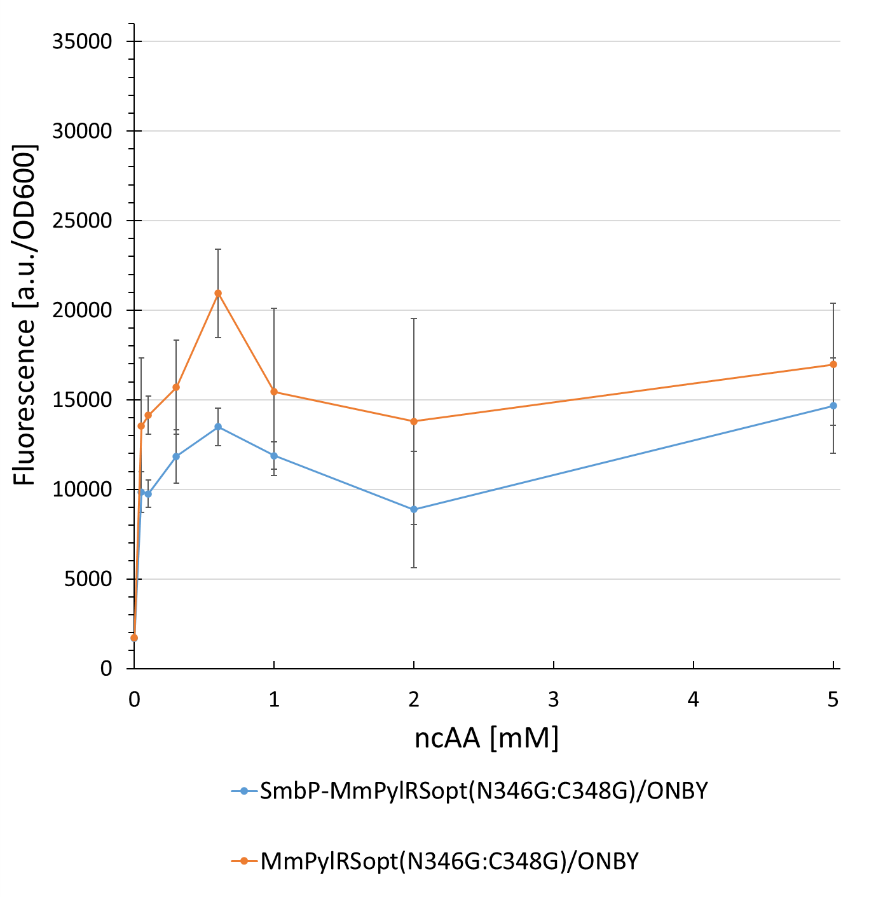


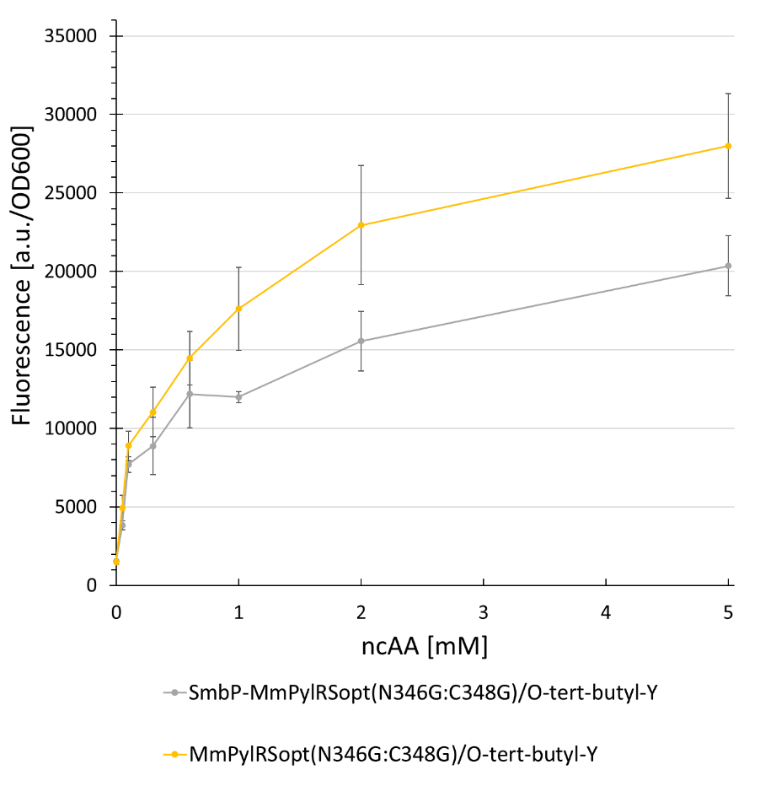
**Figure S59.** Concentration-dependent protein production for two different PylRS double Gly mutants. Fluorescence intensity of intact *E. coli* BL21(DE3) cells expressing the SUMO-sfGFP(R2 amber) reporter. Endpoint measurements after 24 h with different ncAA concentrations (0.05, 0.1, 0.3, 0.6, 1, 2, and 5 mM). Data including standard deviation represents the mean of three biological replicates.

**Figure S60.** Concentration-dependent protein production for two different PylRS double Gly mutants. Fluorescence intensity of intact *E. coli* BL21(DE3) cells expressing the SUMO-sfGFP(R2 amber) reporter. Endpoint measurements after 24 h with different ncAA concentrations (0.05, 0.1, 0.3, 0.6, 1, 2, and 5 mM). Data including standard deviation represents the mean of three biological replicates.


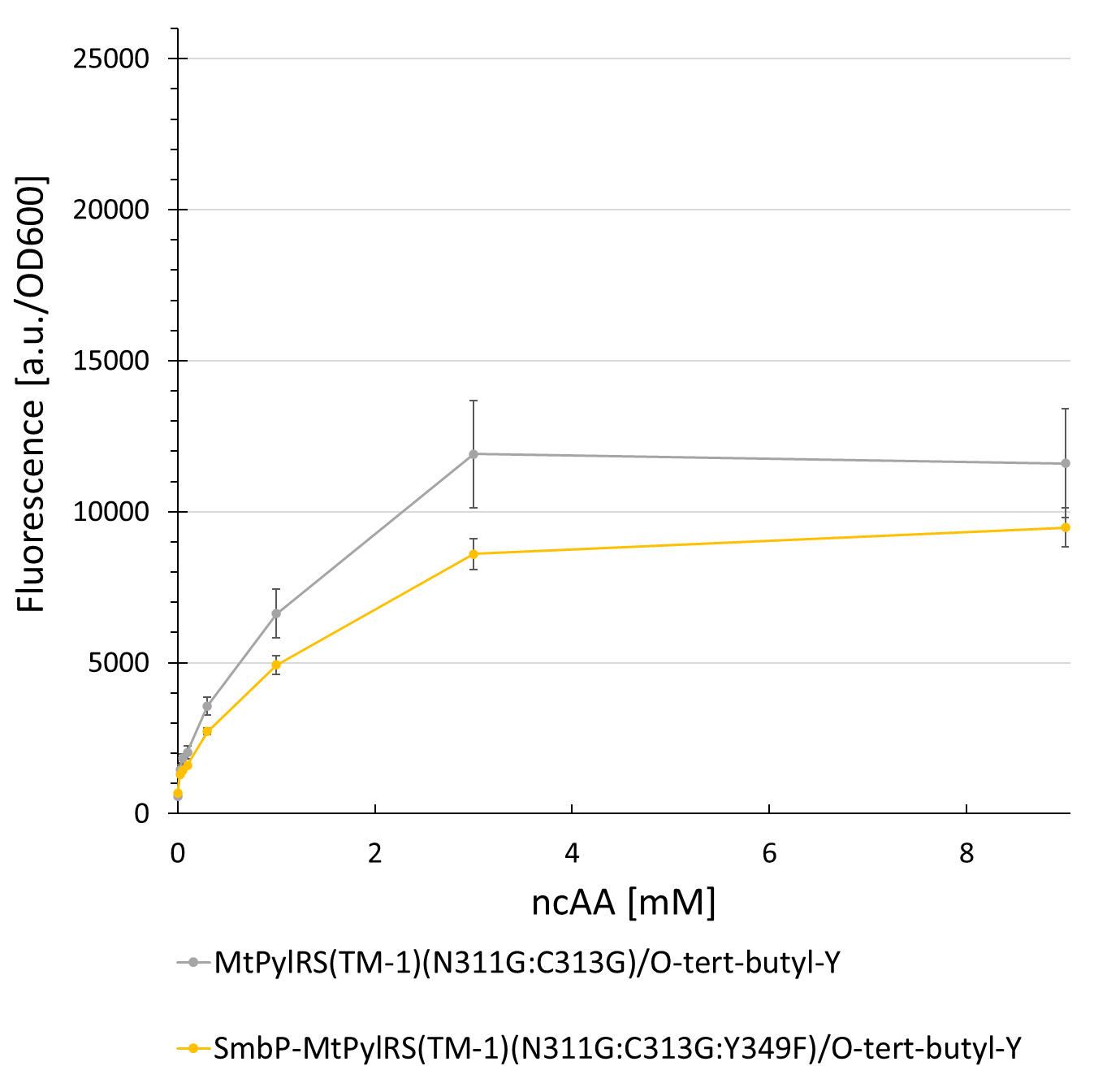


**Figure S61.** Concentration-dependent protein production for two different PylRS double Gly mutants. Fluorescence intensity of intact *E. coli* BL21(DE3) cells expressing the SUMO-sfGFP(R2 amber) reporter. Endpoint measurements after 24 h with different ncAA concentrations (0.05, 0.1, 0.3, 0.6, 1, 2, and 5 mM). Data including standard deviation represents the mean of three biological replicates.


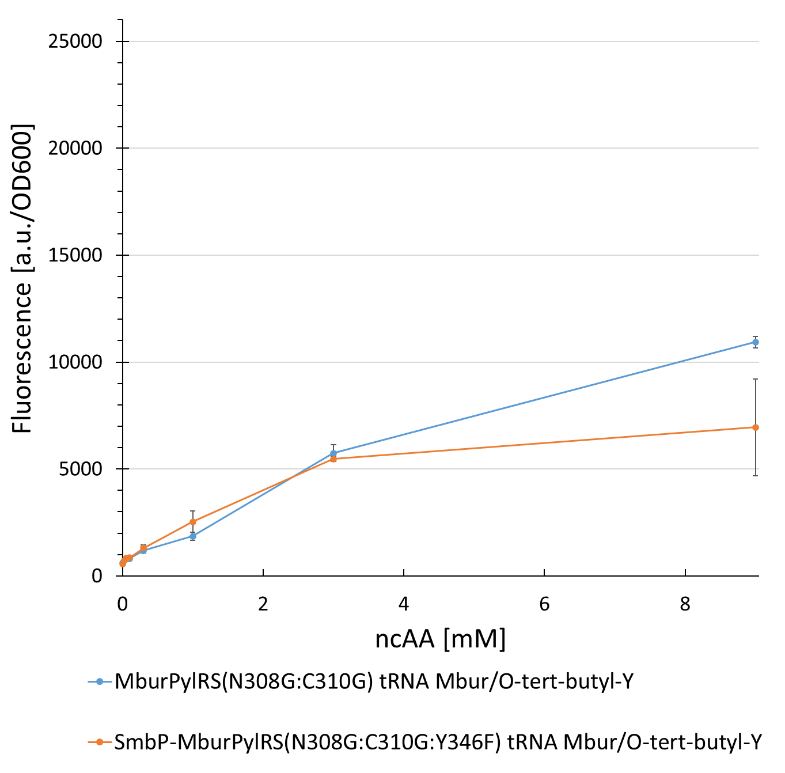


**Figure S62.** Concentration-dependent protein production for two different PylRS double Gly mutants. Fluorescence intensity of intact *E. coli* BL21(DE3) cells expressing the SUMO-sfGFP(R2 amber) reporter. Endpoint measurements after 24 h with different ncAA concentrations (0.05, 0.1, 0.3, 0.6, 1, 2, and 5 mM). Data including standard deviation represents the mean of three biological replicates.


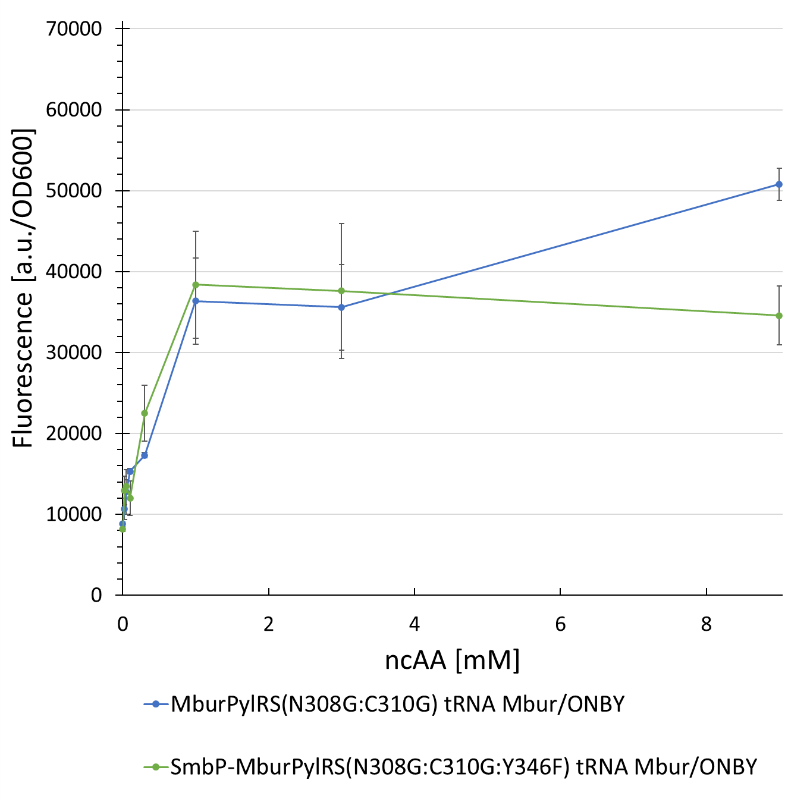


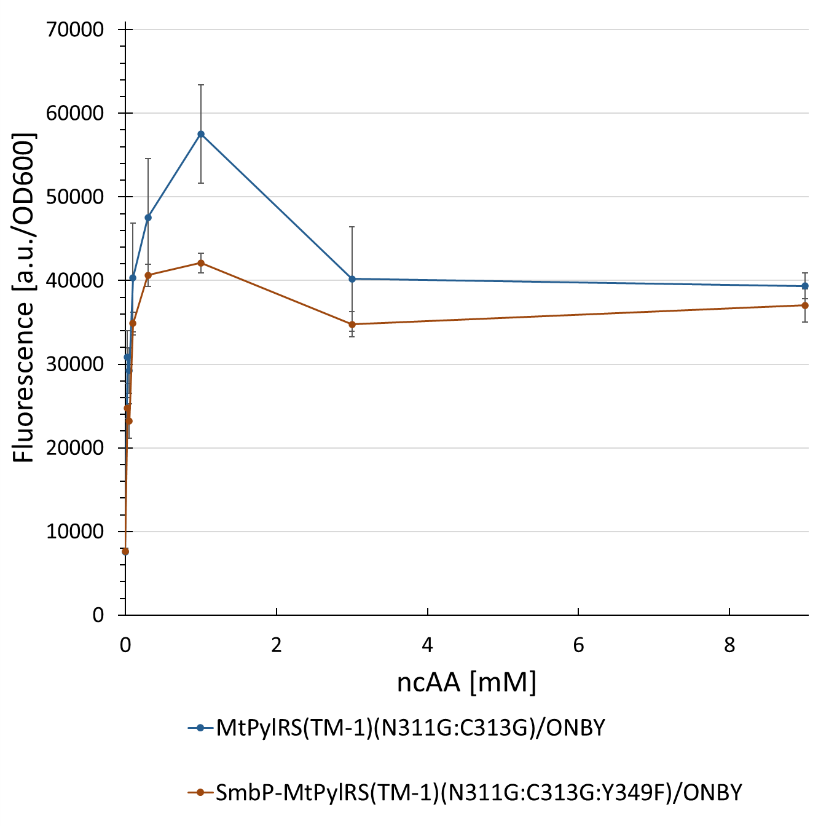
**Figure S63.** Concentration-dependent protein production for two different PylRS double Gly mutants. Fluorescence intensity of intact *E. coli* BL21(DE3) cells expressing the SUMO-sfGFP(R2 amber) reporter. Endpoint measurements after 24 h with different ncAA concentrations (0.05, 0.1, 0.3, 0.6, 1, 2, and 5 mM). Data including standard deviation represents the mean of three biological replicates.

**Figure S64.** Concentration-dependent protein production for two different PylRS double Gly mutants. Fluorescence intensity of intact *E. coli* BL21(DE3) cells expressing the SUMO-sfGFP(R2 amber) reporter. Endpoint measurements after 24 h with different ncAA concentrations (0.05, 0.1, 0.3, 0.6, 1, 2, and 5 mM). Data including standard deviation represents the mean of three biological replicates.


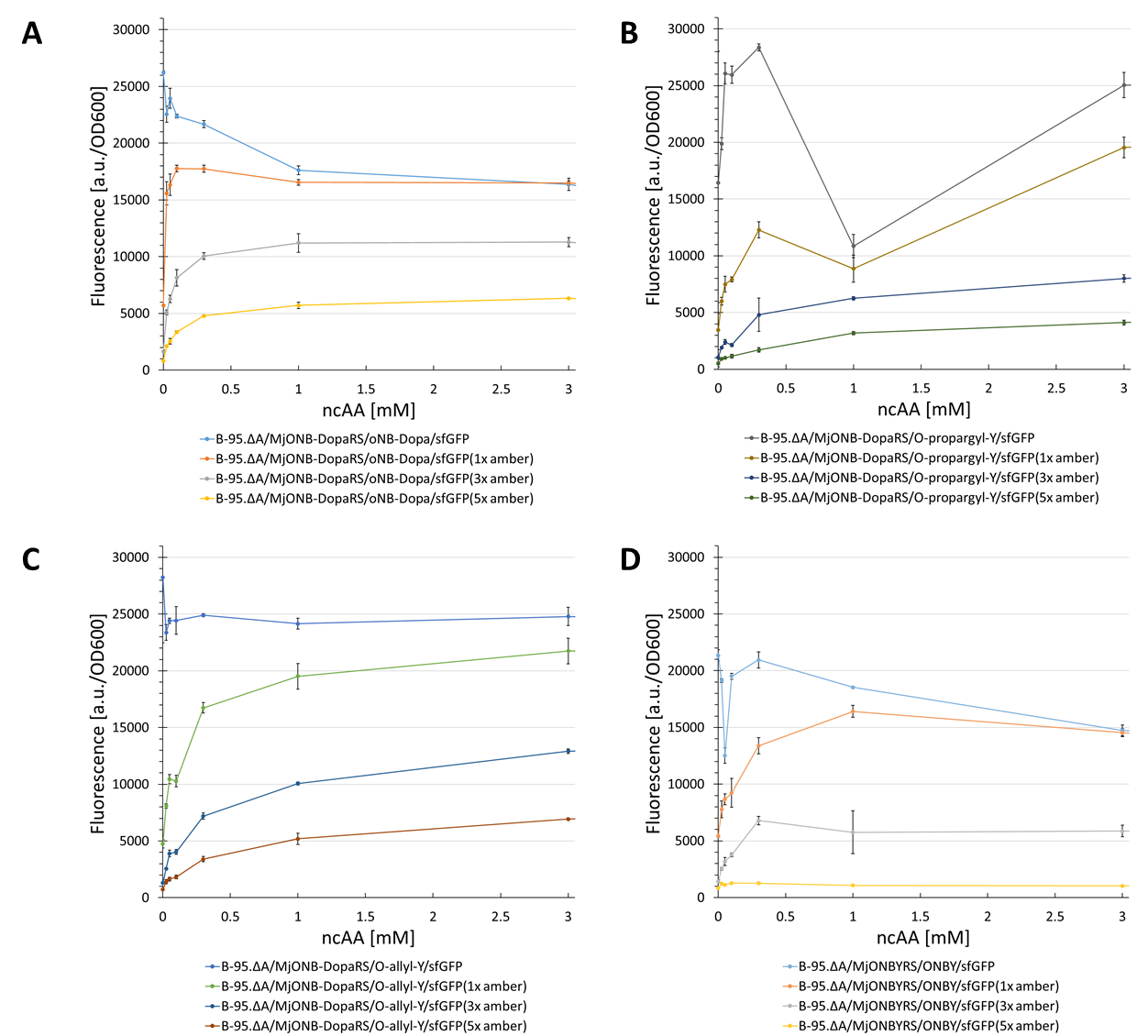


**Figure S65.** Concentration-dependent protein production for four different *Mj*TyrRS/ncAA combinations. Fluorescence intensity of intact B-95.Δ cells expressing the SUMO-sfGFP reporter containing indicated number of stop codons. Endpoint measurements after 24 h with different ncAA concentrations (0.025, 0.05, 0.1, 0.3, 1, 3, and 9 (not shown) mM). Data including standard deviation represents the mean of three biological replicates.


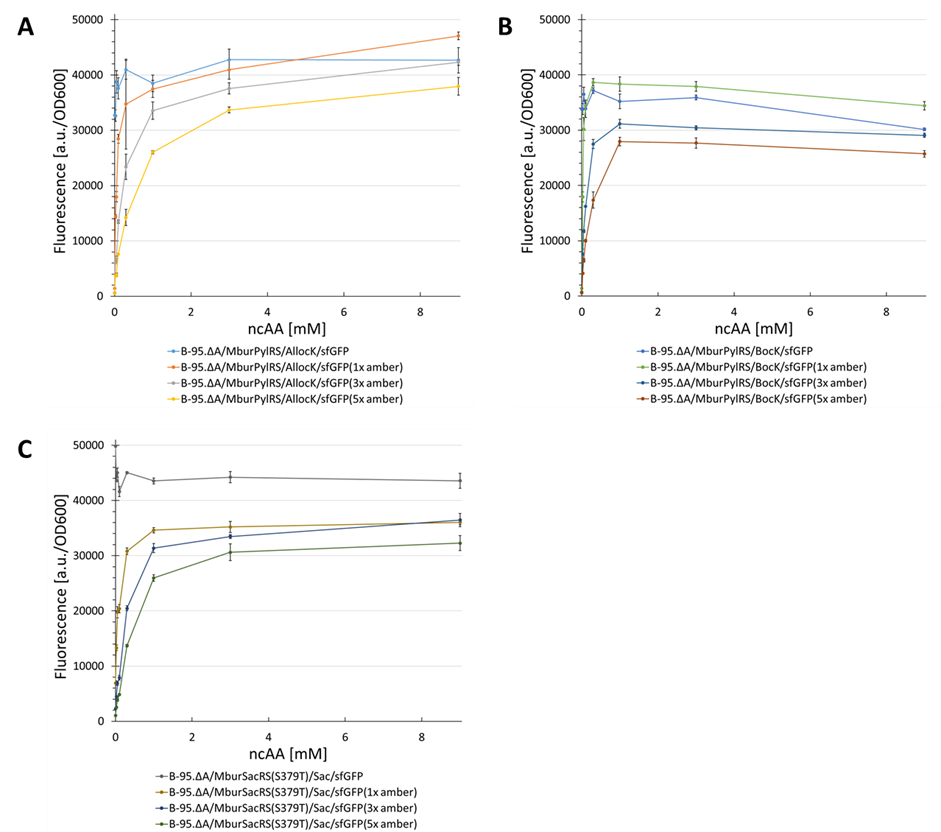


**Figure S66.** Concentration-dependent protein production for three different MburPylRSRS/ncAA combinations. Fluorescence intensity of intact B-95.Δ cells expressing the SUMO-sfGFP reporter containing indicated number of stop codons. Endpoint measurements after 24 h with different ncAA concentrations (0.025, 0.05, 0.1, 0.3, 1, 3, and 9 mM). Data including standard deviation represents the mean of three biological replicates.

## DNA/RNA Sequences

### Used PylRS

The PylRS amino acid and tRNA^Pyl^ sequences used in this study are found in the supplemental excel sheet. All PylRS sequences were codon optimized for *E. coli*. Sequences for the ΔN PylRS class were taken from the publication of Dunkelmann et al.^18^

### Reporter Constructs

1. SUMO-sfGFP(1x amber)-His_6_ reporter construct with amber codons at position R2

ATGGGCAGCAGCGACTCCGAAGTCAATCAAGAAGCTAAGCCAGAGGTCAAGCCAGAAGTCAAGCCTGAGACTCACATCAATTTAAAGGTGTCCGATGGATCTTCAGAGATCTTCTTCAAGATCAAAAAGACCACTCCTCTGCGTCGTCTGATGGAAGCGTTCGCTAAAAGACAGGGTAAGGAAATGGACTCCTTAAGATTCTTGTACGACGGTATTAGAATCCAAGCTGATCAGACCCCTGAAGATTTGGACATGGAGGATAACGATATTATTGAGGCTCATCGCGAACAGATTGGTGGCATGTAGAAAGGCGAAGAGCTGTTCACTGGTGTCGTCCCTATTCTGGTGGAACTGGATGGTGATGTCAACGGTCATAAGTTTTCCGTGCGTGGCGAGGGTGAAGGTGACGCAACTAATGGTAAACTGACGCTGAAGTTCATCTGTACTACTGGTAAACTGCCGGTACCTTGGCCGACTCTGGTAACGACGCTGACTTATGGTGTTCAGTGCTTTGCTCGTTATCCGGACCATATGAAGCAGCATGACTTCTTCAAGTCCGCCATGCCGGAAGGCTATGTGCAGGAACGCACGATTTCCTTTAAGGATGACGGCACGTACAAAACGCGTGCGGAAGTGAAATTTGAAGGCGATACCCTGGTAAACCGCATTGAGCTGAAAGGCATTGACTTTAAAGAAGACGGCAATATCCTGGGCCATAAGCTGGAATACAATTTTAACAGCCACAATGTTTACATCACCGCCGATAAACAAAAAAATGGCATTAAAGCGAATTTTAAAATTCGCCACAACGTGGAGGATGGCAGCGTGCAGCTGGCTGATCACTACCAGCAAAACACTCCAATCGGTGATGGTCCTGTTCTGCTGCCAGACAATCACTATCTGAGCACGCAAAGCGTTCTGTCTAAAGATCCGAACGAGAAACGCGATCATATGGTTCTGCTGGAGTTCGTAACCGCAGCGGGCATCACGCATGGTATGGATGAACTGTACAAAAGCGCTCATCATCATCATCATCACTAA

1. SUMO-sfGFP(3x amber)-His_6_ reporter construct with amber codons at positions R2, N39 and K101

ATGGGCAGCAGCGACTCCGAAGTCAATCAAGAAGCTAAGCCAGAGGTCAAGCCAGAAGTCAAGCCTGAGACTCACATCAATTTAAAGGTGTCCGATGGATCTTCAGAGATCTTCTTCAAGATCAAAAAGACCACTCCTCTGCGTCGTCTGATGGAAGCGTTCGCTAAAAGACAGGGTAAGGAAATGGACTCCTTAAGATTCTTGTACGACGGTATTAGAATCCAAGCTGATCAGACCCCTGAAGATTTGGACATGGAGGATAACGATATTATTGAGGCTCATCGCGAACAGATTGGTGGCATGTAGAAAGGCGAAGAGCTGTTCACTGGTGTCGTCCCTATTCTGGTGGAACTGGATGGTGATGTCAACGGTCATAAGTTTTCCGTGCGTGGCGAGGGTGAAGGTGACGCAACTTAGGGTAAACTGACGCTGAAGTTCATCTGTACTACTGGTAAACTGCCGGTACCTTGGCCGACTCTGGTAACGACGCTGACTTATGGTGTTCAGTGCTTTGCTCGTTATCCGGACCATATGAAGCAGCATGACTTCTTCAAGTCCGCCATGCCGGAAGGCTATGTGCAGGAACGCACGATTTCCTTTTAGGATGACGGCACGTACAAAACGCGTGCGGAAGTGAAATTTGAAGGCGATACCCTGGTAAACCGCATTGAGCTGAAAGGCATTGACTTTAAAGAAGACGGCAATATCCTGGGCCATAAGCTGGAATACAATTTTAACAGCCACAATGTTTACATCACCGCCGATAAACAAAAAAATGGCATTAAAGCGAATTTTAAAATTCGCCACAACGTGGAGGATGGCAGCGTGCAGCTGGCTGATCACTACCAGCAAAACACTCCAATCGGTGATGGTCCTGTTCTGCTGCCAGACAATCACTATCTGAGCACGCAAAGCGTTCTGTCTAAAGATCCGAACGAGAAACGCGATCATATGGTTCTGCTGGAGTTCGTAACCGCAGCGGGCATCACGCATGGTATGGATGAACTGTACAAAAGCGCTCATCATCATCATCATCACTAA

1. SUMO-sfGFP(5x amber)-His_6_ reporter construct with amber codons at positions R2, N39, K101, E132 and D190

ATGGGCAGCAGCGACTCCGAAGTCAATCAAGAAGCTAAGCCAGAGGTCAAGCCAGAAGTCAAGCCTGAGACTCACATCAATTTAAAGGTGTCCGATGGATCTTCAGAGATCTTCTTCAAGATCAAAAAGACCACTCCTCTGCGTCGTCTGATGGAAGCGTTCGCTAAAAGACAGGGTAAGGAAATGGACTCCTTAAGATTCTTGTACGACGGTATTAGAATCCAAGCTGATCAGACCCCTGAAGATTTGGACATGGAGGATAACGATATTATTGAGGCTCATCGCGAACAGATTGGTGGCATGTAGAAAGGCGAAGAGCTGTTCACTGGTGTCGTCCCTATTCTGGTGGAACTGGATGGTGATGTCAACGGTCATAAGTTTTCCGTGCGTGGCGAGGGTGAAGGTGACGCAACTTAGGGTAAACTGACGCTGAAGTTCATCTGTACTACTGGTAAACTGCCGGTACCTTGGCCGACTCTGGTAACGACGCTGACTTATGGTGTTCAGTGCTTTGCTCGTTATCCGGACCATATGAAGCAGCATGACTTCTTCAAGTCCGCCATGCCGGAAGGCTATGTGCAGGAACGCACGATTTCCTTTTAGGATGACGGCACGTACAAAACGCGTGCGGAAGTGAAATTTGAAGGCGATACCCTGGTAAACCGCATTGAGCTGAAAGGCATTGACTTTAAATAGGACGGCAATATCCTGGGCCATAAGCTGGAATACAATTTTAACAGCCACAATGTTTACATCACCGCCGATAAACAAAAAAATGGCATTAAAGCGAATTTTAAAATTCGCCACAACGTGGAGGATGGCAGCGTGCAGCTGGCTGATCACTACCAGCAAAACACTCCAATCGGTTAGGGTCCTGTTCTGCTGCCAGACAATCACTATCTGAGCACGCAAAGCGTTCTGTCTAAAGATCCGAACGAGAAACGCGATCATATGGTTCTGCTGGAGTTCGTAACCGCAGCGGGCATCACGCATGGTATGGATGAACTGTACAAAAGCGCTCATCATCATCATCATCACTAA

## tRNA Sequences and secondary structure prediction

To evaluate structural stability and folding characteristics of selected tRNA^Pyl^ variants, secondary structure predictions were performed using the ViennaRNA-based algorithm implemented in Geneious (**Figure S67**). Predicted minimum free energy (ΔG) values provide a comparative estimate of thermodynamic stability at 37 °C.


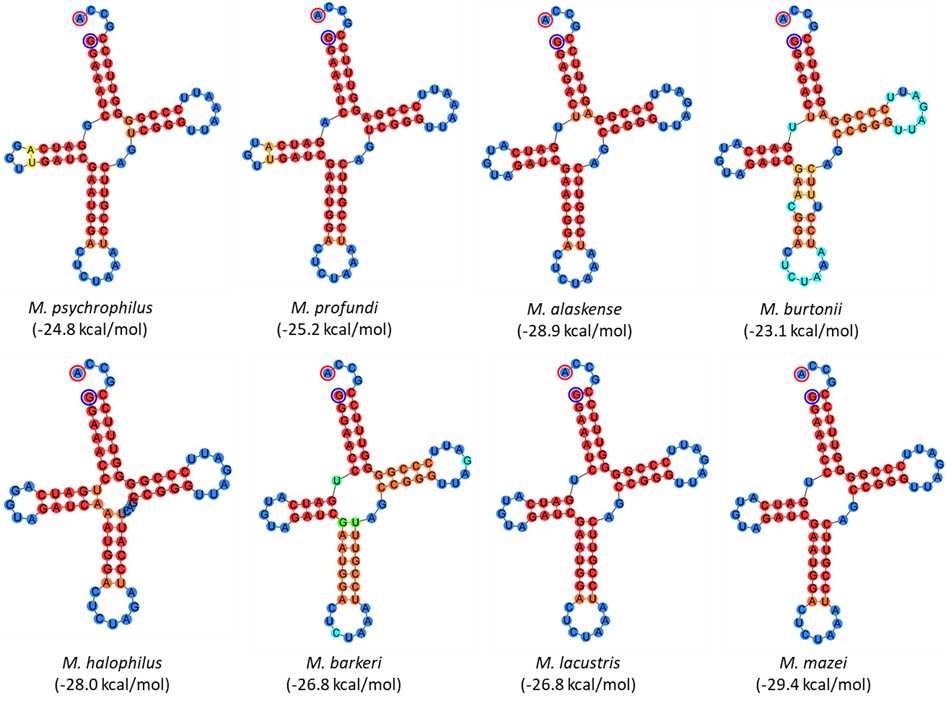
The overall cloverleaf architecture is preserved across variants, with only minor differences in predicted base-pairing probabilities. Variations in ΔG values suggest modest differences in folding stability, which may influence tRNA performance under physiological conditions. However, no major structural disruptions are predicted, indicating that all selected tRNA^Pyl^ constructs maintain canonical secondary structure features required for functionality.

**Figure S67.** Predicted secondary structures of selected tRNAPyl variants. Free energy (ΔG) values, calculated at 37 °C, are indicated in brackets. Nucleotide pairing probabilities are color-coded (red = high, green = intermediate, blue = low). Secondary structure prediction and free energy calculations were performed using Geneious (version 7.1.9), which incorporates the ViennaRNA Package.¹⁹ The nucleotide marked with a blue circle denotes the 5′ end, and the nucleotide marked with a red circle denotes the 3′ end.

## Deconvoluted ESI-MS spectra of intact ncAA-containing protein variants

All intact protein masses were measured by electrospray LC-MS and evaluated using maximum entropy deconvolution, as described in the Methods section of the main manuscript.


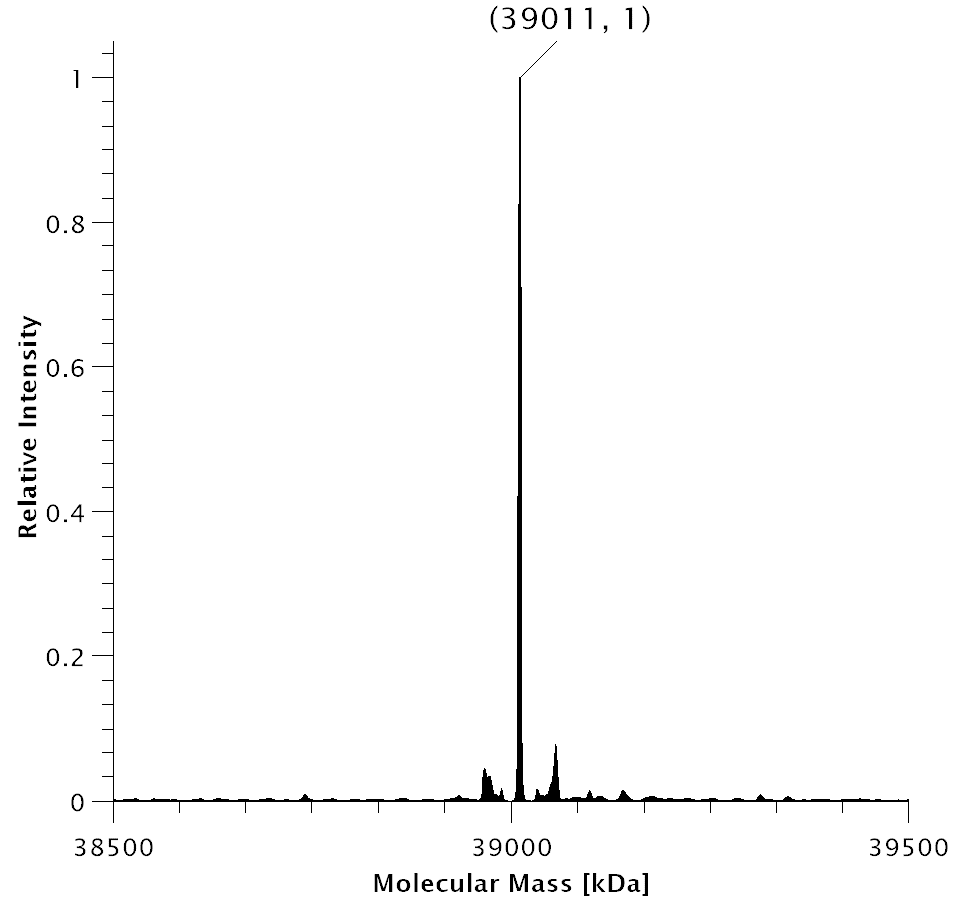


**Figure S68**. Deconvoluted ESI-MS spectrum of SUMO-sfGFP(1x(**38**))-His_6_ produced in *E. coli* BL21(DE3) with co-expression of **Mbur(C310W:S379T)**. Expected protein mass: 39011 Da. Observed mass: 39011 Da.


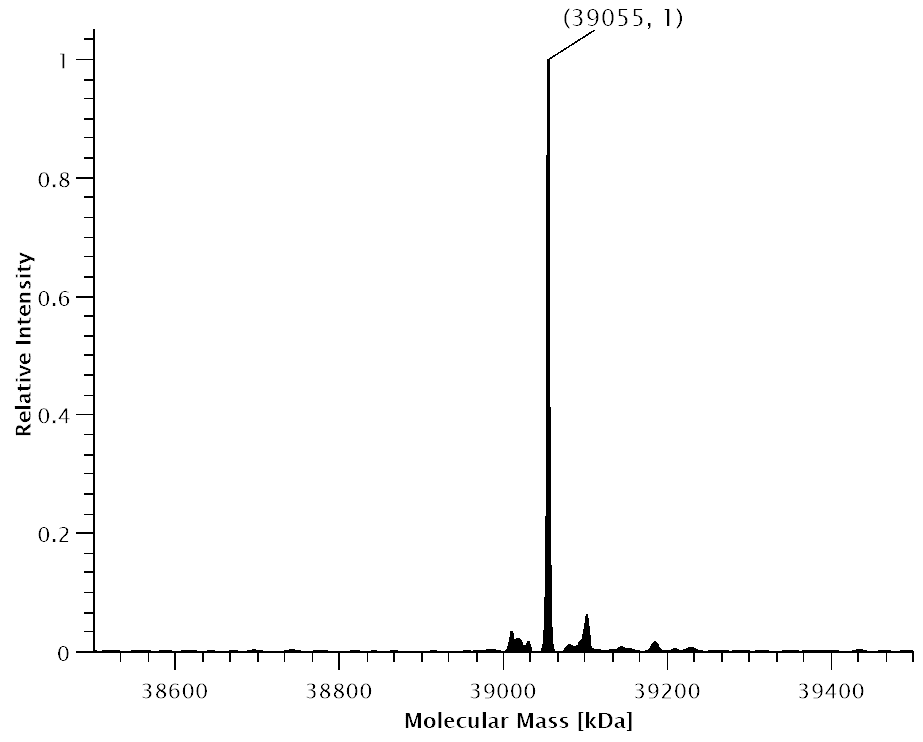


**Figure S69.** Deconvoluted ESI-MS spectrum of SUMO-sfGFP(3x **38**)-His_6_ production in *E. coli* BL21(DE3) with co-expression of **Mbur(C310W:S379T)**. Expected protein mass: 39055 Da. Observed mass: 39055 Da.


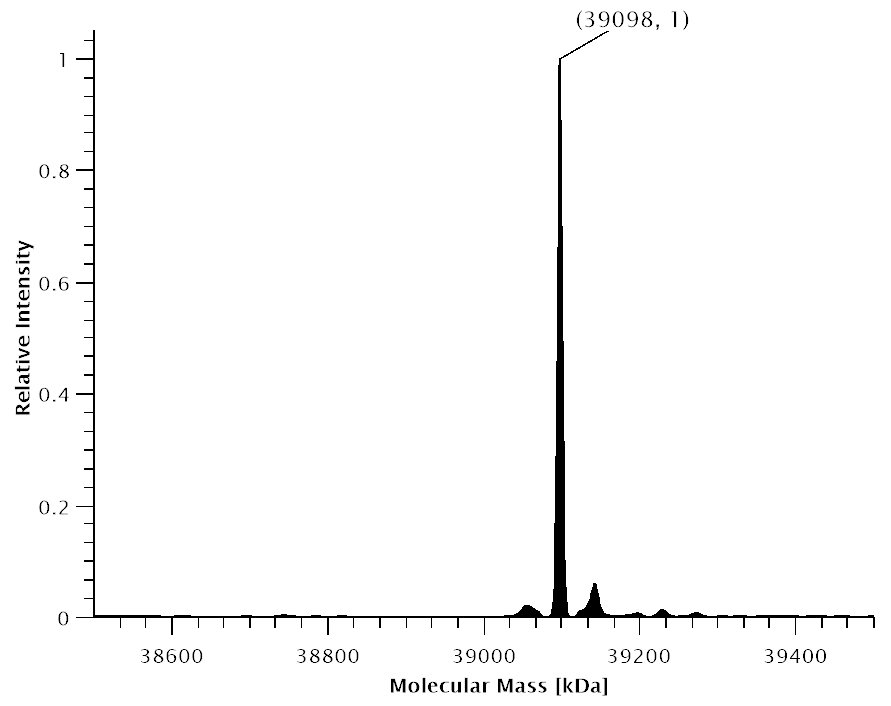


**Figure S70.** Deconvoluted ESI-MS spectrum of SUMO-sfGFP(5x(**38**)-His_6_ production in *E. coli* BL21(DE3) with co-expression of **Mbur(C310W:W379T)**. Expected protein mass: 39098 Da. Observed mass: 39098 Da.


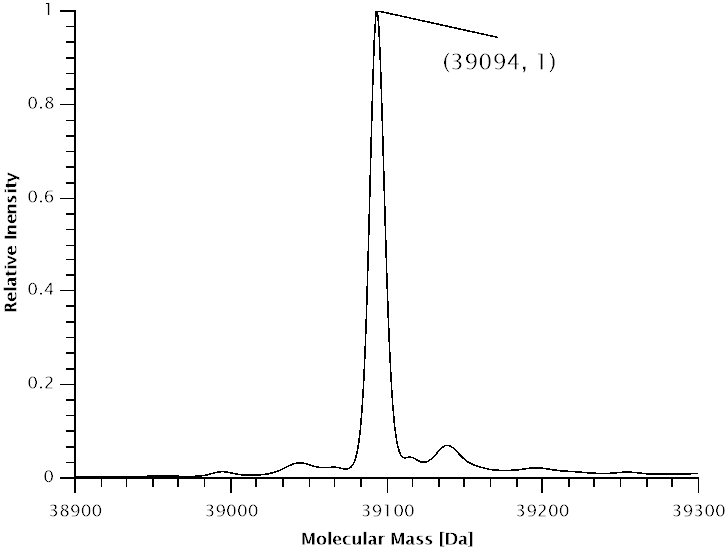


**Figure S71.** Deconvoluted ESI-MS spectrum of SUMO-sfGFP(1x(**1**))-His_6_ production in *E. coli* BL21(DE3) with co-expression of **Mm**. Expected protein mass: 39096 Da. Observed mass: 39094 Da.


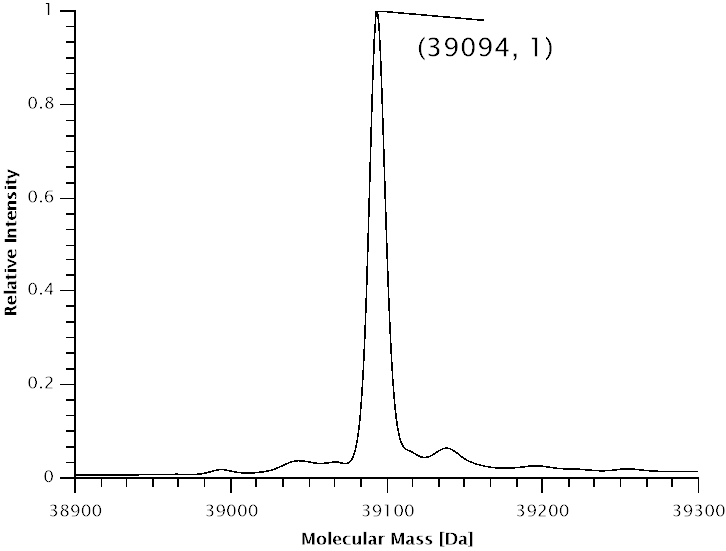


**Figure S72.** Deconvoluted ESI-MS spectrum of SUMO-sfGFP(1x(**1**))-His_6_ production in *E. coli* BL21(DE3) with co-expression of **Mbur**. Expected protein mass: 39096 Da. Observed mass: 39094 Da.


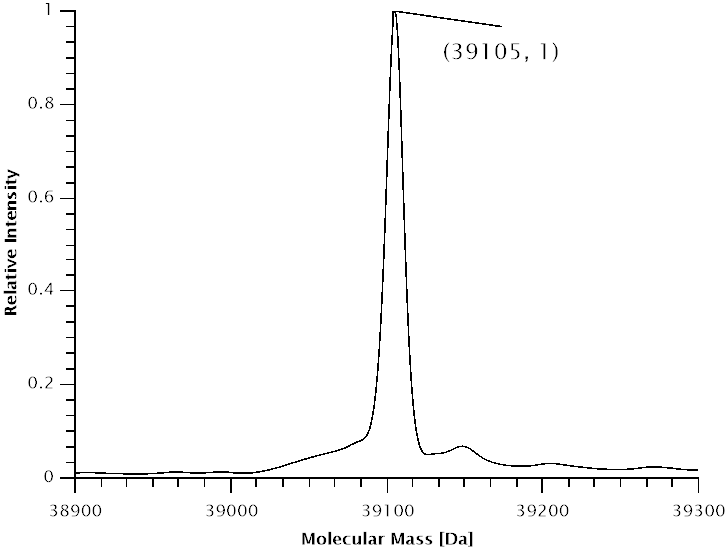


**Figure S73.** Deconvoluted ESI-MS spectrum of SUMO-sfGFP(1x(**4**))-His_6_ production in *E. coli* BL21(DE3) with co-expression of **Mm**. Expected protein mass: 39099 Da. Observed mass: 39095 Da.


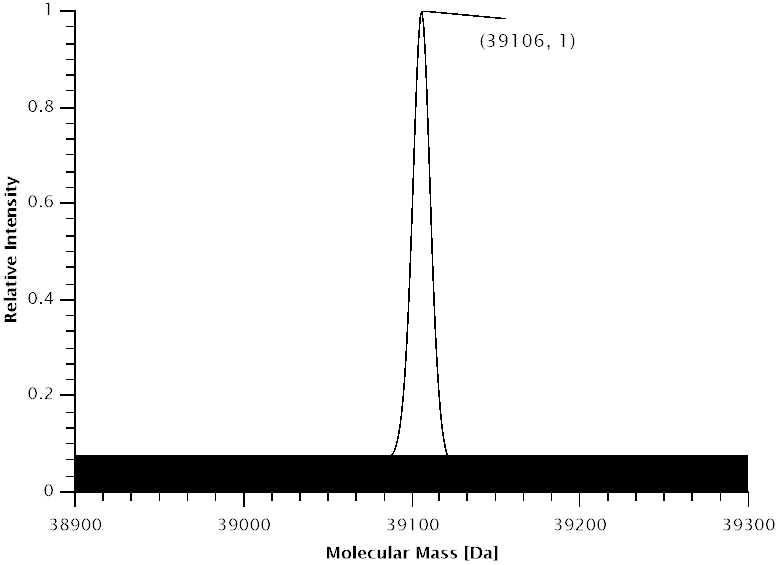


**Figure S74.** Deconvoluted ESI-MS spectrum of SUMO-sfGFP(1x(**4**))-His_6_ production in *E. coli* BL21(DE3) with co-expression of **Mbur**. Expected protein mass: 39099 Da. Observed mass: 39096 Da.


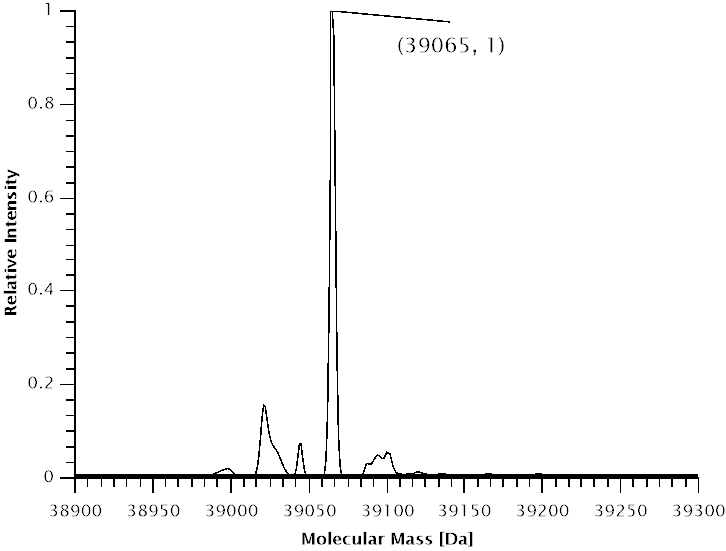


**Figure S75.** Deconvoluted ESI-MS spectrum of SUMO-sfGFP(1x(**9**))-His_6_ production in *E. coli* BL21(DE3) with co-expression of **Mm**. Expected protein mass: 39069 Da. Observed mass: 39065 Da.


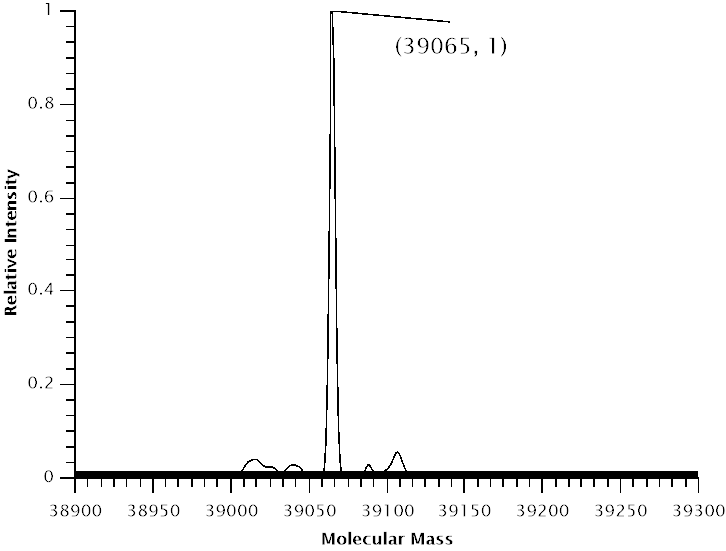


**Figure S76.** Deconvoluted ESI-MS spectrum of SUMO-sfGFP(1x(**9**))-His_6_ production in *E. coli* BL21(DE3) with co-expression of **Mbur**. Expected protein mass: 39069 Da. Observed mass: 39065 Da.


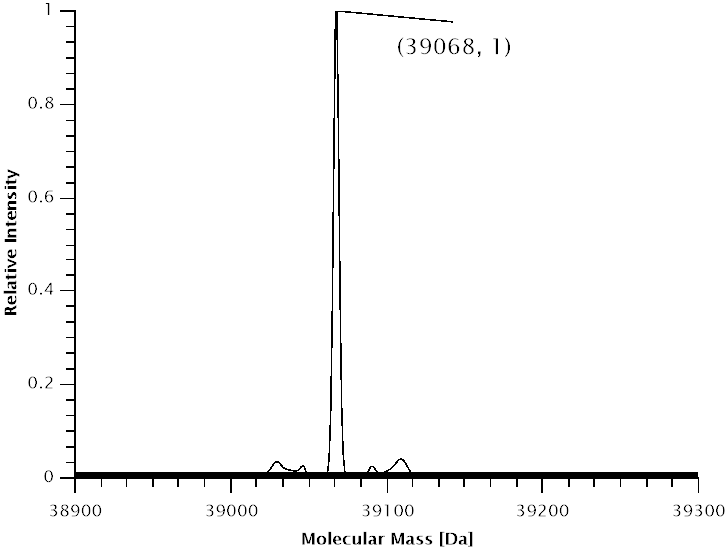


**Figure S77.** Deconvoluted ESI-MS spectrum of SUMO-sfGFP(1x(**11**))-His_6_ production in *E. coli* BL21(DE3) with co-expression of **Mm**. Expected protein mass: 39071 Da. Observed mass: 39068 Da.


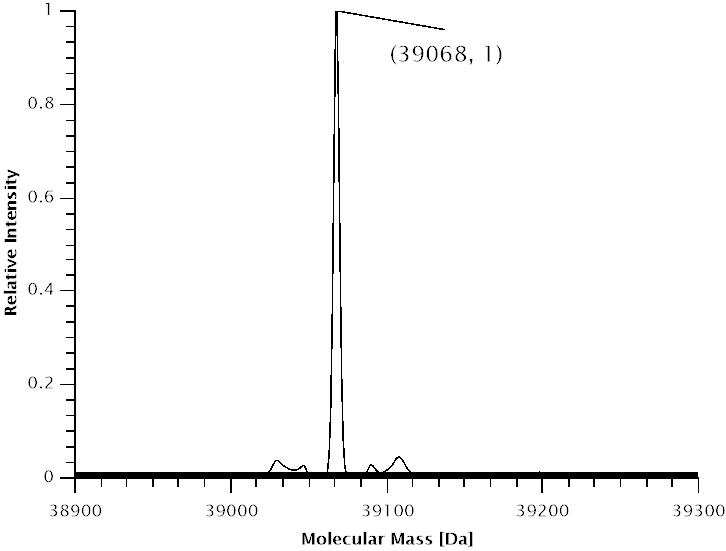


**Figure S78.** Deconvoluted ESI-MS spectrum of SUMO-sfGFP(1x(**11**))-His_6_ production in *E. coli* BL21(DE3) with co-expression of **Mbur**. Expected protein mass: 39071 Da. Observed mass: 39068 Da.


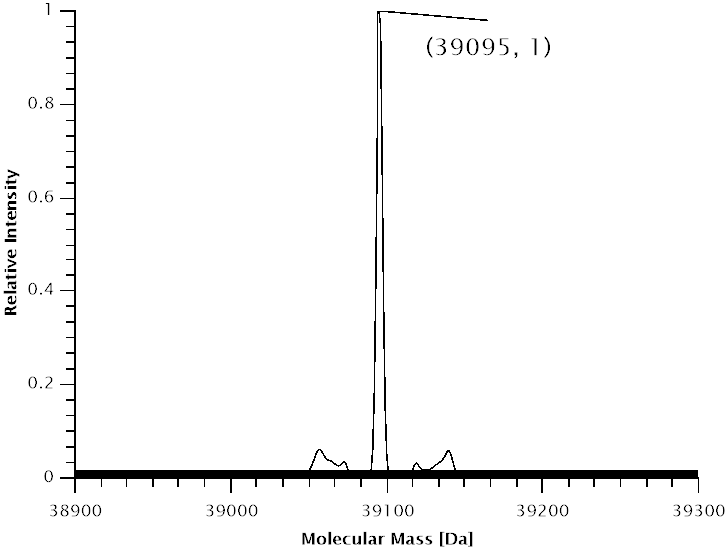


**Figure S79.** Deconvoluted ESI-MS spectrum of SUMO-sfGFP(1x(**13**))-His_6_ production in *E. coli* BL21(DE3) with co-expression of **Mm**. Expected protein mass: 39099 Da. Observed mass: 39095 Da.


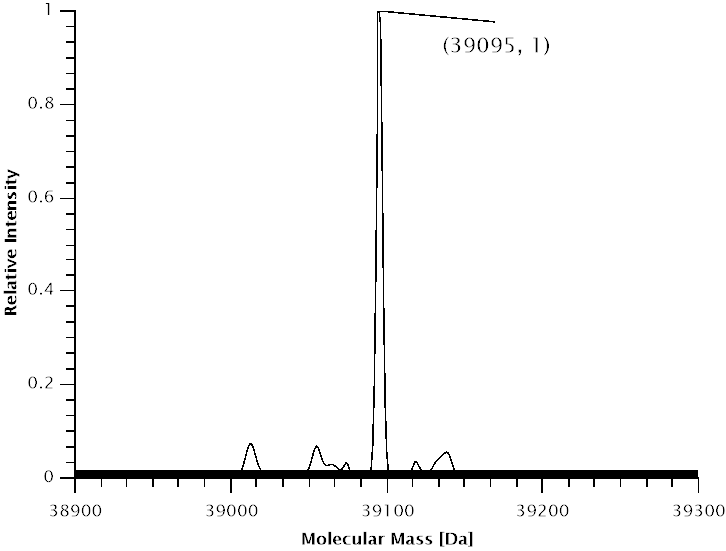


**Figure S80.** Deconvoluted ESI-MS spectrum of SUMO-sfGFP(1x(**13**))-His_6_ production in *E. coli* BL21(DE3) with co-expression of **Mbur**. Expected protein mass: 39099 Da. Observed mass: 39095 Da.


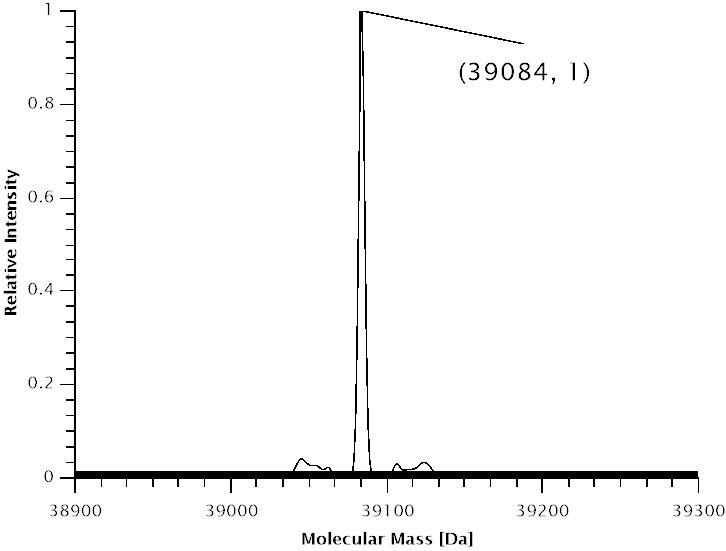


**Figure S81.** Deconvoluted ESI-MS spectrum of SUMO-sfGFP(1x(**8**))-His_6_ production in *E. coli* BL21(DE3) with co-expression of **Mm**. Expected protein mass: 39087 Da. Observed mass: 39084 Da.


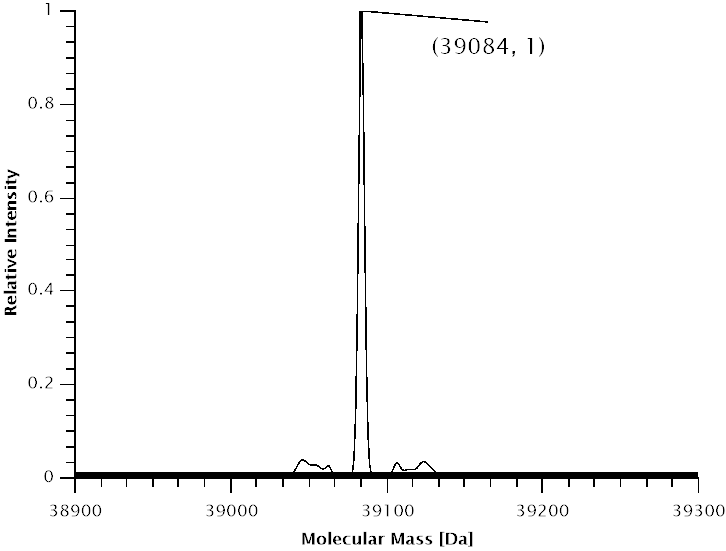


**Figure S82.** Deconvoluted ESI-MS spectrum of SUMO-sfGFP(1x(**8**))-His_6_ production in *E. coli* BL21(DE3) with co-expression of **Mbur**. Expected protein mass: 39087 Da. Observed mass: 39084 Da.


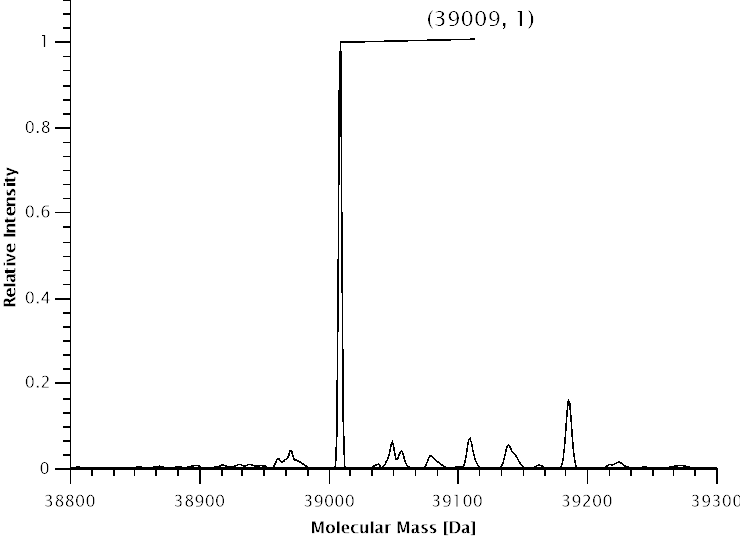


**Figure S83.** Deconvoluted ESI-MS spectrum of SUMO-sfGFP(1x(**35**))-His_6_ production in *E. coli* B95.ΔA with co-expression of **SmbP-Mb(C313W:W382T)**. Expected protein mass: 39008.9 Da. Observed mass: 39009 Da.


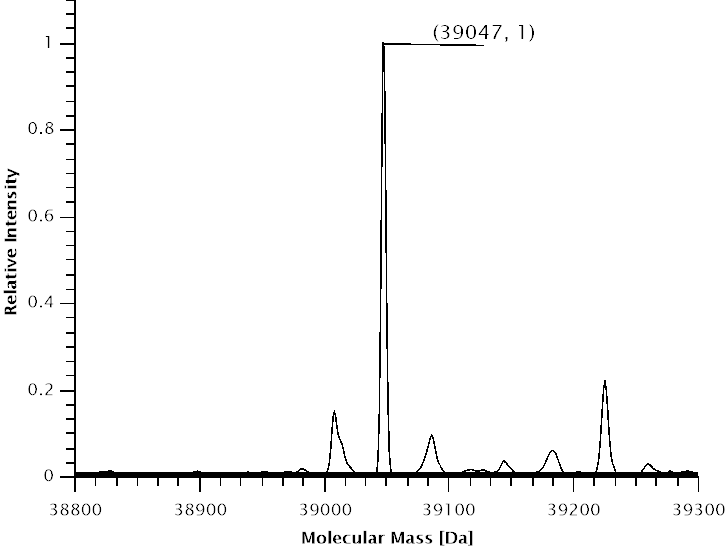


**Figure S84.** Deconvoluted ESI-MS spectrum of SUMO-sfGFP(3x(**35**))-His_6_ production in *E. coli* B95.ΔA with co-expression of **SmbP-Mb(C313W:W382T)**. Expected protein mass: 39049.0 Da. Observed mass: 39047 Da.


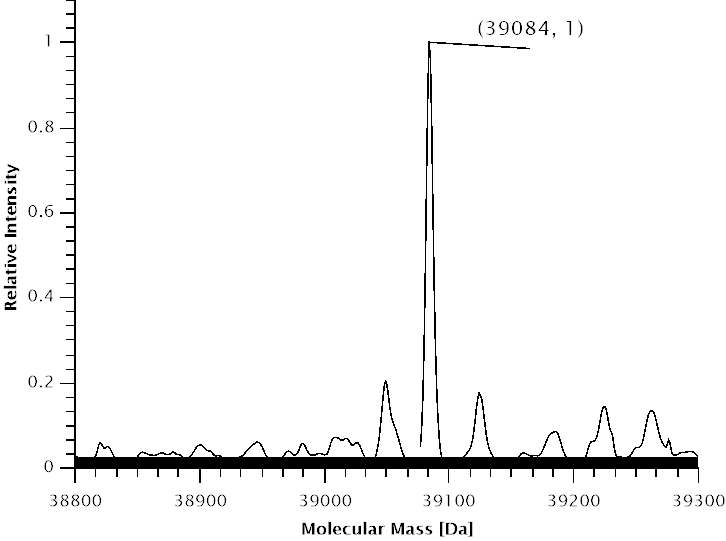


**Figure S85.** Deconvoluted ESI-MS spectrum of SUMO-sfGFP(5x(**35**))-His_6_ production in *E. coli* B95.ΔA with co-expression of **SmbP-Mb(C313W:W382T)**. Expected protein mass: 39087.2 Da. Observed mass: 39084 Da.


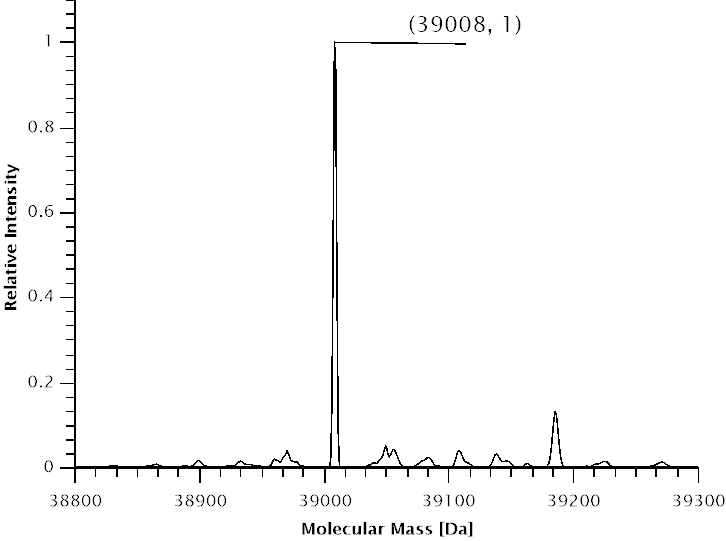


**Figure S86.** Deconvoluted ESI-MS spectrum of SUMO-sfGFP(1x(**35**))-His_6_ production in *E. coli* B95.ΔA with co-expression of **Mbur(C310W:W379T)**. Expected protein mass: 39008.9 Da. Observed mass: 39008 Da.


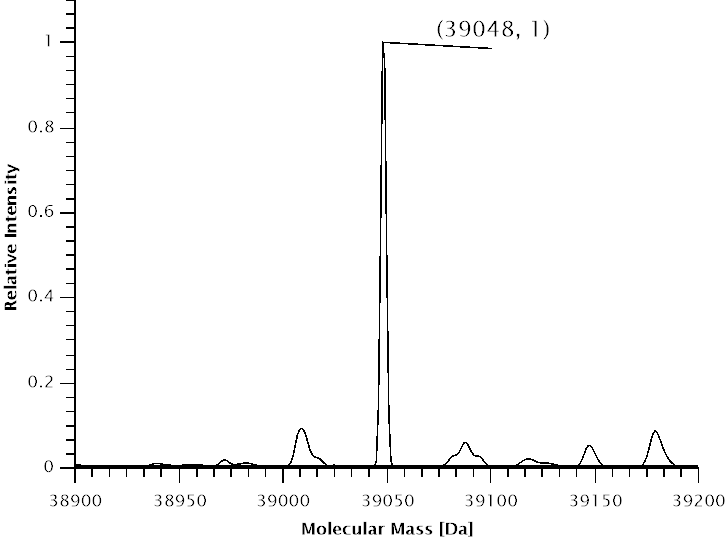


**Figure S87.** Deconvoluted ESI-MS spectrum of SUMO-sfGFP(3x(**35**))-His_6_ production in *E. coli* B95.ΔA with co-expression of **Mbur(C310W:S379T)**. Expected protein mass: 39049.0 Da. Observed mass: 39048 Da.


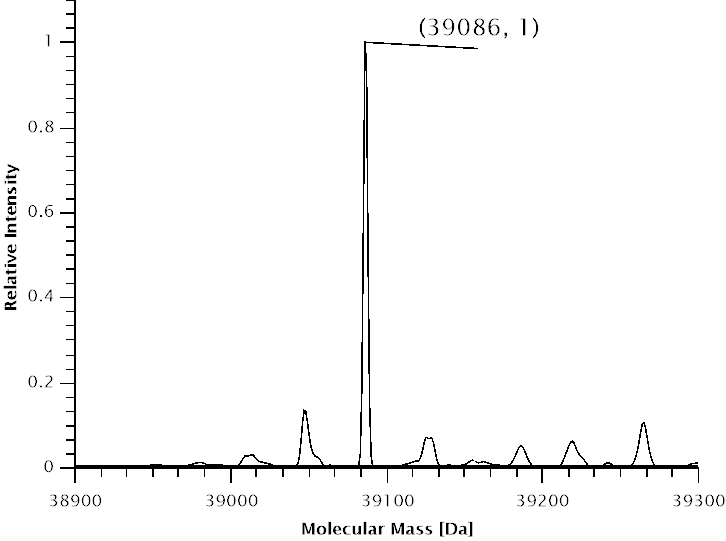


**Figure S88.** Deconvoluted ESI-MS spectrum of SUMO-sfGFP(5x(**35**))-His_6_ production in *E. coli* B95.ΔA with co-expression of **Mbur(C310W:S379T)**. Expected protein mass: 39087.2 Da. Observed mass: 39086 Da.


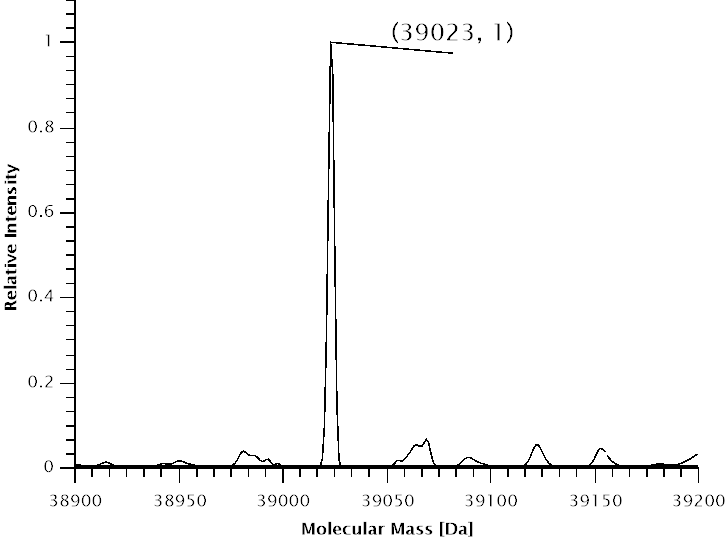


**Figure S89.** Deconvoluted ESI-MS spectrum of SUMO-sfGFP-His_6_ production in *E. coli* B95.ΔA with co-expression of **SmbP-Mb(C310W:W382T)**. Expected protein mass: 39023.9 Da. Observed mass: 39023 Da.

**
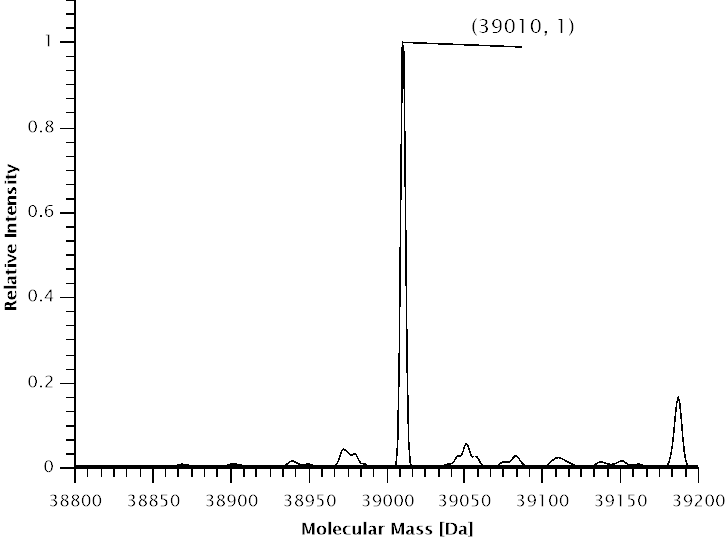
**

**Figure S90.** Deconvoluted ESI-MS spectrum of SUMO-sfGFP(1x(**38**))-His_6_ production in *E. coli* B95.ΔA with co-expression of **SmbP-Mb(C310W:W382T)**. Expected protein mass: 39010.9 Da. Observed mass: 39010 Da.

**
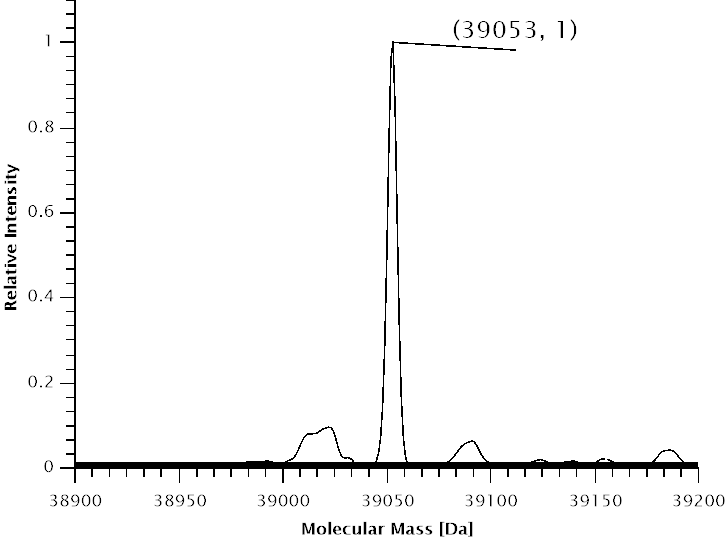
**

**Figure S91.** Deconvoluted ESI-MS spectrum of SUMO-sfGFP(3x(**38**))-His_6_ production in *E. coli* B95.ΔA with co-expression of **SmbP-Mb(C310W:W382T)**. Expected protein mass: 39055.2 Da. Observed mass: 39053 Da.


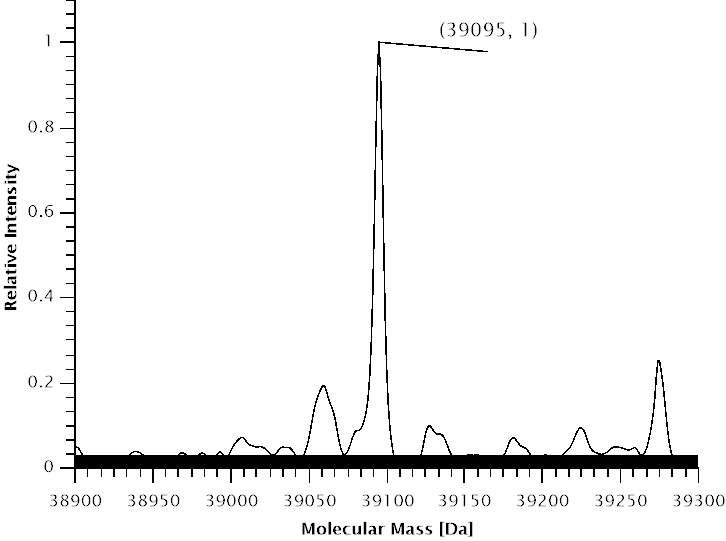


**Figure S92.** Deconvoluted ESI-MS spectrum of SUMO-sfGFP(5x(**38**))-His_6_ production in *E. coli* B95.ΔA with co-expression of **SmbP-Mb(C310W:W382T)** Expected protein mass: 39097.5 Da. Observed mass: 39095 Da.


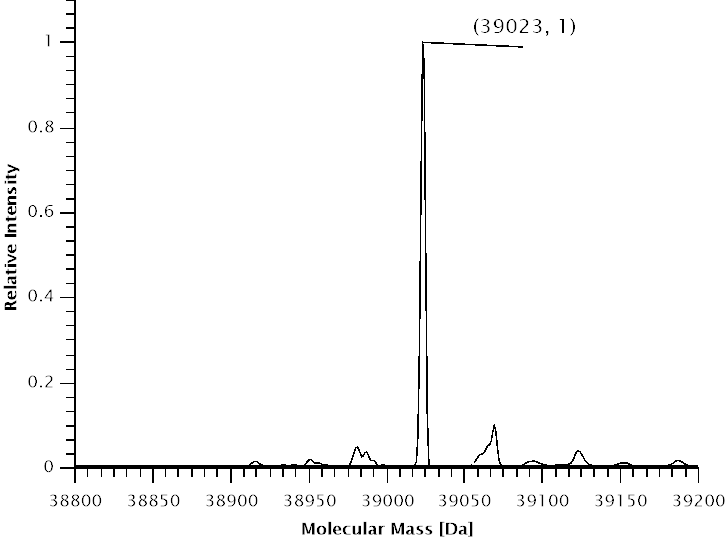


**Figure S93.** Deconvoluted ESI-MS spectrum of SUMO-sfGFP-His_6_ production in *E. coli* B95.ΔA with co-expression of **Mbur(C310W:W379T)**. Expected protein mass: 39023.9 Da. Observed mass: 39023 Da.


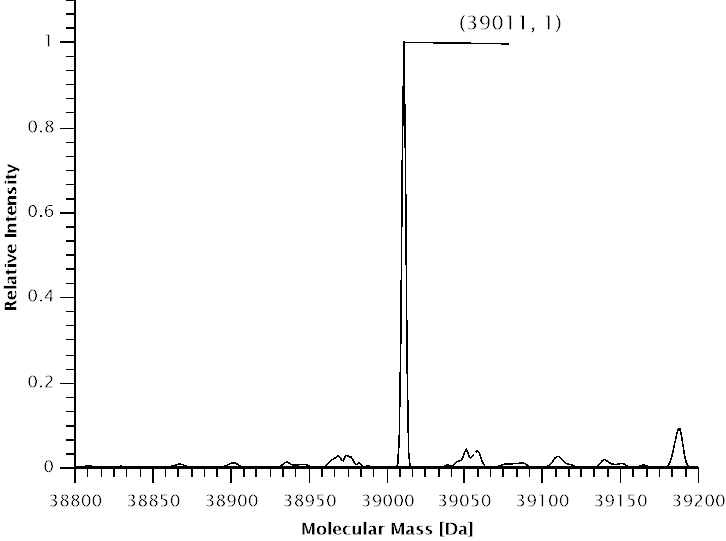


**Figure S94.** Deconvoluted ESI-MS spectrum of SUMO-sfGFP(1x(**38**))-His_6_ production in *E. coli* B95.ΔA with co-expression of **Mbur(C310W:W379T)**. Expected protein mass: 39010.9 Da. Observed mass: 39011 Da.


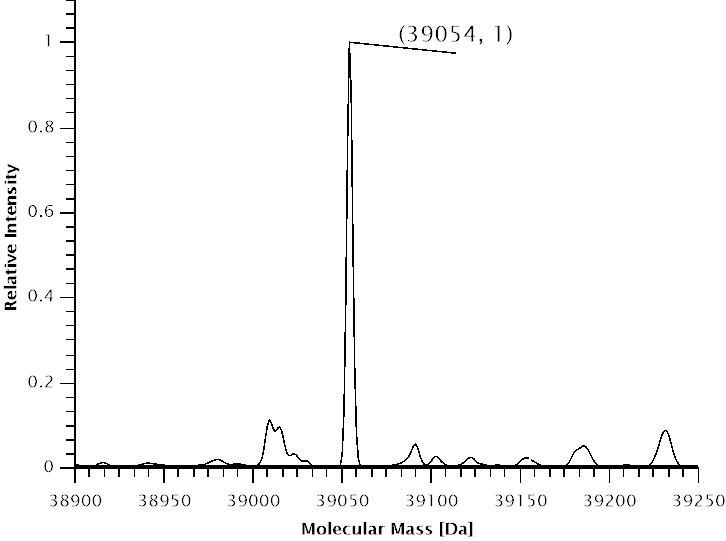


**Figure S95.** Deconvoluted ESI-MS spectrum of SUMO-sfGFP(3x(**38**))-His_6_ production in *E. coli* B95.ΔA with co-expression of **Mbur(C310W:W379T)**. Expected protein mass: 39055.2 Da. Observed mass: 39054 Da.


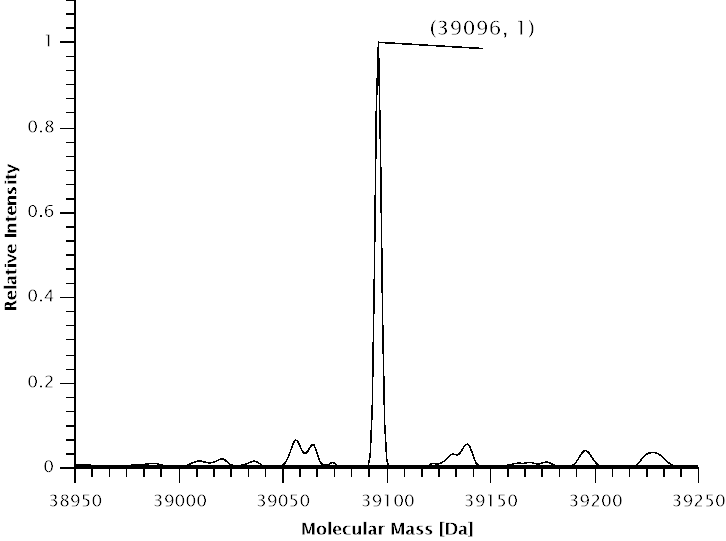


**Figure S96.** Deconvoluted ESI-MS spectrum of SUMO-sfGFP(5x(**38**))-His_6_ production in *E. coli* B95.ΔA with co-expression of **Mbur(C310W:W379T)**. Expected protein mass: 39097.5 Da. Observed mass: 39096 Da.

## ESI-MS data

**Table S1.** Setup of protein production platform with calculated and observed molecular weights of reporter proteins corresponding to a) SUMO-sfGFP(1x amber), b) SUMO-sfGFP(3x amber), c) SUMO-sfGFP(5x amber), d) SUMO-sfGFP(wild-type). The masses were determined by ESI-MS of the intact proteins (**Figure S60-S88**).

| ncAA | [mM] | E. coli strain^[a]^ | PylRS construct | reporter | calculated mass [Da] | found mass [Da]] | Δ mass [Da] |
| --- | --- | --- | --- | --- | --- | --- | --- |
| **2** | 1 | BL21 | Mm | a | 39096.0 | 39094 | 2 |
| **2** | 1 | BL21 | Mbur | a | 39096.0 | 39094 | 2 |
| **3** | 1 | BL21 | Mm | a | 39109.0 | 39105 | 4 |
| **3** | 1 | BL21 | Mbur | a | 39109.0 | 39106 | 3 |
| **9** | 3 | BL21 | Mm(N346A:C348A) | a | 39069.0 | 39065 | 4 |
| **9** | 3 | BL21 | Mbur(N308A:C310A) | a | 39069.0 | 39065 | 4 |
| **11** | 3 | BL21 | Mm(N346A:C348A) | a | 39071.0 | 39068 | 3 |
| **11** | 3 | BL21 | Mbur(N308A:C310A) | a | 39071.0 | 39068 | 3 |
| **13** | 1 | BL21 | Mm(N346A:C348A) | a | 39098.9 | 39095 | 3.9 |
| **13** | 1 | BL21 | Mbur(N308A:C310A) | a | 39098.9 | 39095 | 3.9 |
| **8** | 3 | BL21 | Mm(N346A:C348A) | a | 39087.0 | 39084 | 3 |
| **8** | 3 | BL21 | Mbur(N308A:C310A) | a | 39087.0 | 39084 | 3 |
| **38** | 0.3 | B-95.ΔA | SmbP-Mb(C313W:W382T) | d | 39023.9 | 39023 | 0.9 |
| **38** | 0.3 | B-95.ΔA | SmbP-Mb(C313W:W382T) | a | 39010.9 | 39010 | 0.9 |
| **38** | 0.3 | B-95.ΔA | SmbP-Mb(C313W:W382T) | b | 39055.2 | 39053 | 2.2 |
| **38** | 0.3 | B-95.ΔA | SmbP-Mb(C313W:W382T) | c | 39097.5 | 39095 | 2.5 |
| **38** | 0.3 | B-95.ΔA | Mbur(C310W:W379T) | d | 39023.9 | 39023 | 0.9 |
| **38** | 0.3 | B-95.ΔA | Mbur(C310W:W379T) | a | 39010.9 | 39011 | 0.1 |
| **38** | 0.3 | B-95.ΔA | Mbur(C310W:W379T) | b | 39055.2 | 39054 | 1.2 |
| **38** | 0.3 | B-95.ΔA | Mbur(C310W:W379T) | c | 39097.5 | 39096 | 1.5 |
| **35** | 1 | B-95.ΔA | SmbP-Mb(C313W:W382T) | a | 39008.9 | 39009 | 0.1 |
| **35** | 1 | B-95.ΔA | SmbP-Mb(C313W:W382T) | b | 39049.0 | 39047 | 2 |
| **35** | 1 | B-95.ΔA | SmbP-Mb(C313W:W382T) | c | 39087.2 | 39084 | 3.2 |
| **35** | 1 | B-95.ΔA | Mbur(C310W:W379T) | a | 39008.9 | 39008 | 0.9 |
| **35** | 1 | B-95.ΔA | Mbur(C310W:W379T) | b | 39049.0 | 39048 | 1 |
| **35** | 1 | B-95.ΔA | Mbur(C310W:W379T) | c | 39087.2 | 39086 | 1.2 |

[a] All DE3

## Protein yields

**Table S2.** Setup of protein production platform with calculated and observed molecular weights of reporter proteins corresponding to a) SUMO-sfGFP(1x amber), b) SUMO-sfGFP(3x amber), c) SUMO-sfGFP(5x amber), d) SUMO-sfGFP(wild-type). The masses were determined by ESI-MS of the intact proteins (**Figure S60-S88**).

| ncAA | [mM] | E. coli strain^[a]^ | PylRS construct | reporter | protein yield [mg/L]^[b]^ | protein yield [mg/L]^[b]^ | protein yield [mg/L]^[b]^ |
| --- | --- | --- | --- | --- | --- | --- | --- |
|  |  |  |  |  | Sample 1 | Sample 2 | Sample 3 |
| **2** | 0.05 | BL21 | Mm | a^[c]^ | 18.5 | 16.2 | 19.1 |
| **2** | 0.05 | BL21 | Mbur | a^[c]^ | 20 | 23.7 | 24.9 |
| **2** | 1 | BL21 | Mm | a | 97.2 | 85.4 | 80.3 |
| **2** | 1 | BL21 | Mbur | a | 123.6 | 108.8 | 127.1 |
| **3** | 0.05 | BL21 | Mm | a^[c]^ | 4.3 | 3.7 | 4.9 |
| **3** | 0.05 | BL21 | Mbur | a^[c]^ | 11.2 | 12.1 | 9.8 |
| **3** | 1 | BL21 | Mm | a | 33 | 28.5 | 25.2 |
| **3** | 1 | BL21 | Mbur | a | 84.9 | 95.8 | 88.3 |
| **9** | 3 | BL21 | Mm(N346A:C348A) | a | 11.9 | 8.7 | 7.6 |
| **9** | 3 | BL21 | Mbur(N308A:C310A) | a | 19.9 | 23.4 | 25.8 |
| **11** | 3 | BL21 | Mm(N346A:C348A) | a | 26.4 | 29.3 | 21.2 |
| **11** | 3 | BL21 | Mbur(N308A:C310A) | a | 52 | 59.4 | 58.1 |
| **13** | 1 | BL21 | Mm(N346A:C348A) | a | 4.6 | 3.7 | 3.9 |
| **13** | 1 | BL21 | Mbur(N308A:C310A) | a | 15.1 | 18.9 | 17.7 |
| **8** | 3 | BL21 | Mm(N346A:C348A) | a | 30.2 | 33.6 | 25.4 |
| **8** | 3 | BL21 | Mbur(N308A:C310A) | a | 51.1 | 58.4 | 60.3 |
| **38** | 0.3 | B-95.ΔA | SmbP-Mb(N313W:W382T) | d | 89.8 | 97.6 | 101.3 |
| **38** | 0.3 | B-95.ΔA | SmbP-Mb(N313W:W382T) | a | 42.2 | 40.1 | 46.9 |
| **38** | 0.3 | B-95.ΔA | SmbP-Mb(N313W:W382T) | b | 13.8 | 11.4 | 9.9 |
| **38** | 0.3 | B-95.ΔA | SmbP-Mb(N313W:W382T) | c | 6.7 | 7.5 | 4.2 |
| **38** | 0.3 | B-95.ΔA | Mbur(C310W:W379T) | d | 113.2 | 118.3 | 108.6 |
| **38** | 0.3 | B-95.ΔA | Mbur(C310W:W379T) | a | 86.2 | 98.9 | 90.1 |
| **38** | 0.3 | B-95.ΔA | Mbur(C310W:W379T) | b | 69.1 | 74.2 | 63.4 |
| **38** | 0.3 | B-95.ΔA | Mbur(C310W:W379T) | c | 38.6 | 33.7 | 40.9 |
| **35** | 1 | B-95.ΔA | SmbP-Mb(N313W:W382T) | a | 60.1 | 54.2 | 52.6 |
| **35** | 1 | B-95.ΔA | SmbP-Mb(N313W:W382T) | b | 19.6 | 24.7 | 18.8 |
| **35** | 1 | B-95.ΔA | SmbP-Mb(N313W:W382T) | c | 10.9 | 8.1 | 11.4 |
| **35** | 1 | B-95.ΔA | Mbur(C310W:W379T) | a | 104.9 | 97.6 | 92.3 |
| **35** | 1 | B-95.ΔA | Mbur(C310W:W379T) | b | 59.3 | 65.8 | 57.9 |
| **35** | 1 | B-95.ΔA | Mbur(C310W:W379T) | c | 41 | 44.3 | 37.8 |

[a] All DE3. [b] Yield per liter of cell culture; sfGFP absorption was measured directly in the elution fraction before dialysis.

## Correlation between small-scale fluorescence measurements and shake- flask sfGFP yields

To validate the predictive value of small-scale fluorescence assays, isolated sfGFP yields from shake-flask cultures were correlated with fluorescence measurements obtained in microplate experiments (**Figure S97**).

Across different strains, substrates, and PylRS variants, a strong linear relationship was observed between normalized fluorescence and isolated protein yield, with R² values ranging from 0.86 to 0.98. These results demonstrate that small-scale fluorescence measurements provide a reliable proxy for protein production at preparative scale.

Although minor deviations from linearity are observed in some conditions, the overall correlation supports the use of high-throughput fluorescence screening as an effective predictor of volumetric protein yield.


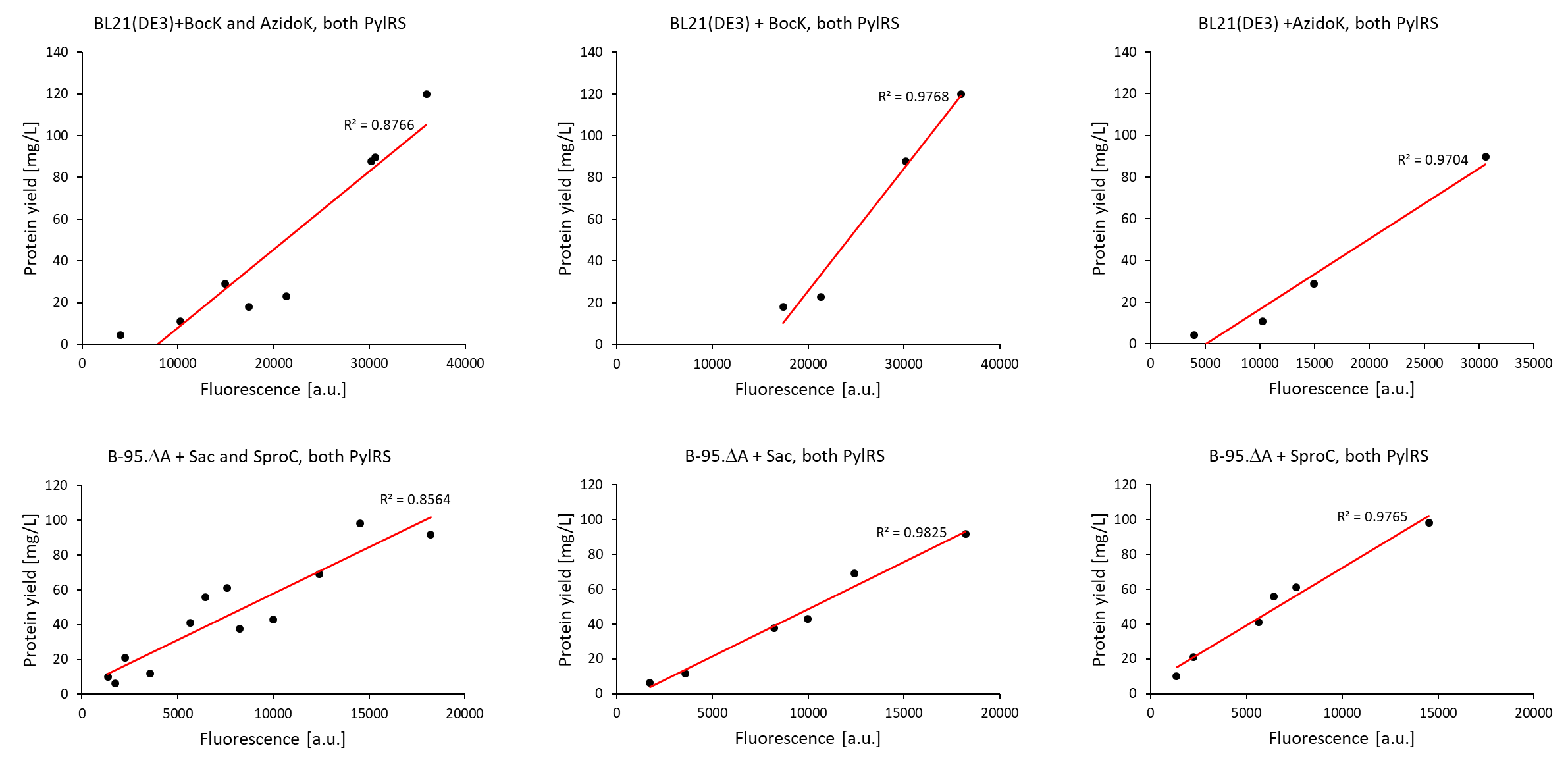


**Figure S97.** Correlation between small-scale fluorescence measurements and isolated sfGFP protein yields. Fluorescence values (a.u.) were obtained from microplate-based assays, and protein yields (mg/L) were determined from shake-flask cultures. Linear regression fits are shown in red, and coefficients of determination (R²) are indicated in each panel.

## Flow Cytometry Dot plots

Representative flow cytometry dot plots corresponding to temperature-dependent sfGFP(5× amber) expression are shown in **Figures S98–S100**. These plots illustrate the gating strategy and population shifts underlying the median fluorescence intensity (MFI) values reported in the main text and Supplementary figures.

At 18 °C, extended cultivation (48 h and 72 h) results in increased fluorescent subpopulations compared to 37 °C after 24 h, consistent with the temperature-dependent expression trends discussed in the manuscript.

**Figure S101** provides representative dot plots from the split-GFP assays used to quantify intracellular PylRS abundance across variants. The distributions confirm distinct fluorescence populations corresponding to differences in expression levels and support the quantitative MFI-based comparisons presented in **Figure S10**.


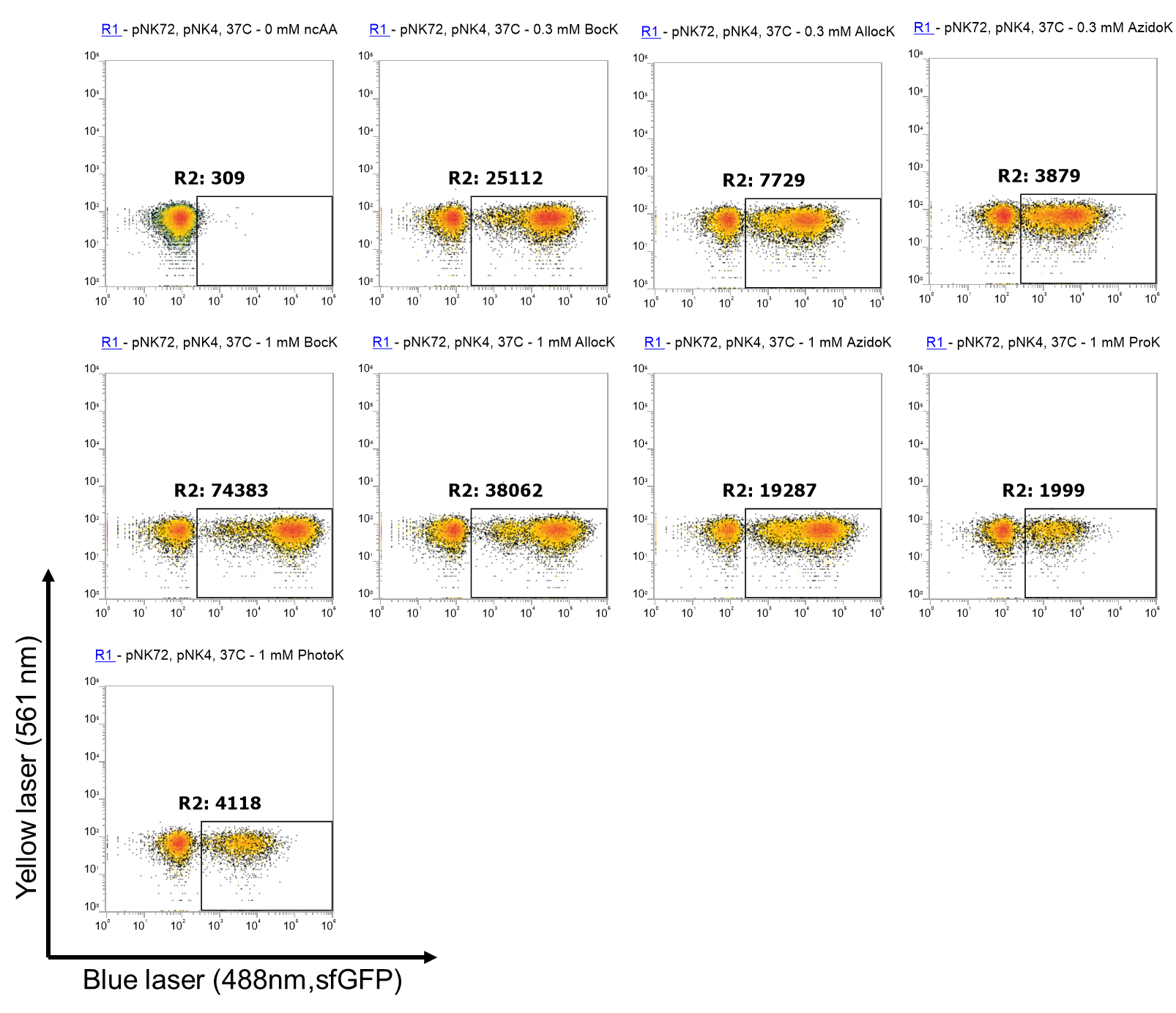


**Figure S98.** Representative flow cytometry dot plots of temperature dependent expression of sfGFP(5x amber) at 37°C after 24h using Mbur. pNK72 encodes Mbur, and pNK4 encodes sfGFP(5× amber).


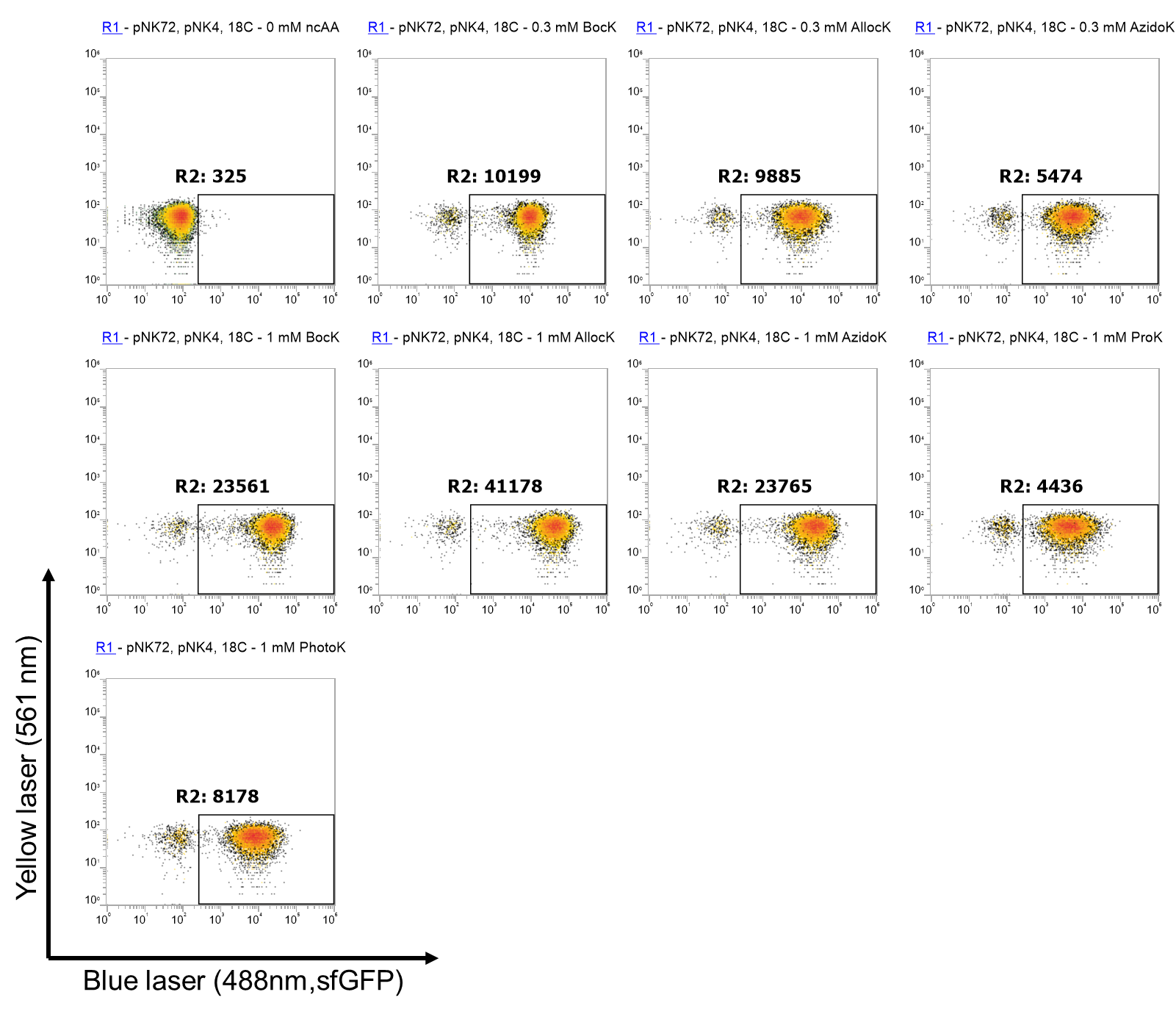
**Figure S99.** Representative flow cytometry dot plots of the temperature dependent expression of sfGFP(5x amber) at 18°C after 48h using Mbur. pNK72 encodes Mbur, and pNK4 encodes sfGFP(5× amber) .


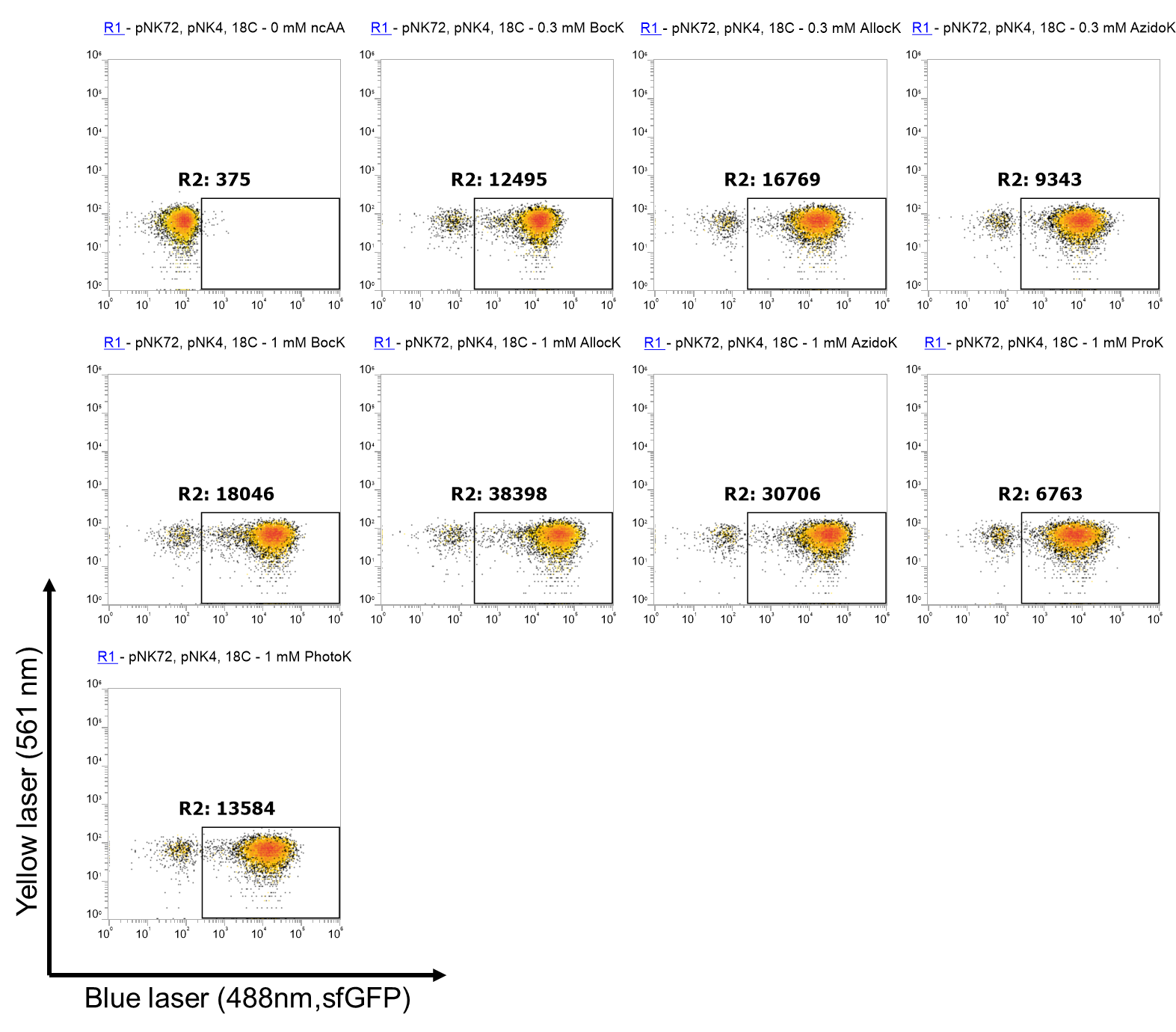


**Figure S100.** Representative flow cytometry dot plots of temperature-dependent expression of sfGFP(5x amber) at 18°C after 72h using Mbur. pNK72 encodes Mbur, and pNK4 encodes sfGFP(5× amber) .


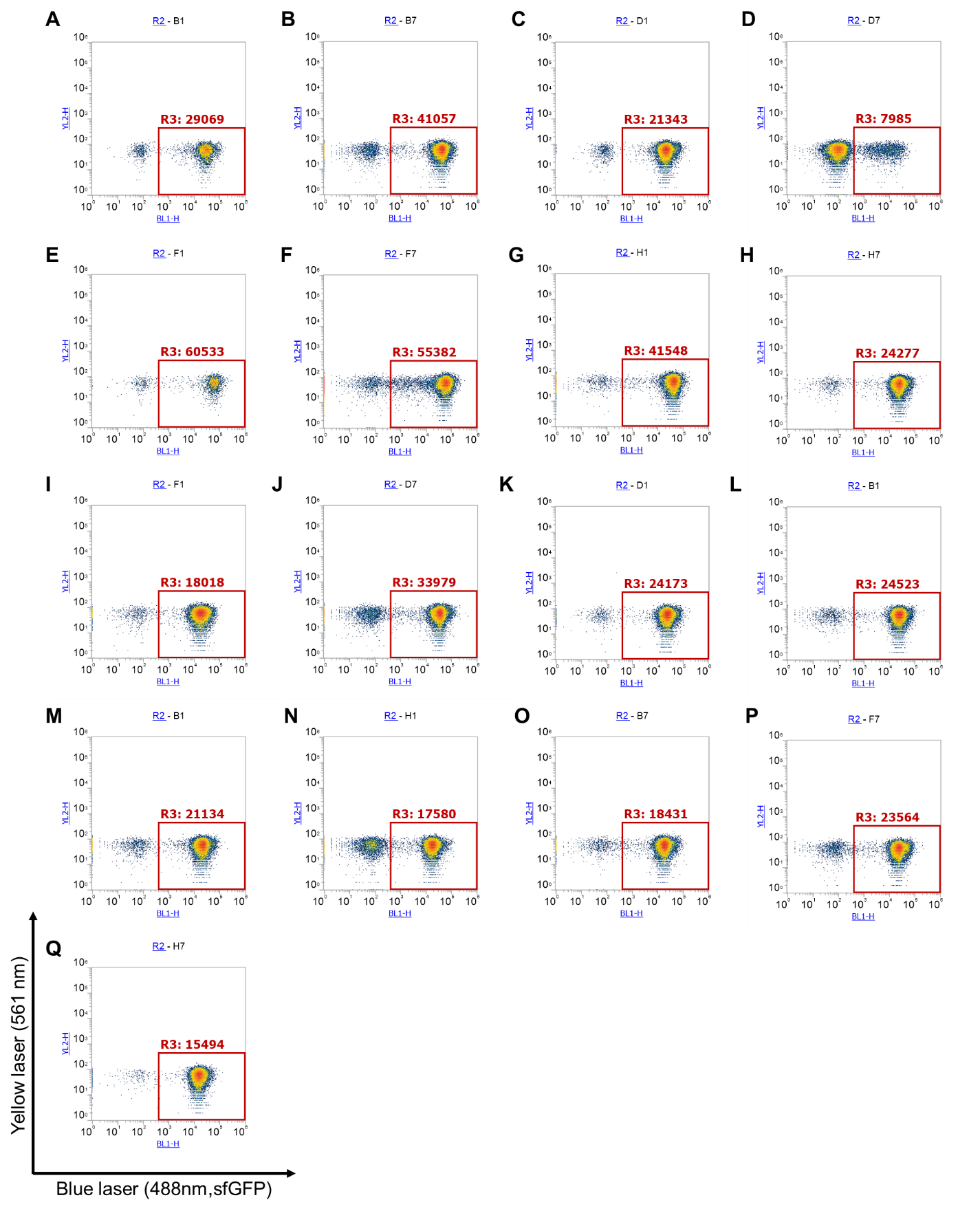


**Figure S101.** Representative dot plots from split-GFP assays for selected variants: **A**) 1R26, **B**) G1, **C**) H5, **D**) I2, **E**) Lum1, **F**) Sheng, **G**) Term, **H**) Alv, **I**) Mp, **J**) Ml, **K**) Mhal, **L**) Mbur, **M**) Mm, **N**) SmbP-Mb, **O**) Mt, **P**) Mt(TM-1), **Q**) Mb.

## Equations used to calculate the Promiscuity Score

This metric reflects how uniformly a PylRS variant activates multiple substrates relative to its best-performing substrate.

To compute the normalized mean activity (*m*) across n=5 substrates we first define:

$m=\frac{1}{n}\sum_{i=0}^{n} \frac{x_{i}}{max(x_{1},\ldots,x_{n})}$ (eq. 1)

where x_i_ represents the fluorescence value (from the plate reader, Fluorescence/OD_600_) obtained for substrate *i*, and max (x_1_,…,x_n_) is the highest value observed among the tested substrates.

This equation would result in $\frac{1}{n}=\frac{1}{5}= 0.2$ for a perfectly specific enzyme. To make the score more intuitive we rescaled it to 0-100%.

To rescale the normalized mean to the interval 0-1 we apply:

$p=\frac{m-\frac{1}{n}}{1-\frac{1}{n}}$ (eq. 2)

For n=5, this simplifies to:

$$p= \frac{m-0.2}{0.8}$$

The promiscuity score (in %) is calculated as

Promiscuity score $=100 xp$.

## OGT References and Source Organisms for +N PylRS Variants Used in Experimental and *In Silico* Analyses

The +N PylRS variants analyzed in this study were derived from publicly available genome sequences of methanogenic archaea. Genomic data were retrieved from NCBI or GenBank databases using the accession numbers listed in **Table S3**. Corresponding tRNA^Pyl^ sequences were extracted from the same genome assemblies.

**Table S3: OGT references and Genome assemblies used for extraction of PylRS and tRNA^Pyl^ sequences**

| Organism | PylRS abbreviation | reference | Strain | Accession number |
| --- | --- | --- | --- | --- |
| *Methanimicrococcus blatticola* |  | Sprenger et al.^19^ | DSM 13328 | GCF_004363215.1 |
| *Methanococcoides alaskense* | Mala | Singh et al.^20^ | DSM 17273 | SAMN18250245 |
| *Methanococcoides burtonii* | Mbur | Allen et al.^21^ | DSM 6242 | GCF_000013725.1 |
| *Methanococcoides methylutens* |  | Sowers et al. ^22^ | DSM 2657 | GCF_000765475.1 |
| *Methanococcoides vulcani* |  | L’Haridon et al.^23^ | SLH 33 | GCF_900111645.1 |
| *Methanohalobium evestigatum* |  | Zhilina et al.^24^ | Z-7303 | GCF_000196655.1 |
| *Methanohalophilus euhalobius* |  | Guan et al.^25^ | WG1_MB | SAMN08777283 |
| *Methanohalophilus halophilus* | Mhal | Guan et al.^25^ | Z-7982 | GCF_001889405.1 |
| *Methanohalophilus levihalophilus* |  | Katayama et al.^26^ | DSM 28452 | GCF_017874375.1 |
| *Methanohalophilus mahii* |  | Guan et al.^25^ | DSM 5219 | GCF_000025865.1 |
| *Methanohalophilus portucalensis* |  | Guan et al.^25^ | FDF-1T | GCF_002761295.1 |
| *Methanohalophilus profundi* |  | L’Haridon et al.^27^ | SLHTYRO | GCF_004137855.1 |
| *Methanolobus bombayensis* |  | Kadam et al.^28^ | DSM 7082 | GCF_017873415.1 |
| *Methanolobus halotolerans* |  | Shen et al.^29^ | SY-01 | GCF_004745425.1 |
| *Methanolobus profundi* |  | Mochimaru et al.^30^ | Mob M | GCF_900114835.1 |
| *Methanolobus psychrophilus* | Mp | Zhang et al.^31^ | R15 | GCF_000306725.1 |
| *Methanolobus psychrotolerans* |  | Chen et al.^32^ | YSF-03 | GCF_002243045.1 |
| *Methanolobus tindarius* |  | König et al.^33^ | DSM 2278 | GCF_000504205.1 |
| *Methanolobus vulcani* |  | Kadam et al.^34^ | PL 12/M | GCF_900100715.1 |
| *Methanolobus zinderi* |  | Doerfert et al.^35^ | DSM 21339 | GCF_013388255.1 |
| *Methanomethylovorans hollandica* |  | Lomans et al.^36^ | DSM 15978 | GCF_000328665.1 |
| *Methanosalsum zhilinae* |  | Sorokin et al.^37^ | DSM 4017 | GCF_000217995.1 |
| *Methanosarcina acetivorans* |  | Sowers et al.^38^ | C2A | GCF_000007345.1 |
| *Methanosarcina barkeri* | Mb | Maestrojuán et al^39^. | 227 | GCF_000970065.1 |
| *Methanosarcina flavescens* |  | Kern et al.^40^ | E03.2 | GCF_001304615.2 |
| *Methanosarcina horonobensis* |  | Shimizu et al.^41^ | HB-1 | GCF_000970285.1 |
| *Methanosarcina lacustris* | Ml | Simankova et al.^42^ | Z-7289 | GCF_000970265.1 |
| *Methanosarcina mazei* | Mm | Maestrojuán et al^39^. | S-6 | GCF_000007065.1 |
| *Methanosarcina siciliae* |  | Ni et al.^43^ | T4/M | GCF_000970085.1 |
| *Methanosarcina soligelidi* |  | Wagner et al.^44^ | SMA-21 | GCF_000744315.1 |
| *Methanosarcina spelaei* |  | Ganzert et al.^45^ | MC-15 | GCF_002287235.1 |
| *Methanosarcina thermophila* | Mt(TM-1) | Zinder et al.^46^ | TM-1 | GCF_000969885.1 |
| *Methanosarcina vacuolata* |  | Maestrojuán et al^39^. | Z-761 | GCF_000969905.1 |

For details regarding the selection and annotation of the Methanosarcina thermophila PylRS sequence (*Mt*PylRS), see Supplementary Section **1.3**.

The ΔN variants were adopted from a previously published study^18^. The corresponding sequences are provided in the Supplementary sequence Excel sheet.

The selected organisms represent phylogenetically diverse members of the Methanosarcinales and related orders, enabling comparative analysis of sequence conservation and structural variability.

# Materials

## Non-Canonical Amino Acids

Unless indicated otherwise, all standard chemicals were purchase from Carl Roth GmbH (Karlsruhe, Germany), Merck (Waltham, MA, USA), VWR International GmbH (Waltham, MA, USA) or Sigma-Aldrich (Taufkirchen, Germany).

**Table S4. Amino acids used in this work.**

| **No.** | **Name** | **Abbreviation** | **Cas No.** | **Company** |
| --- | --- | --- | --- | --- |
| **1** | *N^ε^*‑Allyloxycarbonyl-l-lysine | AllocK | 6298-03-9 | Fluorochem |
| **2** | *N^ε^*‑tert‑Butoxycarbonyl‑l-lysine | BocK | 2418-95-3 | Budisa Group |
| **3** | *N^ε^*‑Propargyloxycarbonyl-l-lysine | ProK | 1428330-91-9 | Iris Biotech |
| **4** | *N^ε^*‑((2‑Azidoethoxy)carbonyl)-l-lysine | AzidoK | 1994331-17-7 | Iris Biotech |
| **5** | 3’-azibutyl-N^ε^-carbamoyl-l-lysine | PhotoK | 1253643-88-7 | Iris Biotech |
| **6** | *N^ε^*‑benzyloxycarbonyl-l-lysine | BenzK | 1155-64-2 | TCI Deutschland |
| **7** | *O*-methyl-l-tyrosine | O-methyl-Y | 6230-11-1 | Fluorochem |
| **8** | *O*-tert-butyl-l-tyrosine | O‑tert-Butyl-Y | 18822-59-8 | Fluorochem |
| **9** | *O*-propargyl-l-tyrosine | O-prop-Y | 610794-20-2 | Iris Biotech |
| **10** | 4-azido-l-phenylalanine | azido-F | 33173-53-4 | Fluorochem |
| **11** | *O*-allyl-l-tyrosine | O-allyl-Y | 107903-42-4 | Iris Biotech |
| **12** | 4-cyano-l-phenylalanine | cyano-F | 167479-78-9 | Alfa Aesar |
| **13** | *O*-CF_3_-l-tyrosine | O-CF_3_-Y | 921609-34-9 | Fluorochem |
| **14** | 4‑ethynyl-l-phenylalanine | ethynyl-F | 278605-15-5 | Sigma-Aldrich (Merck) |
| **15** | (S)-2-amino-3-(3-(hydroxymethyl)-4-nitrophenyl)propanoic acid | p-oNB-alanin |  | Budisa Group |
| **16** | 4-benzoyl-l-phenylalanin | Bpa | 104504-45-2 | Bachem AG |
| **17** | *o*‑(2‑nitrobenzyl)-3,4-dihydroxyphenylalanine | *m*-oNB-Dopa |  | Budisa Group |
| **18** | Sulfotyrosine | sTyr | 956-46-7 | Bachem |
| **19** | (S)-2-Amino-3-(4-((fluorosulfonyl)oxy)phenyl)propanoic acid hydrochloride | FSY | 2227199-79-1 | A2B Chem LLC |
| **20** | O-Phospho-L-tyrosine | pTyr | 21820-51-9 | TCI Chemicals |
| **21** | (S)-2-Amino-3-(4-boronophenyl)propanoic acid | 4-B(OH)-Phe | 76410-58-7 | AmBeeed |
| **22** | 2-Amino-3-(4-(carboxymethyl)phenyl)propanoic acid hydrochloride | CPF | 1803572-24-8 | AmBeeed |
| **23** | (*S*)-2-aminobutyric acid | C4 | 1492-24-6 | TCI Deutschland |
| **24** | (*S*)-2-aminopentanoic acid | C5 | 6600-40-4 | TCI Deutschland |
| **25** | (*S*)-2-aminohexanoic acid | C6 | 327-57-1 | TCI Deutschland |
| **26** | (*S*)-2-aminoheptanoic acid | C7 | 44902-02-5 | Fluorochem |
| **27** | (*S*)-2-aminooctanoic acid | C8 | 116783-26-7 | Fluorochem |
| **28** | (*S*)‑2‑aminopent-4-enoic acid | C5 alken | 16338-48-0 | Fluorochem |
| **29** | (*S*)-2-aminohex-5-enoic acid | C6 alken | 90989-12-1 | Fluorochem |
| **30** | (*S*)-2-amino-3-azidopropanoic acid hydrochloride | azido-ala | 105661-40-3 | Iris Biotech |
| **31** | (*S*)-2-amino-4-azidobutanoic acid hydrochloride | AHA | 942518-29-8 | Carl Roth |
| **32** | (*S*)-2-amino-5-azidopentanoic acid hydrochloride | azido-ornithin | 1782935-10-7 | Iris Biotech |
| **33** | (*S*)‑2‑aminopent‑4‑ynoic acid | propG | 23235-01-0 | Fluorochem |
| **34** | (*S*)-2-aminohex-5-ynoic acid | Hpg | 98891-36-2 | Toronto Research Chemicals |
| **35** | (*S*)-2-aminohept-6-ynoic acid | Bis-Hpg | 835627-45-7 | Chiralix |
| **36** | (*S*)-2-amino-4-methylpent-4-enoic acid | 4,5-DHL | 87392-13-0 | Fluorochem |
| **37** | (*S*)-2-amino-3-cyanopropanoic acid | CA | 6232-19-5 | Iris Biotech |
| **38** | (*S*)-2-amino-4-cyanobutanoic acid | CHA | 6232-22-0 | Iris Biotech |
| **39** | (*S*)-2-amino-3-cyclopropylpropanoic acid | cyclo-ala | 102735-53-5 | Fluorochem |
| **40** | *S*-propargyl-l-cystein | SproC | 3262-64-4 | Fluorochem |
| **41** | l-ethionine | Eth | 13073-35-3 | Sigma-Aldrich (Merck) |
| **42** | l-methionine sulfoxide | Met-sulfoxide | 3226-65-1 | Sigma-Aldrich (Merck) |
| **43** | *S*‑allyl‑l‑cystein | Sac | 21593-77-1 | TCI Deutschland |

## Oligonucleotides

All oligonucleotides were purchased from Sigma-Aldrich (Taufkirchen, Germany), resuspended in ddH_2_O to a final concentration of 100 µM and stored at ‑20 °C. Working concentration of primers were adjusted to 10 µM. Primers shorter than 50 bp were generally purchased in desalted form. Primers between 50-80 bp were ordered in in cartridge-purified form, while longer ones were obtained in HPLC-purified grade. Primers are not listed because far over hundred were used and the utility of this information is limited at best.

# References

1. Herring, S. *et al.* The amino-terminal domain of pyrrolysyl-tRNA synthetase is dispensable in vitro but required for in vivo activity. *FEBS Lett.* **581**, 3197–3203 (2007).

2. Mirdita, M. *et al.* ColabFold: making protein folding accessible to all. *Nat. Methods* **19**, 679–682 (2022).

3. Jumper, J. *et al.* Highly accurate protein structure prediction with AlphaFold. *Nature* **596**, 583–589 (2021).

4. Jumper, J. & Hassabis, D. Protein structure predictions to atomic accuracy with AlphaFold. *Nat. Methods* **19**, 11–12 (2022).

5. Theillet, F.-X. *et al.* The alphabet of intrinsic disorder: Act like a Pro: On the abundance and roles of proline residues in intrinsically disordered proteins. *Intrinsically Disord. Proteins* **1**, e24360 (2014).

6. Ukmar-Godec, T. *et al.* Lysine/RNA-interactions drive and regulate biomolecular condensation. *Nat. Commun.* **10**, 2909 (2019).

7. Jiang, H.-K. *et al.* Linker and N-Terminal Domain Engineering of Pyrrolysyl-tRNA Synthetase for Substrate Range Shifting and Activity Enhancement. *Front. Bioeng. Biotechnol.* **8**, 1–14 (2020).

8. Xi, Z., Liu, L. & Davis, C. C. The Impact of Missing Data on Species Tree Estimation. *Mol. Biol. Evol.* **33**, 838–860 (2016).

9. Violot, S. *et al.* Structure of a Full Length Psychrophilic Cellulase from Pseudoalteromonas haloplanktis revealed by X-ray Diffraction and Small Angle X-ray Scattering. *J. Mol. Biol.* **348**, 1211–1224 (2005).

10. Garsoux, G., Lamotte, J., Gerday, C. & Feller, G. Kinetic and structural optimization to catalysis at low temperatures in a psychrophilic cellulase from the Antarctic bacterium Pseudoalteromonas haloplanktis. *Biochem. J.* **384**, 247–253 (2004).

11. Kavran, J. M. *et al.* Structure of pyrrolysyl-tRNA synthetase, an archaeal enzyme for genetic code innovation. *Proc. Natl. Acad. Sci. U. S. A.* **104**, 11268–11273 (2007).

12. Nozawa, K. *et al.* Pyrrolysyl-tRNA synthetase-tRNAPyl structure reveals the molecular basis of orthogonality. *Nature* **457**, 1163–1167 (2009).

13. Suzuki, T. *et al.* Crystal structures reveal an elusive functional domain of pyrrolysyl-tRNA synthetase. *Nat. Chem. Biol.* **13**, 1261–1266 (2017).

14. Meyer, A. J., Segall-Shapiro, T. H., Glassey, E., Zhang, J. & Voigt, C. A. Escherichia coli “Marionette” strains with 12 highly optimized small-molecule sensors. *Nat. Chem. Biol.* **15**, 196–204 (2019).

15. Cabantous, S., Terwilliger, T. C. & Waldo, G. S. Protein tagging and detection with engineered self-assembling fragments of green fluorescent protein. *Nat. Biotechnol.* **23**, 102–107 (2005).

16. Chen, X., Zaro, J. L. & Shen, W.-C. Fusion protein linkers: Property, design and functionality. *Adv. Drug Deliv. Rev.* **65**, 1357–1369 (2013).

17. Koch, N. G., Goettig, P., Rappsilber, J. & Budisa, N. Engineering Pyrrolysyl-tRNA Synthetase for the Incorporation of Non-Canonical Amino Acids with Smaller Side Chains. *Int. J. Mol. Sci.* **22**, 11194 (2021).

18. Beattie, A. T., Dunkelmann, D. L. & Chin, J. W. Quintuply orthogonal pyrrolysyl-tRNA synthetase/tRNAPyl pairs. *Nat. Chem.* **15**, 948–959 (2023).

19. Sprenger, W. W., van Belzen, M. C., Rosenberg, J., Hackstein, J. H. P. & Keltjens, J. T. Methanomicrococcus blatticola gen. nov., sp. nov., a methanol- and methylamine-reducing methanogen from the hindgut of the cockroach Periplaneta americana. *Int. J. Syst. Evol. Microbiol.* **50**, 1989–1999 (2000).

20. Singh, N., Kendall, M. M., Liu, Y. & Boone, D. R. Isolation and characterization of methylotrophic methanogens from anoxic marine sediments in Skan Bay, Alaska: description of Methanococcoides alaskense sp. nov., and emended description of Methanosarcina baltica. *Int. J. Syst. Evol. Microbiol.* **55**, 2531–2538 (2005).

21. Allen, M. A. *et al.* The genome sequence of the psychrophilic archaeon, Methanococcoides burtonii: The role of genome evolution in cold adaptation. *ISME J.* **3**, 1012–1035 (2009).

22. Sowers, K. R. & Ferry, J. G. Isolation and Characterization of a Methylotrophic Marine Methanogen, Methanococcoides methylutens gen. nov., sp. nov. *Appl. Environ. Microbiol.* **45**, 684–690 (1983).

23. L’Haridon, S., Chalopin, M., Colombo, D. & Toffin, L. Methanococcoides vulcani sp. nov., a marine methylotrophic methanogen that uses betaine, choline and N,N-dimethylethanolamine for methanogenesis, isolated from a mud volcano, and emended description of the genus Methanococcoides. *Int. J. Syst. Evol. Microbiol.* **64**, 1978–1983 (2014).

24. Zhilina, T. N. & Merkel, A. Y. Methanohalobium. in *Bergey’s Manual of Systematics of Archaea and Bacteria* 1–6 (Wiley, 2019). doi:10.1002/9781118960608.gbm00515.pub2.

25. Guan, Y. *et al.* Comparative Genomics of the Genus Methanohalophilus, Including a Newly Isolated Strain From Kebrit Deep in the Red Sea. *Front. Microbiol.* **10**, 1–11 (2019).

26. Katayama, T. *et al.* Methanohalophilus levihalophilus sp. nov., a slightly halophilic, methylotrophic methanogen isolated from natural gas-bearing deep aquifers, and emended description of the genus Methanohalophilus. *Int. J. Syst. Evol. Microbiol.* **64**, 2089–2093 (2014).

27. L’Haridon, S. *et al.* Methanohalophilus profundi sp. nov., a methylotrophic halophilic piezophilic methanogen isolated from a deep hypersaline anoxic basin. *Syst. Appl. Microbiol.* **43**, 126107 (2020).

28. KADAM, P. C., RANADE, D. R., MANDELCO, L. & BOONE, D. R. Isolation and Characterization of Methanolobus bombayensis sp. nov., a Methylotrophic Methanogen That Requires High Concentrations of Divalent Cations. *Int. J. Syst. Bacteriol.* **44**, 603–607 (1994).

29. Shen, Y. *et al.* Methanolobus halotolerans sp. nov., isolated from the saline Lake Tus in Siberia. *Int. J. Syst. Evol. Microbiol.* **70**, 5586–5593 (2020).

30. Mochimaru, H. *et al.* Methanolobus profundi sp. nov., a methylotrophic methanogen isolated from deep subsurface sediments in a natural gas field. *Int. J. Syst. Evol. Microbiol.* **59**, 714–718 (2009).

31. Zhang, G., Jiang, N., Liu, X. & Dong, X. Methanogenesis from Methanol at Low Temperatures by a Novel Psychrophilic Methanogen, “ Methanolobus psychrophilus ” sp. nov., Prevalent in Zoige Wetland of the Tibetan Plateau. *Appl. Environ. Microbiol.* **74**, 6114–6120 (2008).

32. Chen, S.-C. *et al.* Methanolobus psychrotolerans sp. nov., a psychrotolerant methanoarchaeon isolated from a saline meromictic lake in Siberia. *Int. J. Syst. Evol. Microbiol.* **68**, 1378–1383 (2018).

33. König, H. & Stetter, K. O. Isolation and characterization of Methanolobus tindarius , sp. nov., a coccoid methanogen growing only on methanol and methylamines. *Zentralblatt für Bakteriol. Mikrobiol. und Hyg. I. Abt. Orig. C Allg. Angew. und ökologische Mikrobiol.* **3**, 478–490 (1982).

34. KADAM, P. C. & BOONE, D. R. Physiological Characterization and Emended Description of Methanolobus vulcani. *Int. J. Syst. Bacteriol.* **45**, 400–402 (1995).

35. Doerfert, S. N., Reichlen, M., Iyer, P., Wang, M. & Ferry, J. G. Methanolobus zinderi sp. nov., a methylotrophic methanogen isolated from a deep subsurface coal seam. *Int. J. Syst. Evol. Microbiol.* **59**, 1064–1069 (2009).

36. Lomans, B. P. *et al.* Isolation and Characterization of Methanomethylovorans hollandica gen. nov., sp. nov., Isolated from Freshwater Sediment, a Methylotrophic Methanogen Able To Grow on Dimethyl Sulfide and Methanethiol. *Appl. Environ. Microbiol.* **65**, 3641–3650 (1999).

37. Sorokin, D. Y. & Merkel, A. Y. Methanosalsum. in *Bergey’s Manual of Systematics of Archaea and Bacteria* 1–8 (Wiley, 2018). doi:10.1002/9781118960608.gbm00518.pub2.

38. Sowers, K. R., Baron, S. F. & Ferry, J. G. Methanosarcina acetivorans sp. nov., an Acetotrophic Methane-Producing Bacterium Isolated from Marine Sediments. *Appl. Environ. Microbiol.* **47**, 971–978 (1984).

39. MAESTROJUAN, G. M. & BOONE, D. R. Characterization of Methanosarcina barkeri MST and 227, Methanosarcina mazei S-6T, and Methanosarcina vacuolata Z-761T. *Int. J. Syst. Bacteriol.* **41**, 267–274 (1991).

40. Kern, T., Fischer, M. A., Deppenmeier, U., Schmitz, R. A. & Rother, M. Methanosarcina flavescens sp. nov., a methanogenic archaeon isolated from a full-scale anaerobic digester. *Int. J. Syst. Evol. Microbiol.* **66**, 1533–1538 (2016).

41. Shimizu, S., Upadhye, R., Ishijima, Y. & Naganuma, T. Methanosarcina horonobensis sp. nov., a methanogenic archaeon isolated from a deep subsurface Miocene formation. *Int. J. Syst. Evol. Microbiol.* **61**, 2503–2507 (2011).

42. Simankova, M. V. *et al.* Methanosarcina lacustris sp. nov., a New Psychrotolerant Methanogenic Archaeon from Anoxic Lake Sediments. *Syst. Appl. Microbiol.* **24**, 362–367 (2001).

43. Ni, S. & Boone, D. R. Isolation and Characterization of a Dimethyl Sulfide-Degrading Methanogen, Methanolobus siciliae HI350, from an Oil Well, Characterization of M. siciliae T4/MT, and Emendation of M. siciliae. *Int. J. Syst. Bacteriol.* **41**, 410–416 (1991).

44. Wagner, D., Schirmack, J., Ganzert, L., Morozova, D. & Mangelsdorf, K. Methanosarcina soligelidi sp. nov., a desiccation- and freeze-thaw-resistant methanogenic archaeon from a Siberian permafrost-affected soil. *Int. J. Syst. Evol. Microbiol.* **63**, 2986–2991 (2013).

45. Ganzert, L. *et al.* Methanosarcina spelaei sp. nov., a methanogenic archaeon isolated from a floating biofilm of a subsurface sulphurous lake. *Int. J. Syst. Evol. Microbiol.* **64**, 3478–3484 (2014).

46. ZINDER, S. H., SOWERS, K. R. & FERRY, J. G. NOTES: Methanosarcina thermophila sp. nov., a Thermophilic, Acetotrophic, Methane-Producing Bacterium. *Int. J. Syst. Bacteriol.* **35**, 522–523 (1985).
